# UK Biobank retinal imaging grading: methodology, baseline characteristics and findings for common ocular diseases

**DOI:** 10.1038/s41433-022-02298-7

**Published:** 2022-11-03

**Authors:** Alasdair N. Warwick, Katie Curran, Barbra Hamill, Kelsey Stuart, Anthony P. Khawaja, Paul J. Foster, Andrew J. Lotery, Michael Quinn, Savita Madhusudhan, Konstantinos Balaskas, Tunde Peto, UKBB Eye and Vision Consortium, N. Allen, T. Aslam, D. Atan, S. Barman, J. Barrett, P. Bishop, G. Black, T. Braithwaite, R. Carare, U. Chakravarthy, M. Chan, S. Chua, A. Day, P. Desai, B. Dhillon, A. Dick, A. Doney, C. Egan, S. Ennis, P. Foster, M. Fruttiger, J. Gallacher, D. Garway-Heath, J. Gibson, J. Guggenheim, C. Hammond, A. Hardcastle, S. Harding, R. Hogg, P. Hysi, P. Keane, P. T. Khaw, A. Khawaja, G. Lascaratos, T. Littlejohns, A. Lotery, P. Luthert, T. Macgillivray, S. Mackie, B. Mcguinness, G. Mckay, M. Mckibbin, T. Moore, J. Morgan, R. Oram, E. O’sullivan, C. Owen, P. Patel, E. Paterson, T. Peto, A. Petzold, N. Pontikos, J. Rahi, A. Rudnicka, N. Sattar, J. Self, P. Sergouniotis, S. Sivaprasad, D. Steel, I. Stratton, N. Strouthidis, C. Sudlow, Z. Sun, R. Tapp, D. Thomas, E. Trucco, A. Tufail, A. Viswanathan, V. Vitart, M. Weedon, K. Williams, C. Williams, J. Woodside, M. Yates, J. Yip, Y. Zheng

**Affiliations:** 1 Institute of Cardiovascular Science, University College London, London, UK; 2 Medical Retina Service, Moorfields Eye Hospital NHS Foundation Trust, London, UK; 3 Centre for Public Health, Queen’s University Belfast, Faculty of Medicine Health and Life Sciences, Belfast, UK; 4 Institute of Ophthalmology, University College London, London, UK; 5 NIHR Biomedical Research Centre, Moorfields Eye Hospital NHS Foundation Trust, London, UK; 6 Faculty of Medicine, Clinical and Experimental Sciences, University of Southampton, Southampton, UK; 7 Medical Retina Service, University Hospital Southampton NHS Foundation Trust, Southampton, UK; 8 St. Paul’s Eye Unit, Liverpool University Hospitals NHS Foundation Trust, Liverpool, UK; 9 University of Oxford, Oxford, UK; 10 The University of Manchester, Manchester, UK; 11 University of Bristol, Bristol, UK; 12 Kingston University, London, UK; 13 University of Leeds, Leeds, West Yorkshire UK; 14 St Thomas’ Hospital, London, UK; 15 University of Southampton, Southampton, UK; 16 Queen’s University Belfast, Belfast, UK; 17 Moorfields Eye Hospital, London, UK; 18 UCL Institute of Ophthalmology, London, UK; 19 University of Edinburgh, Edinburgh, UK; 20 University of Dundee, Dundee, UK; 21 Cardiff University, Cardiff, UK; 22 King’s College London, London, UK; 23 University of Liverpool, Liverpool, UK; 24 Leeds Teaching Hospitals NHS Trust, Leeds, West Yorkshire UK; 25 University of Exeter, Exeter, UK; 26 King’s College Hospital, London, UK; 27 St George’s, University of London, London, UK; 28 UCL Institute of Neurology, London, UK; 29 UCL Institute of Child Health, London, UK; 30 University of Glasgow, Glasgow, UK; 31 Newcastle University, Newcastle, UK; 32 Gloucestershire Hospitals NHS Foundation Trust, Gloucester, UK; 33 University of East Anglia, Norwich, UK; 34 University of Cambridge, Cambridge, UK

**Keywords:** Eye diseases, Outcomes research

## Abstract

**Background/objectives:**

This study aims to describe the grading methods and baseline characteristics for UK Biobank (UKBB) participants who underwent retinal imaging in 2009–2010, and to characterise individuals with retinal features suggestive of age-related macular degeneration (AMD), glaucoma and retinopathy.

**Methods:**

Non-mydriatic colour fundus photographs and macular optical coherence tomography (OCT) scans were manually graded by Central Administrative Research Facility certified graders and quality assured by clinicians of the Network of Ophthalmic Reading Centres UK. Captured retinal features included those associated with AMD (≥1 drusen, pigmentary changes, geographic atrophy or exudative AMD; either imaging modality), glaucoma (≥0.7 cup-disc ratio, ≥0.2 cup-disc ratio difference between eyes, other abnormal disc features; photographs only) and retinopathy (characteristic features of diabetic retinopathy with or without microaneurysms; either imaging modality). Suspected cases of these conditions were characterised with reference to diagnostic records, physical and biochemical measurements.

**Results:**

Among 68,514 UKBB participants who underwent retinal imaging, the mean age was 57.3 years (standard deviation 8.2), 45.7% were men and 90.6% were of White ethnicity. A total of 64,367 participants had gradable colour fundus photographs and 68,281 had gradable OCT scans in at least one eye. Retinal features suggestive of AMD and glaucoma were identified in 15,176 and 2184 participants, of whom 125 (0.8%) and 188 (8.6%), respectively, had a recorded diagnosis. Of 264 participants identified to have retinopathy with microaneurysms, 251 (95.1%) had either diabetes or hypertension.

**Conclusions:**

This dataset represents a valuable addition to what is currently available in UKBB, providing important insights to both ocular and systemic health.

## Introduction

An estimated 2.5 million people in the UK are currently living with sight loss and this is projected to increase to 3.5 million by 2050 [1]. Population ageing is leading to substantial increases in the number of individuals affected by age-related sight-impairing conditions such as age-related macular degeneration (AMD) and glaucoma, the commonest causes of irreversible blindness globally [2]. The number of individuals with diabetic retinopathy (DR), a leading cause of blindness in the working age population, is also growing in line with the rising prevalence of diabetes [2–4].

Sight loss can have a profound impact on quality of life, restricting social participation and impairing mental and physical health [5]. The annual cost to the UK economy associated with eye conditions is estimated at £25.2 billion, and this is projected to reach £33.5 billion by 2050 [1]. Timely detection and intervention can prevent sight loss and improve socioeconomic outcomes. Economic modelling by the Fight for Sight charity suggests that reducing the prevalence of AMD, glaucoma and type 2 diabetes-related retinopathy by just 1% each year could save the UK economy £1.2 billion, £325 million and £150 million, respectively, by 2050 [1].

Optical coherence tomography (OCT) scans and fundus colour photographs are non-invasive imaging techniques capable of detecting retinal changes at exquisite resolution. They provide a cost-effective and safe method for diagnosing and monitoring disease progression, and for identifying individuals at risk of developing eye disease who may benefit from early intervention [6]. There is furthermore growing evidence that retinal imaging may yield valuable ocular biomarkers for systemic disorders, including cardiovascular disease and dementia [7].

UK Biobank (UKBB) is a large, prospective population-based cohort study including >500,000 UK residents aged between 37 and 73 years, registered with the National Health Service. UKBB has accrued a wealth of phenotypic, genetic imaging data, including colour fundus photographs and OCT scans obtained for a subset of participants between 2009 and 2010 [8]. The present study details the methods used to manually grade these images and aims to (i) describe the subcohort of participants who attended for retinal imaging (ii) characterise individuals with retinal features suggestive of AMD, glaucoma and retinopathy.

## Methods

### Ethics

This project used data from the UKBB study under approved project number 6507 and 36741. Ethics approval was obtained by the Northwest Multi-centre Research Ethics Committee and our research adhered to the tenets of the Declaration of Helsinki [9]. Informed consent was obtained from all study participants and all participants were free to withdraw from the study at any time.

### Study population

Baseline examinations were carried out at 22 study assessment centres between January 2006 and October 2010. All participants underwent a detailed questionnaire-based interview on demographic, clinical and lifestyle related information. The choices for ethnic background were categorised as White, Mixed, Asian or Asian British, Black or Black British, Chinese, or Other ethnic group. Participant postcode at the time of recruitment was used to determine Townsend Deprivation Index (TDI), based on the corresponding output area from the preceding national census; a higher positive score implies a greater degree of deprivation.

Blood pressure (BP) and heart rate were measured using the HEM70151T digital BP monitor (Omron, Hoofddorp, The Netherlands). Body mass index (BMI) was calculated as weight in kilograms divided by height in metres squared. HbA1c (mmol/mol) quantification was performed using the Bio-Rad Variant II Turbo analyser (Bio-Rad Laboratories, Inc.) which employ a high-performance liquid chromatography (HPLC) method. Triglycerides, total cholesterol, low-density lipoprotein (LDL) and high-density lipoprotein (HDL) cholesterol levels (mmol/L) were measured by GPO-POD, CHO-POD, enzyme protective selection and enzyme immunoinhibition analysis respectively on a Beckman Coulter AU5800. Further details of the overall study protocol and protocols for individual tests are available online (https://biobank.ndph.ox.ac.uk/ukb/index.cgi).

### Ophthalmic assessment

Ophthalmic assessment was performed for a subset of participants between 2009 and 2010 at 6 assessments centres, including visual acuity (LogMAR), refractive error and intraocular pressure (IOP) measurements, as well as ophthalmic imaging [8]. Baseline best corrected visual acuity was measured using a computerised semi-automated system at 3 m. Autorefraction was performed using an RC5000 Auto Refkeratometer (Tomey, Nagoya, Japan). The spherical equivalent was calculated by adding the sum of the spherical power and half of the cylindrical power. Corneal compensated intraocular pressure (IOPcc) was measured using the Ocular Response Analyzer (ORA; Reichert Corp., Philadelphia, PA) and one measurement was taken per eye. Any participants with possible eye infections or previous eye surgery (within 4 weeks) were excluded from having IOP measured. Single field colour fundus photographs (45° field-of-view, centred to include both optic disc and macula) and macular OCT scans were captured using a digital Topcon-1000 integrated ophthalmic camera (Topcon 3D OCT1000 Mark II, Topcon Corp., Tokyo, Japan).

### Grading methods

Colour fundus photographs and OCT scans taken at baseline ophthalmic assessment (2009–2010) were graded by Central Administrative Research Facility certified graders and clinicians of the Network of Ophthalmic Reading Centres UK (NetwORC UK – Belfast, Liverpool, and Moorfields Ophthalmic Image Reading Centres). Grading of retinal images and evaluation of OCT scans were performed using dual monitors. Further information on image quality of colour fundus photographs and OCT scans is provided in Supplementary File 1. Standardised training and certification of all graders was carried out before grading began. Study specific grading forms were designed to capture a variety of retinal features, including those relevant to AMD, DR and glaucoma. Graders were able to record incidental findings of other potentially interesting features as free text; these entries were reduced to a standard set of labels following manual review by NetwORC UK clinicians, senior graders and the UKBB Eye and Vision Consortium. Throughout the grading process, all graders were masked to participant characteristics. A data dictionary of graded features is provided in Supplementary Table  1.

### Quality assurance

Regular clinician review and training sessions were performed throughout the duration of the study at quarterly NetwORC UK meetings to ensure consistency between grader decisions. Continuous quality assurance grading was undertaken, and a ratio of 1–20 images were randomly selected for re-grading, in which case, fundus photographs and corresponding OCT scans were independently graded by two or more graders and disagreements were collectively discussed with arbitration graders (Consultant Ophthalmologists, Reading Centre Directors and senior graders) until there was full consensus on the final grade. Any major discrepancies in grades were highlighted and retraining was provided.

### Ascertainment of ocular and systemic disease status from non-imaging data

Eye and systemic disease status, including AMD, glaucoma, retinopathy, diabetes and hypertension were ascertained from the following diagnostic records: verbal interview responses, linked hospital episode statistics, death register and primary care records. At the time of writing, linked primary care data was available for approximately 45% of the UKBB cohort (~230,000 participants). Clinical code lists were manually curated by ophthalmic specialists (AW, KS, AK, TP) for AMD, glaucoma and DR. For diabetes and hypertension, Read 2 and ICD-10 clinical code lists were minimally adapted from the CALIBER Portal [10] and mapped to Read 3 and ICD-9 equivalents respectively, using the mapping files provided by UK Biobank Resource 592 (https://biobank.ndph.ox.ac.uk/ukb/refer.cgi?id=592). Clinical code lists are provided in Supplementary Table  2.

Self-reported medication history for insulin, antihypertensive medication and cholesterol-lowering medication usage was obtained from baseline touchscreen questionnaire responses. An HbA1c >48 mmol/mol (World Health Organisation (WHO) diagnostic threshold for diabetes) was considered diagnostic of diabetes. Systolic or diastolic blood pressure measurements ≥140 and ≥80 mmHg, respectively, were considered diagnostic of hypertension.

### Imaging-based definitions of suspected ocular disease status

Participants were identified as having suspected AMD, suspected glaucoma or retinopathy (with or without microaneurysms) if the following retinal features were present in either eye:

- Suspected AMD: any drusen, pigmentary changes, geographic atrophy or exudative AMD on either colour fundus photographs or OCT scans.
- Suspected glaucoma: cup-to-disc ratio (CDR) ≥0.7 in either eye, inferior rim notching or thinning, or presence of a disc haemorrhage. These features were identified from examination of colour fundus photographs only [11]. A ≥0.2 CDR difference between right and left eyes was also considered suspicious for glaucoma.
- Retinopathy with microaneurysms: one or more microaneurysms with or without hard exudates, cotton wool spots, intra-retinal microvascular abnormalities, venous beading, pre-retinal/vitreous haemorrhage or neovascularization on colour fundus photographs, or inner retinal changes on OCT.
- Retinopathy without microaneurysms: as for retinopathy with microaneurysms, but in the absence of microaneurysms.

### Statistical analysis

Sociodemographic details, self-reported medication history, ocular, physical and biochemical measurements obtained at baseline were included for analysis, as these would have generally been obtained closest to the date of retinal image acquisition. Repeated blood pressure measurements (automated and/or manual) were summarised by calculating a mean value. Diagnosed ocular and systemic disease status were ascertained from diagnostic codes recorded both at date of attendance for baseline retinal imaging (or 1 January 2010 for participants who did not undergo imaging; Table  1, Supplementary Table  3 and Supplementary Fig.  1(i)), as well as using all health records available at the time of writing (primary care data up to 2016, hospital episode statistics and death register up to 2020; Supplementary Fig.  1(ii)).

**Table 1:** Participant characteristics by attendance for retinal imaging in 2009–2010 (left), and by suspected ocular disease status based on retinal image gradings (right).

	Attended retinal imaging				Retinal grading-defined outcomes			
Characteristic	No, *N* = 433,983	Yes, *N* = 68,514	*p* value	AMD suspected, *N* = 15,176	Glaucoma suspected, *N* = 2184	Retinopathy with MA, *N* = 264	Retinopathy without MA, *N* = 1601	*p* value
Age	58.2 (8.1)	57.3 (8.1)	**<0.001**	59.3 (7.7)	57.8 (8.1)	57.7 (8.1)	59.5 (7.8)	**<0.001**
Sex			0.7					**<0.001**
Female	236,145 (54.4%)	37,230 (54.3%)		8219 (54.2%)	1156 (52.9%)	77 (29.2%)	834 (52.1%)	
Male	197,830 (45.6%)	31,284 (45.7%)		6957 (45.8%)	1028 (47.1%)	187 (70.8%)	767 (47.9%)	
Ethnic background			**<0.001**					**<0.001**
White	411,084 (95.2%)	61,596 (90.6%)		13,638 (90.6%)	1839 (84.8%)	210 (81.4%)	1391 (87.5%)	
Mixed	2358 (0.5%)	600 (0.9%)		137 (0.9%)	18 (0.8%)	6 (2.3%)	14 (0.9%)	
Asian or Asian British	7660 (1.8%)	2222 (3.3%)		506 (3.4%)	100 (4.6%)	23 (8.9%)	75 (4.7%)	
Black or Black British	5848 (1.4%)	2213 (3.3%)		445 (3.0%)	150 (6.9%)	13 (5.0%)	77 (4.8%)	
Chinese	1262 (0.3%)	312 (0.5%)		66 (0.4%)	17 (0.8%)	1 (0.4%)	7 (0.4%)	
Other ethnic group	3483 (0.8%)	1075 (1.6%)		268 (1.8%)	45 (2.1%)	5 (1.9%)	25 (1.6%)	
Townsend Deprivation Index	−1.34 (3.11)	−1.01 (3.00)	**<0.001**	−1.08 (2.97)	−0.85 (3.08)	−0.49 (3.22)	−0.87 (3.08)	**<0.001**
On insulin	4874 (1.1%)	738 (1.1%)	0.3	165 (1.1%)	21 (1.0%)	125 (47.3%)	64 (4.0%)	**<0.001**
On antihypertensive medication	89,738 (20.7%)	14,257 (20.8%)	0.4	3600 (23.7%)	482 (22.1%)	146 (55.3%)	514 (32.1%)	**<0.001**
On cholesterol-lowering medication	74,200 (17.1%)	12,688 (18.5%)	**<0.001**	3219 (21.2%)	436 (20.0%)	155 (58.7%)	446 (27.9%)	**<0.001**
Diabetes diagnosis	24,158 (5.6%)	3826 (5.6%)	0.9	919 (6.1%)	146 (6.7%)	184 (69.7%)	210 (13.1%)	**<0.001**
Hypertension diagnosis	124,679 (28.7%)	18,724 (27.3%)	**<0.001**	4581 (30.2%)	643 (29.4%)	157 (59.5%)	651 (40.7%)	**<0.001**
Age-related macular degeneration diagnosis	1684 (0.4%)	271 (0.4%)	0.8	125 (0.8%)	14 (0.6%)	6 (2.3%)	48 (3.0%)	**<0.001**
Glaucoma diagnosis	6319 (1.5%)	1128 (1.6%)	**<0.001**	318 (2.1%)	189 (8.7%)	11 (4.2%)	55 (3.4%)	**<0.001**
Diabetic retinopathy diagnosis	3852 (0.9%)	678 (1.0%)	**0.009**	181 (1.2%)	29 (1.3%)	132 (50.0%)	102 (6.4%)	**<0.001**

Characteristics were ascertained from self-reported (verbal interview) data at study baseline and linked electronic healthcare records data (primary care, hospital episode statistics, death register) up until the time of retinal imaging, or 1 January 2010, for participants who did not undergo imaging.

Continuous variables are summarised by mean (standard deviation) and compared using one-way ANOVA. Categorical variables are summarised by number (percentage) and compared using Pearson’s *χ*^2^ test.

*AMD* age-related macular degeneration, *MA* microaneurysm. Statistically significant comparisons (*p* < 0.05) are highlighted in bold.

Simple descriptive statistics were presented as mean (standard deviation) for continuous variables and number (percentage) for categorical variables. Student’s *t*-test and one-way ANOVA were used to compare continuous variables for two and more than two groups, respectively. Categorical variables were compared using Pearson’s *χ*^2^ test. Cohen’s kappa coefficient was calculated to assess intergrader agreement for detecting any gradable abnormalities at the patient level, based on a random sample of 525 patients.

Statistical analysis was performed using SPSS (IBM SPSS Statistics for Window, Version 26.0., IBM Corp, Armonk, New York, USA) and R (R for GNU macOS, Version 4.1.0, The R Foundation for Statistical Computing, Vienna, Austria). R packages used included targets, tarchetypes, workflowr, tidyverse, ukbwranglr, codemapper, ggstatsplot, knitr, gtsummary and flextable [12–21].

## Results

**Fig. 1:**
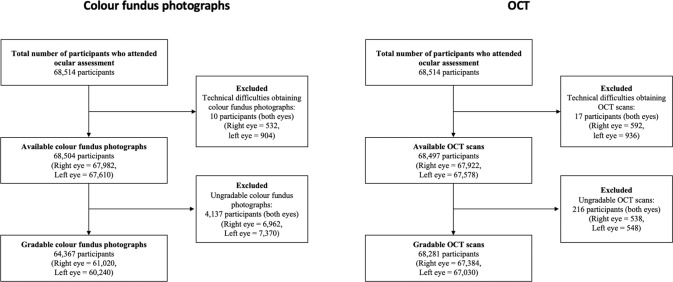
Flowcharts of participants who attended for retinal imaging at ocular assessment. OCT optical coherence tomography.

A total of 68,514 UKBB participants attended for retinal imaging between 2009 and 2010. Of these, 68,504 had colour fundus photographs taken in at least one eye, of which 64,367 (121,260 eyes) had at least one gradable photo. Likewise, 68,497 participants had an available OCT scan for at least one eye, of which 68,281 (134,414 eyes) had at least one gradable scan (Fig.  1). The grading findings for individual retinal features are summarised in Supplementary Table  4. There was moderate intergrader agreement for detecting any abnormalities at the patient level (Cohen’s k = 0.60; *p* < 0.001).

### Comparison with participants who did not attend for retinal imaging

Individuals who attended for retinal imaging were slightly younger and had a higher TDI compared to those who did not (Table  1). A lower proportion of those imaged described their ethnic background as White, while the ratio of male to female participants was similar to those without ocular measurements. Comparing medical records, those who attended for retinal imaging more commonly self-reported taking cholesterol-lowering medication while slightly fewer had a diagnosis of hypertension. The prevalence of diagnosed DR was also marginally lower.

### Ungradable retinal images

In comparison to individuals with gradable retinal images in both eyes, participants with an ungradable OCT scan or colour fundus photographs in at least one eye were older, had a higher TDI, and were more likely to be male and of non-White ethnic background. The prevalence of all considered medical conditions (ascertained from diagnostic records) was also higher, and these individuals were more likely to be taking insulin, cholesterol-lowering or antihypertensive medication (Supplementary Table  3).

### Diagnosed ocular disease

Among participants who underwent retinal imaging, the number of individuals with a diagnostic record of AMD, glaucoma and DR was 271 (0.4%), 1128 (1.6%) and 678 (1.0%), respectively (Table  1). These figures rose to 1205 (1.8%), 2122 (3.1%) and 1435 (2.1%), respectively, when including diagnostic codes recorded after baseline imaging (Supplementary Fig. 1). Within the 3826 participants (5.6% of those imaged) with a diagnosis of diabetes, 672 (17.6%) also had a diagnostic record for DR.

### Suspected ocular disease from retinal imaging

By comparison, retinal features suggestive of AMD and suspected glaucoma were identified in 15,176 (22.2%) and 2184 (3.2%) participants, of whom 125 (0.8%) and 188 (8.6%), respectively, had a diagnostic record of these conditions at the time of retinal imaging (Table  1).

There were 264 (0.4%) and 1601 (2.3%) participants with retinopathy with and without microaneurysms respectively, of whom 132 (50.0%) and 102 (6.4%) had a diagnostic record of DR. Within the 3826 participants with a diagnosis of diabetes, 184 (4.8%) and 210 (5.5%) individuals had evidence of retinopathy with and without microaneurysms, with mean HbA1c measurements of 63 and 57 mmol/mol, respectively. Within 64,688 participants without a diagnosis of diabetes, 80 (0.1%) and 1391 (2.2%) had retinopathy with and without microaneurysms, with a mean HbA1c measurement of 36 mmol/mol in both groups; only 11 individuals from the latter group had an HbA1c above the WHO diagnostic threshold for diabetes.

**Fig. 2:**
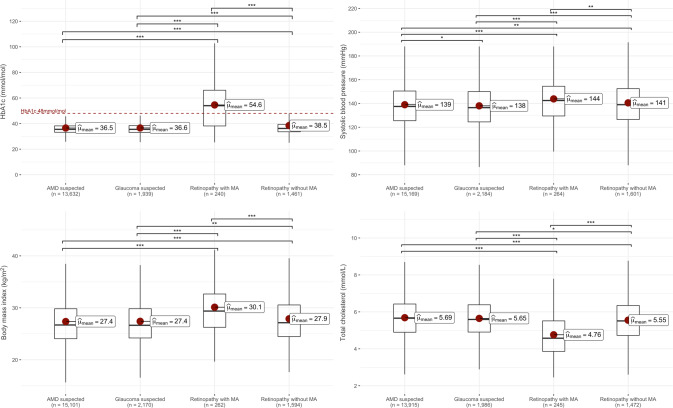
Comparisons of baseline measurements for HbA1c, systolic blood pressure, body mass index and total cholesterol between participants who were identified from retinal imaging to have suspected age-related macular degeneration, glaucoma, or retinopathy with or without microaneurysms. Statistically significant pairwise comparisons (Student’s *t*-test) are indicated by asterisks: *p < 0.05, **p < 0.01, ***p < 0.001. *MA* microaneurysm.

The most striking demographic differences were observed for participants graded to have retinopathy with microaneurysms. Of these 264 individuals, 187 (70.8%) were male, compared with a small majority of females for the other three conditions. This group was also comparatively younger, with the highest TDI and the highest proportion of non-white participants. A diagnosis of diabetes was recorded for 184 individuals (69.7%), 147 (55.7%) had an HbA1c level in the diagnostic range for diabetes (mean 54.6 mmol/mol), and 125 (47.3%) self-reported use of insulin. A diagnosis of hypertension was recorded for 157 (59.5%) individuals, 146 (55.3%) reported taking antihypertensive medication and 194 (73.5%) had blood pressure measurements in the hypertensive range. Overall, 251 (95.1%) of these participants had either diabetes or hypertension (diagnosed or undiagnosed), while 166 (62.9%) had both. Cholesterol-lowering medication usage was reported by 155 (58.7%) and the mean total cholesterol, LDL and HDL cholesterol levels were comparatively lower for these individuals compared with the other groups (Fig.  2 and Supplementary Fig.  2). By comparison, triglycerides levels were slightly higher in this group (1.80 mmol/L compared to 1.67, 1.66 and 1.69 mmol/L in the AMD, glaucoma, and retinopathy without MA groups) (Supplementary Fig.  2).

Similar findings, albeit less pronounced, were observed for individuals with retinopathy without microaneurysms. The proportion of participants with either diabetes or hypertension, or taking insulin, antihypertensive or cholesterol-lowering medication was higher relative to individuals with suspected AMD or glaucoma, but lower in comparison to those with retinopathy with microaneurysms (Table 1). Overall, 1225 (76.5%) of these participants had either diabetes or hypertension (diagnosed or undiagnosed), while 192 (12.0%) had both.

Comparing ocular measures, suspected AMD, suspected glaucoma and retinopathy were all more commonly identified for right eyes than for left eyes (Table  2), reflecting the larger number of left eyes for which retinal images were either unobtainable or ungradable (Fig. 1). Eyes with suspected glaucoma had the highest IOPcc among the diagnostic groups. Eyes with suspected retinopathy with microaneurysms had the lowest visual acuity. Eyes with retinopathy without microaneurysms had a similar average spherical equivalent to those with suspected glaucoma, which was relatively myopic compared to those with retinopathy with microaneurysms or suspected AMD.

**Table 2:** Summary of eye measurements data by ocular disease status, as suspected from retinal grading.

Characteristic	AMD suspected, *N* = 20,979	Glaucoma suspected, *N* = 1438	Retinopathy with MA, *N* = 402	Retinopathy without MA, *N* = 1945	*p* value
Eye					**0.025**
Left eye	10,472 (49.9%)	660 (45.9%)	193 (48.0%)	955 (49.1%)	
Right eye	10,507 (50.1%)	778 (54.1%)	209 (52.0%)	990 (50.9%)	
Visual acuity (LogMAR)	0.04 (0.21)	0.05 (0.21)	0.14 (0.27)	0.13 (0.27)	**<0.001**
IOPcc (mmHg)	16.0 (3.6)	17.2 (3.9)	15.7 (3.8)	16.1 (3.6)	**<0.001**
Spherical equivalent (Dioptres)	−0.1 (3.2)	−1.8 (4.3)	−0.7 (3.0)	−1.4 (4.7)	**<0.001**

Continuous variables are summarised by mean (standard deviation) and compared using Student’s *t*-test and one-way ANOVA for two and more than two groups, respectively. Categorical variables are summarised by number (percentage) and compared using Pearson’s *χ*^2^ test.

*AMD* age-related macular degeneration, *MA* microaneurysm, *IOPcc* corneal compensated intraocular pressure. Statistically significant comparisons (*p* < 0.05) are highlighted in bold.

## Discussion

This study describes the subcohort of 68,514 UKBB participants who attended for retinal imaging (fundus photographs and OCT) in 2009–2010 and the methods for image grading. Drawing on diagnostic records, physical and biochemical measurements, we furthermore characterise individuals identified to have retinal features suggestive of AMD (15,176), glaucoma (2184) and retinopathy with (264) and without (1601) microaneurysms.

The number of participants in this subcohort with a diagnostic record of AMD (any severity) at baseline retinal imaging in 2009–2010 was 271 (prevalence 0.4%). By comparison, Desai et al. previously predicted 271 participants to have either geographic atrophy or neovascular AMD by 2013 [22], suggesting that electronic health records do not capture all cases of AMD. Indeed, retinal imaging identified substantially more suspected AMD cases compared to medical records. According to our grading data, the prevalence of suspected AMD (defined as the presence of one or more drusen, pigmentary changes, geographic atrophy or exudative AMD on retinal colour photographs or OCT scans) was 22.2% (15,176 individuals). A meta-analysis by Wong et al. reported a prevalence of 12.3% for any AMD severity in Europeans [23]. Our definition for AMD was more permissive than commonly used AMD grading criteria [24–26], which could explain the discrepancy. The inclusion of features detected on OCT in our study may have additionally improved sensitivity. Future work will explore more granular AMD definitions derived from the available graded features (Supplementary Table  4). Of note, previous estimates for the prevalence of drusen in adults range from 40.5 to 96.9% [27–31]. It is likely that these studies, which focused primarily on grading drusen, were more sensitive for detecting smaller drusen (<63 µm) in particular.

Patients with suspected AMD were more hyperopic compared to individuals with suspected glaucoma or retinopathy. This is consistent with previous studies where a higher AMD prevalence has been found among individuals with hyperopic eyes compared to emmetropic eyes, and others that have reported a lower risk of AMD among people with myopia [32–36].

The number of individuals with a diagnostic record of glaucoma (1128 at baseline retinal imaging, prevalence 1.6%) was higher than the prediction from Desai et al. (311 predicted for 2013) [22]. The latter prevalence estimate was based on prevalence data from population surveys that employed visual fields assessment, and confirmatory clinician review to confirm the presence of glaucoma. These case diagnoses from surveys are probably more secure than those from healthcare records and self-reported data alone.

In comparison, the number of participants identified as having suspected glaucoma from gradable UKBB fundus photographs was even higher (2184, prevalence 3.2%). These individuals had a higher IOPcc relative to those with suspected AMD and retinopathy. The overall prevalence of open angle glaucoma in the Blue Mountain Eye Study was 3.0% [37]. Lower rates were reported in the Beaver Dam Eye Study (2.1%) and Rotterdam Study (1.1%) [27, 38]. In Ireland, the prevalence of definite open angle glaucoma and suspected glaucoma was 1.88 and 1.05%, respectively [39]. Variances in sampling methodology, cohort age distributions and case definitions for glaucoma may explain the differences in prevalence estimates. Visual field testing was not possible in UKBB. Consequently, the high rates of suspected glaucoma are not surprising. If field testing had been carried out, between 35 and 50% of tests may have been normal, therefore bringing the true glaucoma prevalence into line with that seen under more rigorous survey methodology [11]. Fundus photographs only were used for disc examination; however, multimodal optic disc examination may uncover more glaucomatous discs in future studies.

Approximately one third of patients with diabetes have some degree of retinopathy and one in ten will have sight-threatening disease [3]. Retinopathy is also often detectable in people without diabetes, especially in the presence of hypertension [40]. Retinal microaneurysms are the earliest clinical signs of DR and also often observed with hypertension [40, 41]. We therefore hypothesised that microaneurysms would associate particularly strongly with these two conditions. Strikingly, we found that 95.1% of individuals identified to have retinopathy with microaneurysms had either diabetes or hypertension (diagnosed or undiagnosed), and 62.9% had both.

Among participants with diabetes in the current study, 17.6% had a history of DR recorded in their medical records, while the prevalence of retinopathy with or without microaneurysms detectable on retinal imaging was 10.3%. The prevalence of retinopathy with or without microaneurysms within participants without diabetes was 2.3%. In comparison, the prevalence of DR in the Beaver Dam Eye Study was 36.8%, and was 26.8% in the Tromsø Eye Study [27, 42]. The prevalence of DR in The Age, Gene/Environment Susceptibility-Reykjavik Study (AGES-R) was 27.0%, while the prevalence of retinopathy in people without diabetes was 10.7% [43]. Our findings are lower than expected from previous epidemiological studies, which may reflect that UKBB represents a relatively healthy cohort [42, 44]. Furthermore, the more limited photographic survey performed for UKBB participants (single field) is likely to have reduced sensitivity for retinopathy detection. Indeed, non-mydriatic photography was employed in the Atherosclerosis Risk in Communities Study, where the reported prevalence of retinopathy in people without diabetes was also relatively low at 4% [45].

### Strengths and limitations

This study has several strengths. It is one of the largest prospective cohort studies containing rigorously graded (double grading with adjudication) data from both colour fundus photographs and OCT scans for a range of retinal features. Single field fundus photography without pupil dilation was used to ensure there was a high study-uptake rate by reducing image acquisition time. This approach is less accurate than 7-field imaging however and does mean that some features outside the macula will have been missed [46]. Media opacities and smaller pupillary diameters may partly account for ungradable colour fundus images in 6% of participants, a group who appear to be at higher risk of health problems (Supplementary Table  3). Nonetheless, comparison with diagnostic records, physical and biochemical measurements still clearly demonstrated the potential to detect ocular and systemic disease from available retinal imaging.

There is rapidly growing interest in the use of artificial intelligence (AI) techniques to automate feature extraction from retinal images. For example, deep learning algorithms can extract features from colour fundus photographs to predict cardiovascular risk factors and other systemic biomarkers, including body composition indices and serum creatinine [47, 48]. Despite these impressive advances in AI and deep learning however, further validation is required to gauge their clinical effectiveness. In particular, these algorithms rely on high quality labelled data [49]. We hope that the manual feature-based grading of retinal images from UKBB participants will facilitate the development of AI algorithms and prediction tools in the future.

## Conclusions

In summary, the manual retinal grading findings for this subcohort of participants with colour fundus photos and OCT data provides valuable additional information, complementing the already rich phenotypic and genetic data held by UKBB. By focusing on just a few retinal features, we showcase the potential utility of ocular biomarkers that are readily obtainable from non-invasive ophthalmic imaging. This formidable data resource promises to accelerate research across a range of areas with relevance to both ocular and associated systemic diseases.

## Summary

### What was known before

- Previous population-based studies have assessed the prevalence of retinal conditions such as age-related macular degeneration, glaucoma and diabetic retinopathy using retinal grading data and diagnostic records.
- Ophthalmic imaging data can yield ocular biomarkers for both ophthalmic and systemic disorders.

### What this study adds

- This study presents rigorous retinal grading data (double graded and adjudicated) for 68,514 UK Biobank participants.
- Data were obtained from both colour fundus photographs and optical coherence tomography scans taken between 2009 and 2010.
- The feature-based grading of retinal images from this subcohort complements the phenotypic and genetic data already contained in the UK Biobank, providing important insights to ocular and systemic health.

## Supplementary information

Supplementary tables

Supplementary figure 1

Supplementary figure 2

Supplementary File 1

Supplementary Material

## Data Availability

The manually graded retinal imaging dataset presented in the current study will be available from the UK Biobank for approved researchers to request access.
