# Correction: UK Biobank retinal imaging grading: methodology, baseline characteristics and findings for common ocular diseases

**DOI:** 10.1038/s41433-022-02377-9

**Published:** 2023-01-05

**Authors:** Alasdair N. Warwick, Katie Curran, Barbra Hamill, Kelsey Stuart, Anthony P. Khawaja, Paul J. Foster, Andrew J. Lotery, Michael Quinn, Savita Madhusudhan, Konstantinos Balaskas, Tunde Peto, UKBB Eye and Vision Consortium, N. Allen, T. Aslam, D. Atan, S. Barman, J. Barrett, P. Bishop, G. Black, T. Braithwaite, R. Carare, U. Chakravarthy, M. Chan, S. Chua, A. Day, P. Desai, B. Dhillon, A. Dick, A. Doney, C. Egan, S. Ennis, P. Foster, M. Fruttiger, J. Gallacher, D. Garway-Heath, J. Gibson, J. Guggenheim, C. Hammond, A. Hardcastle, S. Harding, R. Hogg, P. Hysi, P. Keane, P. T. Khaw, A. Khawaja, G. Lascaratos, T. Littlejohns, A. Lotery, P. Luthert, T. Macgillivray, S. Mackie, B. Mcguinness, G. Mckay, M. Mckibbin, T. Moore, J. Morgan, R. Oram, E. O’sullivan, C. Owen, P. Patel, E. Paterson, T. Peto, A. Petzold, N. Pontikos, J. Rahi, A. Rudnicka, N. Sattar, J. Self, P. Sergouniotis, S. Sivaprasad, D. Steel, I. Stratton, N. Strouthidis, C. Sudlow, Z. Sun, R. Tapp, D. Thomas, E. Trucco, A. Tufail, A. Viswanathan, V. Vitart, M. Weedon, K. Williams, C. Williams, J. Woodside, M. Yates, J. Yip, Y. Zheng

**Affiliations:** 1 Institute of Cardiovascular Science, University College London, London, UK; 2 Medical Retina Service, Moorfields Eye Hospital NHS Foundation Trust, London, UK; 3 Centre for Public Health, Queen’s University Belfast, Faculty of Medicine Health and Life Sciences, Belfast, UK; 4 Institute of Ophthalmology, University College London, London, UK; 5 NIHR Biomedical Research Centre, Moorfields Eye Hospital NHS Foundation Trust, London, UK; 6 Faculty of Medicine, Clinical and Experimental Sciences, University of Southampton, Southampton, UK; 7 Medical Retina Service, University Hospital Southampton NHS Foundation Trust, Southampton, UK; 8 St. Paul’s Eye Unit, Liverpool University Hospitals NHS Foundation Trust, Liverpool, UK; 9 University of Oxford, Oxford, UK; 10 The University of Manchester, Manchester, UK; 11 University of Bristol, Bristol, UK; 12 Kingston University, London, UK; 13 University of Leeds, Leeds, West Yorkshire UK; 14 St Thomas’ Hospital, London, UK; 15 University of Southampton, Southampton, UK; 16 Queen’s University Belfast, Belfast, UK; 17 Moorfields Eye Hospital, London, UK; 18 UCL Institute of Ophthalmology, London, UK; 19 University of Edinburgh, Edinburgh, UK; 20 University of Dundee, Dundee, UK; 21 Cardiff University, Cardiff, UK; 22 King’s College London, London, UK; 23 University of Liverpool, Liverpool, UK; 24 Leeds Teaching Hospitals NHS Trust, Leeds, West Yorkshire UK; 25 University of Exeter, Exeter, UK; 26 King’s College Hospital, London, UK; 27 St George’s, University of London, London, UK; 28 UCL Institute of Neurology, London, UK; 29 UCL Institute of Child Health, London, UK; 30 University of Glasgow, Glasgow, UK; 31 Newcastle University, Newcastle, UK; 32 Gloucestershire Hospitals NHS Foundation Trust, Gloucester, UK; 33 University of East Anglia, Norwich, UK; 34 University of Cambridge, Cambridge, UK

**Keywords:** Eye diseases, Outcomes research

Correction to: *Eye* 10.1038/s41433-022-02298-7, published online 03 November 2022

The wrong Supplementary file was originally published with this article; it has now been replaced with the correct file.

The original article has been corrected.

## Supplementary information

Supplementary Table

