# Supplementary figures and images for: UK Biobank retinal imaging grading: methodology, baseline characteristics and findings for common ocular diseases
Source: Eye (Lond). Author manuscript; Available in PMC 2023 Jul 12. (PMC10333328; doi:10.1038/s41433-022-02298-7)

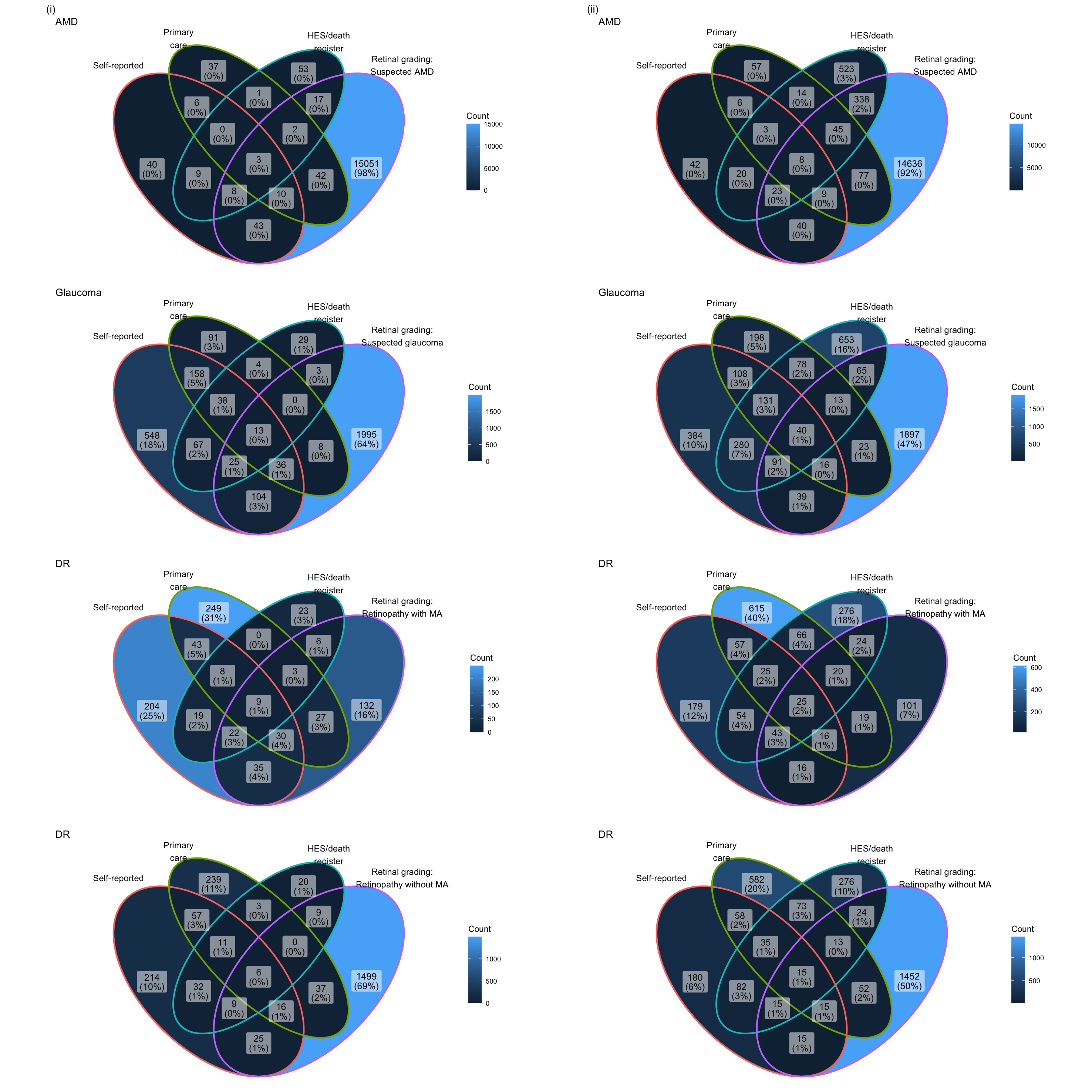

Supplement: sFigure 1 — Numbers of participants who attended for retinal imaging with a diagnostic record of age-related macular degeneration, glaucoma and diabetic retinopathy identified through self-reported (verbal intervew), primary care, hospital episode statistics and death register records and those with suspicious features for these conditions identified from retinal imaging (i) at the time of imaging (ii) at any time. Abbreviations: AMD = age-related macular degeneration, DR = diabetic retinopathy, MA = microaneurysm. [file EMS155920-supplement-sFigure_1.png]

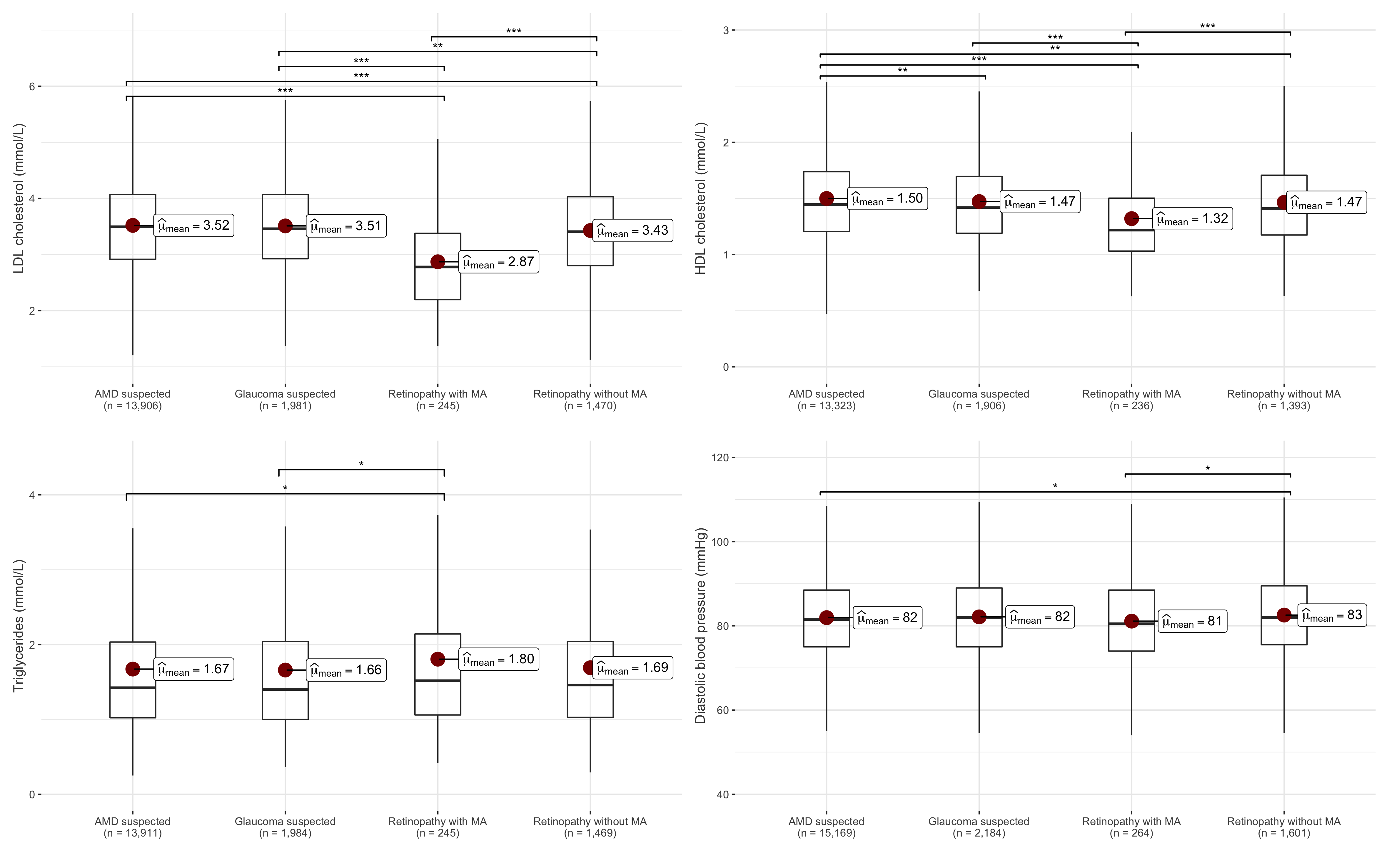

Supplement: sFigure 2 — Comparisons of baseline measurements for LDL and HDL cholesterol, triglycerides and diastolic blood pressure between participants who were identified from retinal imaging to have suspected age-related macular degeneration, glaucoma, diabetic retinopathy or other vascular disease. Statistically significant pairwise comparisons (Student’s t-test) are indicated by asterisks: p<0.05 (*), p<0.01 (**), p<0.001(***). Abbreviations: LDL = low-density lipoprotein cholesterol; HDL = high-density lipoprotein cholesterol. [file EMS155920-supplement-sFigure_2.png]
