# Supplementary material for: UK Biobank retinal imaging grading: methodology, baseline characteristics and findings for common ocular diseases
Source: Eye (Lond). Author manuscript; Available in PMC 2023 Jul 12. (PMC10333328; doi:10.1038/s41433-022-02298-7)
Supplement: Supplementary File 1 [file EMS155920-supplement-Supplementary_File_1.docx]

UK BIOBANK - Grading Protocol

A grading protocol was developed as a reference document for graders involved in UK Biobank (UKBB) to assist with the grading of retinal images and OCT scans.

Contents

[Image quality definitions for colour fundus photographs 3](#_Toc110844484)

[Good Quality 3](#_Toc110844485)

[Fair Quality 3](#_Toc110844486)

[Poor Quality 4](#_Toc110844487)

[Cannot Grade Quality 4](#_Toc110844488)

[Image quality definitions for OCT scans 5](#_Toc110844489)

[Good quality 5](#_Toc110844490)

[Fair quality 6](#_Toc110844491)

[Poor quality 6](#_Toc110844492)

[Cannot Grade 6](#_Toc110844493)

[Grading definitions 7](#_Toc110844494)

[Determining presence or absence of ocular abnormality features 7](#_Toc110844495)

## Image quality definitions for colour fundus photographs

Image quality was defined as good, fair, poor and cannot grade. These definitions were developed and revised by Consultant Ophthalmologists, Reading Centre Directors, and senior graders from NetwORC UK Reading Centres. The definitions as explained in the protocol are outlined below:

### Good Quality


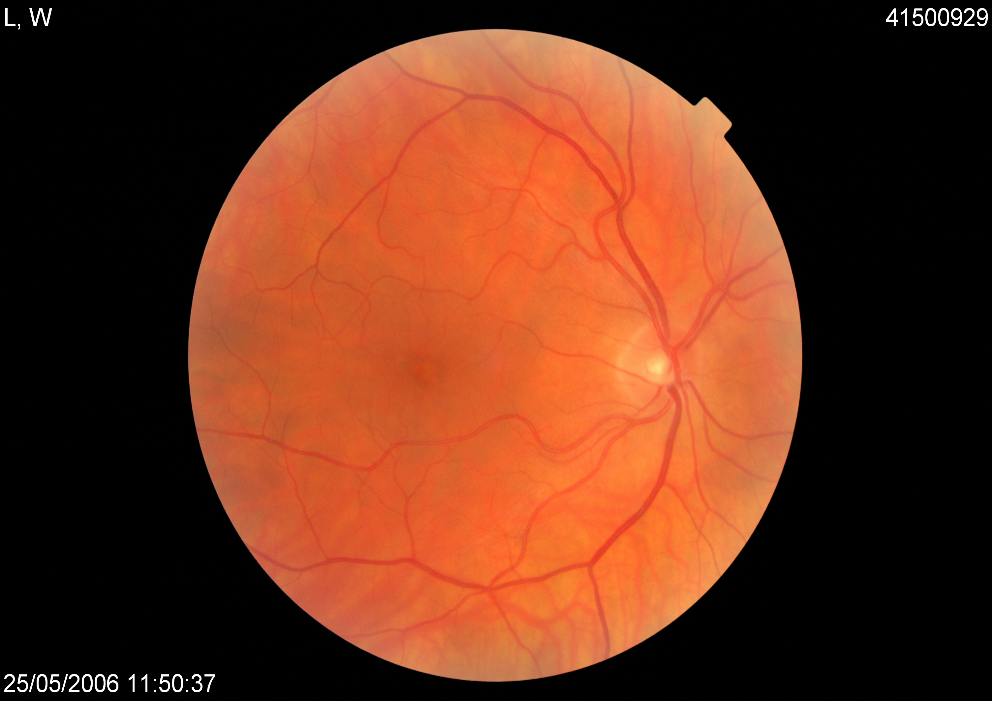
Good quality colour fundus photographs should have crisp focus, at least in the central and inner macular grids. Retinal details should be sharply defined and have crisp boundaries. It should be possible to see the ends of the larger vessels approaching the Foveal Avascular Zone (FAZ) in appropriate fields, if they are not obscured by pathology. It should also be possible to grade microaneurysms (approximately 63μm in size) in the macular region confidently if they are present.

Figure 1:Example of good quality colour fundus photograph

### Fair Quality

The colour fundus photograph is less well focused, with more difficultly to determine exactly where the ends of the larger vessels approaching the FAZ terminate. Retinal details are slightly indistinct but subtle lesions, such as small retinal haemorrhages, can still be graded. It is more difficult to confidently grade haemorrhages or exudates <125μm in size in the macular region.


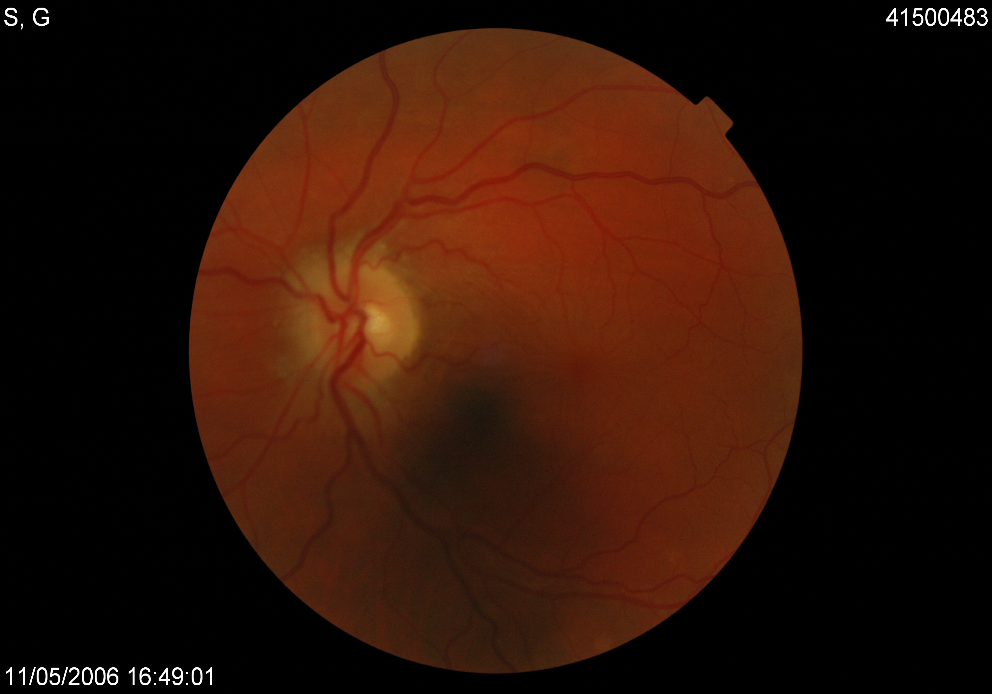


###

Figure 2: Example of fair quality colour fundus photograph

### Poor Quality

Poor quality indicates the colour fundus photograph is not well focused, but it is possible to identify some features for grading, such as new vessels on the disc or macular oedema. Clarity will be decreased so that subtle lesions might be missed but is sufficient for assessment of larger retinal haemorrhages and retinal vessels. Effectively major disease characteristics are gradable**.**

Figure 3: Example of poor quality colour fundus photograph


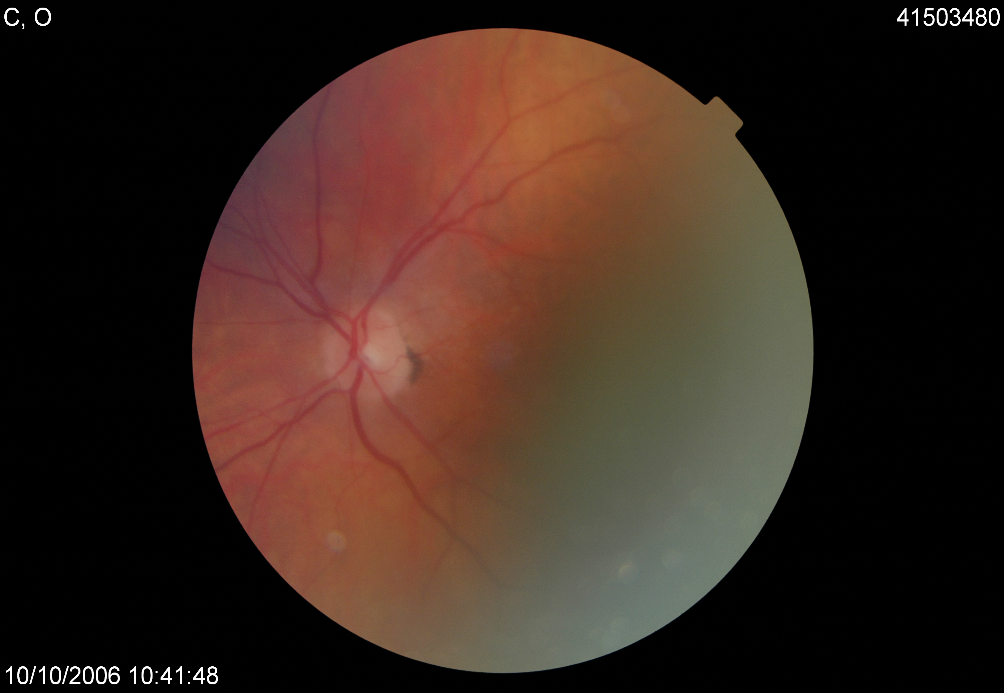


###

### Cannot Grade Quality

Cannot grade should be selected when the retinal vessels cannot be seen and less than 25% of the image is of sufficient quality to grade any lesion confidently. In this case graders should use other imaging modalities to complete grading.


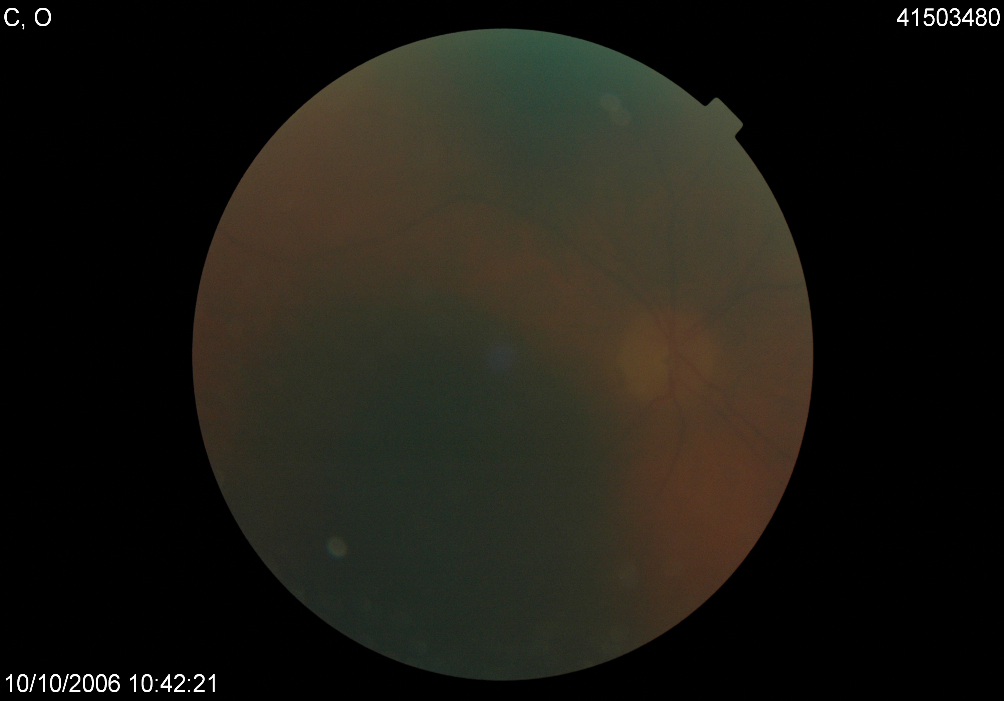


Figure 4: Example of cannot grade colour fundus photograph

## Image quality definitions for OCT scans

Quality assessments on the OCT scans should be made irrespective of the pathology present. If pathology is disrupting the scan extensively, graders should look at the edge of the scans or scans distant to pathology in order to judge the OCT image quality. The grader should assess the overall quality of all images supplied.

### Good quality

Excellent or good discrimination of retinal layers throughout the majority of the OCT scans supplied, especially in sub foveal region.


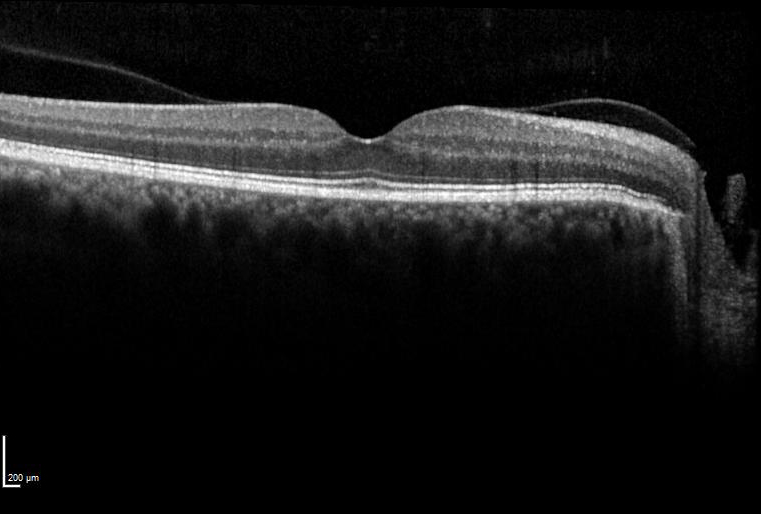


Figure 5: Example of good quality image

Figure 6:Example of good quality OCT scan

### Fair quality

Discrimination of retinal layers may be less distinct than ‘Good’ but still of suitable quality for acquisition of reliable data. This should be the case throughout majority of the OCT scans supplied especially in sub fovea region.


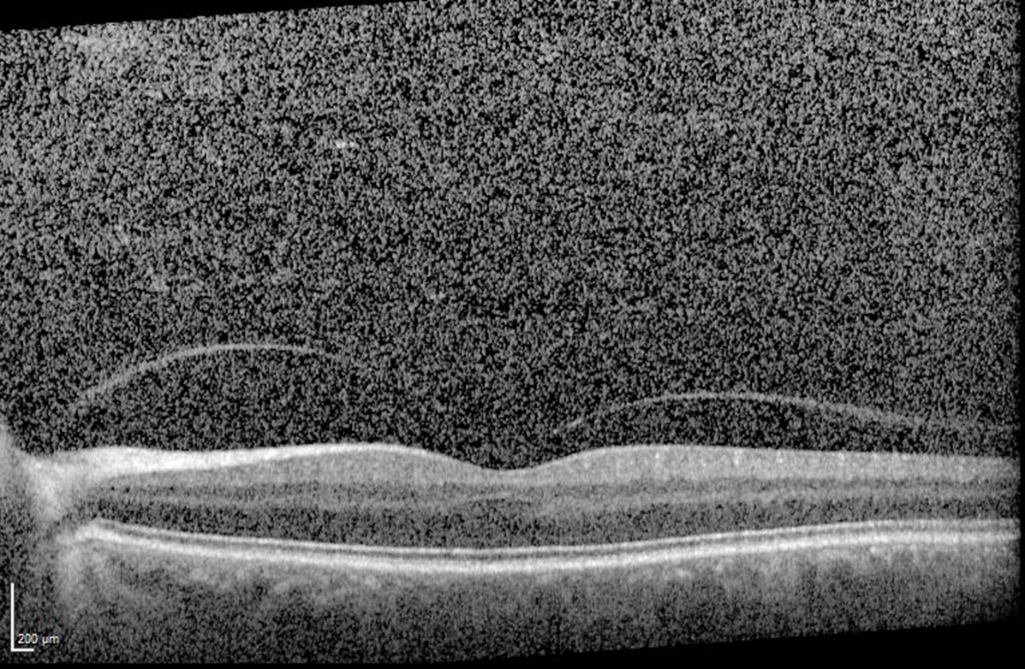


Figure 6: Example of fair quality OCT scan

## Poor quality

Discrimination of retinal layers is not distinct. Some information may still be discerned but no reliable measurements could be taken. This should be the case throughout majority of the OCT scans supplied especially in sub fovea region.


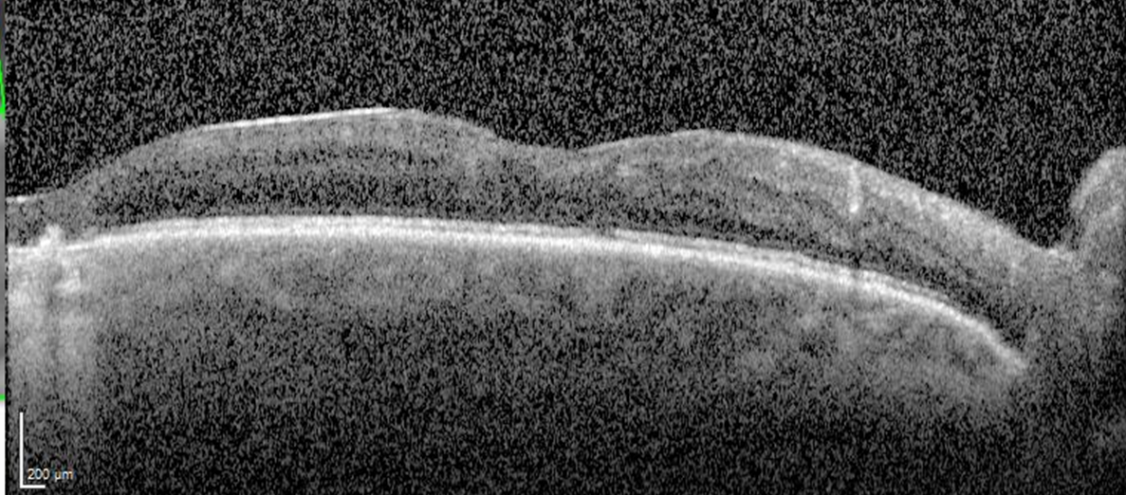


Figure 7: Example of poor quality OCT scan

### Cannot Grade

Discrimination of retinal layers is not of suitable quality for acquisition of reliable data. This should be the case throughout the majority of the OCT scans supplied especially in sub foveal region.


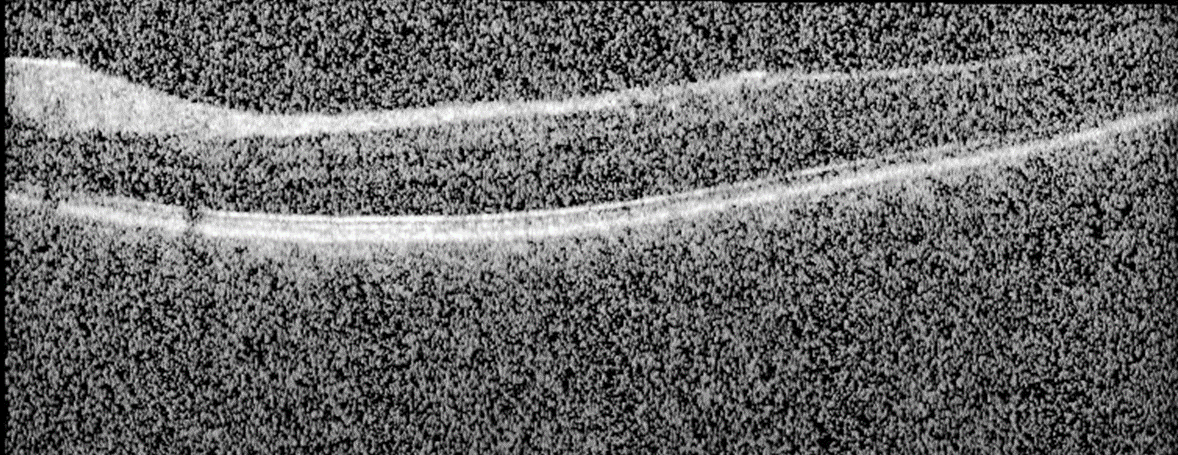


## Grading definitions

Figure 8: Example of cannot grade OCT scan

Study specific grading forms were designed to capture a variety of retinal features. Based on the grading form questions, definitions were developed and revised by Consultant Ophthalmologists, Reading Centre Directors, and senior graders from NetwORC UK Reading Centres. These definitions in addition to reference images were supplied to assist graders during the grading process. Refer to the UKBB data dictionary for further information.

## Determining presence or absence of ocular abnormality features

The presence or absence of ocular abnormalities was based on the following confidence levels:

- ‘Present’ - the grader is at least 90% certain that the abnormality is present in the area under consideration.
- ‘Absent’ - the grader is less than 90% confident that the abnormality under consideration is visible in the area being graded and that at least 25% of this area can be seen sufficiently well to be sure it is free of the abnormality. If the grader identifies an abnormality that cannot be confidently categorised, they should seek a second opinion or select adjudication.
- ‘Cannot Grade’ - Less than 25% of the area under consideration can be graded and this area is free from abnormalities.
