# Supplementary material for: Correction: UK Biobank retinal imaging grading: methodology, baseline characteristics and findings for common ocular diseases
Source: Eye (Lond). 2023 Jan 5;37(10):2163. doi: 10.1038/s41433-022-02377-9 (PMC10333216; doi:10.1038/s41433-022-02377-9)
Supplement: Supplementary file 1 — Supplementary Table [file 41433_2022_2377_MOESM1_ESM.docx]

**sTable 1.** Data dictionary for available variables in the retinal gradings dataset. Variables relating specifically to either colour fundus photographs or optical coherence tomography scans are prefixed with ‘COL_’ and ‘OCT_’ respectively. Those without a prefix are multimodal. Column ‘PersonEye’ indicates whether variables are recorded at either a person or eye level.

| **Field** | **Notes** | **ValueType** | **PersonEye** |
| --- | --- | --- | --- |
| VisitDate | The date of the visit; DD/MM/YYYY | Date | person |
| CupDiscRatioComparison | Absolute cup disc ratio comparison; 99 if CDR is missing for one or both eyes | Continuous | person |
| COL_Available | Are colour images available? Yes, No | Categorical single | eye |
| OCT_Available | Are optical coherence tomography (OCT) scans available? Yes, No | Categorical single | eye |
| COL_Quality | Colour image quality – Good, Fair, Poor or Cannot grade [CG]; NULL if COL_Available is No | Categorical single | eye |
| OCT_Quality | OCT scan quality – Good, Fair, Poor or Cannot grade [CG]; NULL if OCT_Available is No | Categorical single | eye |
| AbnormalitiesPresent | Are any abnormalities present? Yes, No; NULL only if all modalities are unavailable or ungradable | Categorical single | eye |
| AMD | Is there evidence of age-related macular degeneration (AMD)? Yes, No; NULL if AbnormalitiesPresent is No or NULL | Categorical single | eye |
| COL_IfAMD | On colour, is AMD: Exudative [Exud], Geographic atrophy [GA], Hyperpigmentation [Pigment], Drusen; NULL if AbnormalitiesPresent is No or NULL, colour images are unavailable or ungradable, or if AMD is not visible on colour images | Compound | eye |
| COL_DrusenInsideGrid | Are there drusen inside the grid? Yes, No; NULL if AbnormalitiesPresent is No or NULL, AMD is No or COL_IfAMD does not contain Drusen | Categorical single | eye |
| COL_SevereDrusen | Most severe drusen type in grid: <63µm [LessThan63], 63-125µm [SixtyThreeTo125], >125µm [GreaterThan125], Cannot grade [CG]; NULL if COL_DrusenInsideGrid is No or NULL | Categorical single | eye |
| COL_DrusenOutsideGrid | Are there drusen outside the grid? Yes, No; NULL if AbnormalitiesPresent is No, AMD is No or COL_IfAMD does not contain Drusen | Categorical single | eye |
| COL_ReticularDrusen | Reticular pseudodrusen? Yes, No, CG; NULL if AbnormalitiesPresent is No, AMD is No or COL_IfAMD does not contain Drusen | Categorical single | eye |
| OCT_DrusenPresent | On OCT, are drusen present? Yes, No; NULL if AbnormalitiesPresent is No, AMD is No or OCT images are unavailable or ungradable | Categorical single | eye |
| OCT_SubretDrusen | On OCT, are there subretinal drusenoid deposits? Yes, No; NULL if AbnormalitiesPresent is No, AMD is No or OCT images are unavailable or ungradable | Categorical single | eye |
| OCT_FocalAtrophy | On OCT, is there focal atrophy? Yes, No; NULL if AbnormalitiesPresent is No, AMD is No or OCT images are unavailable or ungradable | Categorical single | eye |
| OCT_CentralSubfieldThickness | Central subfield thickness; Integer obtained from automated OCT machine thickness map; NULL if OCT images are unavailable or ungradable | Integer | eye |
| COL_RetinalHaemorrhage | Is there evidence of retinal haemorrhage or microaneurysm? Yes, No; NULL if AbnormalitiesPresent is No or NULL if COL images are unavailable or ungradable | Categorical single | eye |
| COL_SignsOfDR | If COL_RetinalHaemorrhage is Yes, is it accompanied by characteristic signs of diabetic retinopathy? Yes, No; NULL if COL_RetinalHaemorrhage is No or NULL | Categorical single | eye |
| COL_DRFeatures | If COL_SignsOfDR is Yes, select all the characteristics that apply: Microaneurysm/haemorrhage [HMA], Venous loop [VL], Exudate [Ex], Cotton wool spots [CWS], Venous beading [VB], Venous reduplication [VR], Multiple blot haemorrhages [MBH], intra-retinal microvascular abnormalities [IRMA], Fibrous proliferation [FP], Laser scars [LS], New vessels on disc [NVD], New vessels elsewhere [NVE], Pre-retinal or vitreous haemorrhage [PVH], Pre-retinal fibrosis [PF], tractional retinal detachment [TRD]; NULL if COL_SignsOfDR is No or NULL | Compound | eye |
| COL_VisibleLaserTreatment | Any visible laser treatment? Yes, No; NULL if AbnormalitiesPresent is No or NULL; or COL images are unavailable or ungradable | Categorical single | eye |
| OCT_HyporeflectiveIntraretinalSpaces | On OCT, are there hyporeflective intraretinal spaces? Yes, No; NULL if AbnormalitiesPresent is No or NULL; or OCT images are unavailable or ungradable | Categorical single | eye |
| COL_CupDiscRatio | Cup disc ratio (CDR); Floating point measurement; NULL if CDR not gradable or COL images are unavailable | Continuous | eye |
| COL_HaemorrhageOnDisc | Haemorrhage on disc? Yes, No, Cannot grade [CG]; NULL if AbnormalitiesPresent is No or NULL | Categorical single | eye |
| COL_IfOpticDiscSuspicious | If suspicious: Notch, Inferior rim thinning [IRT], Notch and Inferior rim thinning [Both]; NULL if COL_CupDiscRatio is ≥0.7 or COL_HaemorrhageOnDisc is Yes | Categorical single | eye |
| Occlusion | Occlusion? Yes, No; NULL if AbnormalitiesPresent is No or NULL | Categorical single | eye |
| IfOcclusion | If Occlusion is Yes: Central retinal vein occlusion [CRVO], Branch retinal vein occlusion [BRVO], Central retinal artery occlusion [CRAO], Branch retinal artery occlusion [BRAO]; NULL if Occlusion is No or NULL | Categorical single | eye |
| EvidenceOfERM | Is there evidence of Epi-retinal membrane [ERM]? Yes, No; NULL if AbnormalitiesPresent is No or NULL | Categorical single | eye |
| IfERMStructuralChanges | Are there any retinal structural changes associated with the ERM? Yes, No; NULL if EvidenceOfERM is No or NULL | Categorical single | eye |
| OCT_EvidenceOfVMA | Is there evidence of vitreomacular adhesion [VMA]? Yes, No; NULL if AbnormalitiesPresent is No or NULL; or OCT images are unavailable or ungradable | Categorical single | eye |
| OCT_IfVMACategories | VMA categories: WithTraction, WithoutTraction; NULL if OCT_EvidenceOfVMA is No or NULL | Categorical single | eye |
| Other | Are other features present? Yes, No; NULL if AbnormalitiesPresent is No or NULL | Categorical single | eye |
| IfOther | If Other is Yes, select all that apply: Asteroid hyalosis [AH], Central serous chorio-retinopathy [CSCR], Macular hole (any stage) [MH], Macular telangiectasia [MacTel], Media opacity [MO], Myopic degeneration [MD], Naevus/Melanoma [Naev], Retinal detachment [RD], Schisis/foveal cystic changes [SFCS], Other; NULL if Other is No or NULL | Compound | eye |
| OtherComment | If Other was selected, please comment: Free-text; NULL if Other is No or NULL; NULL if IfOther does not contain Other | Compound | eye |
| IfOtherMerged | IfOther and OtherComment combined | Compound | eye |

**sTable 2.** Clinical codes used to identify diagnosed ocular (age-related macular degeneration, diabetic retinopathy and glaucoma) and systemic disease (diabetes, hypertension) in the UK Biobank from self-reported (UK Biobank data coding 6) and linked primary care (Read 2 and Read 3), hospital episodes statistics and death register (ICD-9 and ICD-10) data.

| **Disease** | **Code** | **Code type** | **Description** |
| --- | --- | --- | --- |
| Age-related macular degeneration | 1528 | Data coding 6 | Macular degeneration |
| Age-related macular degeneration | H353 | ICD-10 | Degeneration of macula and posterior pole |
| Age-related macular degeneration | 3625 | ICD-9 | DEGENERATION OF MACULA AND POSTERIOR POLE |
| Age-related macular degeneration | 2BBH. | Read 2 | Retinal drusen |
| Age-related macular degeneration | F425. | Read 2 | Degeneration of macula and posterior pole |
| Age-related macular degeneration | F4250 | Read 2 | Unspecified senile macular degeneration |
| Age-related macular degeneration | F4251 | Read 2 | Dry senile macular degeneration |
| Age-related macular degeneration | F4252 | Read 2 | Wet senile macular degeneration |
| Age-related macular degeneration | F4257 | Read 2 | Drusen |
| Age-related macular degeneration | F425z | Read 2 | Degeneration of macula or posterior pole NOS |
| Age-related macular degeneration | F4250 | Read 3 | Unspecified senile macular degeneration |
| Age-related macular degeneration | F4251 | Read 3 | Atrophic age-related macular degeneration |
| Age-related macular degeneration | F4252 | Read 3 | Subretinal neovascularisation of macula |
| Age-related macular degeneration | F4257 | Read 3 | Drusen |
| Age-related macular degeneration | F425z | Read 3 | Degeneration of macula or posterior pole NOS |
| Age-related macular degeneration | X75mo | Read 3 | Retinal drusen |
| Age-related macular degeneration | XE15x | Read 3 | Degeneration of macula and posterior pole |
| Age-related macular degeneration | XE18j | Read 3 | Age-related macular degeneration |
| Age-related macular degeneration | X75mp | Read 3 | Macular drusen |
| Age-related macular degeneration | XaF41 | Read 3 | Drusen plus pigment change stage macular degeneration |
| Age-related macular degeneration | Xa9BN | Read 3 | Macular degeneration |
| Diabetes | 1220 | Data coding 6 | diabetes |
| Diabetes | 1221 | Data coding 6 | gestational diabetes |
| Diabetes | 1222 | Data coding 6 | type 1 diabetes |
| Diabetes | 1223 | Data coding 6 | type 2 diabetes |
| Diabetes | E13 | ICD-10 | Other specified diabetes mellitus |
| Diabetes | E14 | ICD-10 | Unspecified diabetes mellitus |
| Diabetes | G590 | ICD-10 | Diabetic mononeuropathy |
| Diabetes | G632 | ICD-10 | Diabetic polyneuropathy |
| Diabetes | H280 | ICD-10 | Diabetic cataract |
| Diabetes | H360 | ICD-10 | Diabetic retinopathy |
| Diabetes | M142 | ICD-10 | Diabetic arthropathy |
| Diabetes | N083 | ICD-10 | Glomerular disorders in diabetes mellitus |
| Diabetes | O240 | ICD-10 | Diabetes mellitus in pregnancy: Pre-existing diabetes mellitus, insulin-dependent |
| Diabetes | O241 | ICD-10 | Diabetes mellitus in pregnancy: Pre-existing diabetes mellitus, non-insulin-dependent |
| Diabetes | O243 | ICD-10 | Diabetes mellitus in pregnancy: Pre-existing diabetes mellitus, unspecified |
| Diabetes | E130 | ICD-10 | Other specified diabetes mellitus With coma |
| Diabetes | E131 | ICD-10 | Other specified diabetes mellitus With ketoacidosis |
| Diabetes | E132 | ICD-10 | Other specified diabetes mellitus With renal complications |
| Diabetes | E133 | ICD-10 | Other specified diabetes mellitus With ophthalmic complications |
| Diabetes | E134 | ICD-10 | Other specified diabetes mellitus With neurological complications |
| Diabetes | E135 | ICD-10 | Other specified diabetes mellitus With peripheral circulatory complications |
| Diabetes | E136 | ICD-10 | Other specified diabetes mellitus With other specified complications |
| Diabetes | E137 | ICD-10 | Other specified diabetes mellitus With multiple complications |
| Diabetes | E138 | ICD-10 | Other specified diabetes mellitus With unspecified complications |
| Diabetes | E139 | ICD-10 | Other specified diabetes mellitus Without complications |
| Diabetes | E140 | ICD-10 | Unspecified diabetes mellitus With coma |
| Diabetes | E141 | ICD-10 | Unspecified diabetes mellitus With ketoacidosis |
| Diabetes | E142 | ICD-10 | Unspecified diabetes mellitus With renal complications |
| Diabetes | E143 | ICD-10 | Unspecified diabetes mellitus With ophthalmic complications |
| Diabetes | E144 | ICD-10 | Unspecified diabetes mellitus With neurological complications |
| Diabetes | E145 | ICD-10 | Unspecified diabetes mellitus With peripheral circulatory complications |
| Diabetes | E146 | ICD-10 | Unspecified diabetes mellitus With other specified complications |
| Diabetes | E147 | ICD-10 | Unspecified diabetes mellitus With multiple complications |
| Diabetes | E148 | ICD-10 | Unspecified diabetes mellitus With unspecified complications |
| Diabetes | E149 | ICD-10 | Unspecified diabetes mellitus Without complications |
| Diabetes | E10 | ICD-10 | Insulin-dependent diabetes mellitus |
| Diabetes | E100 | ICD-10 | Type 1 diabetes mellitus With coma |
| Diabetes | E101 | ICD-10 | Type 1 diabetes mellitus With ketoacidosis |
| Diabetes | E102 | ICD-10 | Type 1 diabetes mellitus With renal complications |
| Diabetes | E103 | ICD-10 | Type 1 diabetes mellitus With ophthalmic complications |
| Diabetes | E104 | ICD-10 | Type 1 diabetes mellitus With neurological complications |
| Diabetes | E105 | ICD-10 | Type 1 diabetes mellitus With peripheral circulatory complications |
| Diabetes | E106 | ICD-10 | Type 1 diabetes mellitus With other specified complications |
| Diabetes | E107 | ICD-10 | Type 1 diabetes mellitus With multiple complications |
| Diabetes | E108 | ICD-10 | Type 1 diabetes mellitus With unspecified complications |
| Diabetes | E109 | ICD-10 | Type 1 diabetes mellitus Without complications |
| Diabetes | E11 | ICD-10 | Non-insulin-dependent diabetes mellitus |
| Diabetes | E110 | ICD-10 | Type 2 diabetes mellitus With coma |
| Diabetes | E111 | ICD-10 | Type 2 diabetes mellitus With ketoacidosis |
| Diabetes | E112 | ICD-10 | Type 2 diabetes mellitus With renal complications |
| Diabetes | E113 | ICD-10 | Type 2 diabetes mellitus With ophthalmic complications |
| Diabetes | E114 | ICD-10 | Type 2 diabetes mellitus With neurological complications |
| Diabetes | E115 | ICD-10 | Type 2 diabetes mellitus With peripheral circulatory complications |
| Diabetes | E116 | ICD-10 | Type 2 diabetes mellitus With other specified complications |
| Diabetes | E117 | ICD-10 | Type 2 diabetes mellitus With multiple complications |
| Diabetes | E118 | ICD-10 | Type 2 diabetes mellitus With unspecified complications |
| Diabetes | E119 | ICD-10 | Type 2 diabetes mellitus Without complications |
| Diabetes | E12 | ICD-10 | Malnutrition-related diabetes mellitus |
| Diabetes | O242 | ICD-10 | Diabetes mellitus in pregnancy: Pre-existing malnutrition-related diabetes mellitus |
| Diabetes | E120 | ICD-10 | Malnutrition-related diabetes mellitus With coma |
| Diabetes | E121 | ICD-10 | Malnutrition-related diabetes mellitus With ketoacidosis |
| Diabetes | E122 | ICD-10 | Malnutrition-related diabetes mellitus With renal complications |
| Diabetes | E123 | ICD-10 | Malnutrition-related diabetes mellitus With ophthalmic complications |
| Diabetes | E124 | ICD-10 | Malnutrition-related diabetes mellitus With neurological complications |
| Diabetes | E125 | ICD-10 | Malnutrition-related diabetes mellitus With peripheral circulatory complications |
| Diabetes | E126 | ICD-10 | Malnutrition-related diabetes mellitus With other specified complications |
| Diabetes | E127 | ICD-10 | Malnutrition-related diabetes mellitus With multiple complications |
| Diabetes | E128 | ICD-10 | Malnutrition-related diabetes mellitus With unspecified complications |
| Diabetes | E129 | ICD-10 | Malnutrition-related diabetes mellitus Without complications |
| Diabetes | 6480 | ICD-9 | DIABETES MELLITUS |
| Diabetes | C108. | Read 2 | Type 1 diabetes mellitus |
| Diabetes | C1080 | Read 2 | Type I diabetes mellitus with renal complications |
| Diabetes | C1082 | Read 2 | Type I diabetes mellitus with neurological complications |
| Diabetes | C1084 | Read 2 | Unstable type I diabetes mellitus |
| Diabetes | C1085 | Read 2 | Type I diabetes mellitus with ulcer |
| Diabetes | C1087 | Read 2 | Type I diabetes mellitus with retinopathy |
| Diabetes | C1088 | Read 2 | Type I diabetes mellitus - poor control |
| Diabetes | C1089 | Read 2 | Type I diabetes mellitus maturity onset |
| Diabetes | C108A | Read 2 | Type I diabetes mellitus without complication |
| Diabetes | C108D | Read 2 | Type I diabetes mellitus with nephropathy |
| Diabetes | C108E | Read 2 | Type I diabetes mellitus with hypoglycaemic coma |
| Diabetes | C108F | Read 2 | Type I diabetes mellitus with diabetic cataract |
| Diabetes | C108H | Read 2 | Type I diabetes mellitus with arthropathy |
| Diabetes | C108J | Read 2 | Type I diabetes mellitus with neuropathic arthropathy |
| Diabetes | C10E. | Read 2 | Type 1 diabetes mellitus |
| Diabetes | C10E0 | Read 2 | Type 1 diabetes mellitus with renal complications |
| Diabetes | C10E1 | Read 2 | Type 1 diabetes mellitus with ophthalmic complications |
| Diabetes | C10E2 | Read 2 | Type 1 diabetes mellitus with neurological complications |
| Diabetes | C10E3 | Read 2 | Type 1 diabetes mellitus with multiple complications |
| Diabetes | C10E4 | Read 2 | Unstable type 1 diabetes mellitus |
| Diabetes | C10E5 | Read 2 | Type 1 diabetes mellitus with ulcer |
| Diabetes | C10E6 | Read 2 | Type 1 diabetes mellitus with gangrene |
| Diabetes | C10E7 | Read 2 | Type 1 diabetes mellitus with retinopathy |
| Diabetes | C10E8 | Read 2 | Type 1 diabetes mellitus - poor control |
| Diabetes | C10E9 | Read 2 | Type 1 diabetes mellitus maturity onset |
| Diabetes | C10EA | Read 2 | Type 1 diabetes mellitus without complication |
| Diabetes | C10EB | Read 2 | Type 1 diabetes mellitus with mononeuropathy |
| Diabetes | C10EC | Read 2 | Type 1 diabetes mellitus with polyneuropathy |
| Diabetes | C10ED | Read 2 | Type 1 diabetes mellitus with nephropathy |
| Diabetes | C10EE | Read 2 | Type 1 diabetes mellitus with hypoglycaemic coma |
| Diabetes | C10EF | Read 2 | Type 1 diabetes mellitus with diabetic cataract |
| Diabetes | C10EG | Read 2 | Type 1 diabetes mellitus with peripheral angiopathy |
| Diabetes | C10EH | Read 2 | Type 1 diabetes mellitus with arthropathy |
| Diabetes | C10EJ | Read 2 | Type 1 diabetes mellitus with neuropathic arthropathy |
| Diabetes | C10EK | Read 2 | Type 1 diabetes mellitus with persistent proteinuria |
| Diabetes | C10EL | Read 2 | Type 1 diabetes mellitus with persistent microalbuminuria |
| Diabetes | C10EM | Read 2 | Type 1 diabetes mellitus with ketoacidosis |
| Diabetes | C10EN | Read 2 | Type 1 diabetes mellitus with ketoacidotic coma |
| Diabetes | C10EP | Read 2 | Type 1 diabetes mellitus with exudative maculopathy |
| Diabetes | C10EQ | Read 2 | Type 1 diabetes mellitus with gastroparesis |
| Diabetes | C109. | Read 2 | Type 2 diabetes mellitus |
| Diabetes | C1090 | Read 2 | Type II diabetes mellitus with renal complications |
| Diabetes | C1091 | Read 2 | Type II diabetes mellitus with ophthalmic complications |
| Diabetes | C1092 | Read 2 | Type II diabetes mellitus with neurological complications |
| Diabetes | C1094 | Read 2 | Type II diabetes mellitus with ulcer |
| Diabetes | C1095 | Read 2 | Type II diabetes mellitus with gangrene |
| Diabetes | C1096 | Read 2 | Type II diabetes mellitus with retinopathy |
| Diabetes | C1097 | Read 2 | Type II diabetes mellitus - poor control |
| Diabetes | C109A | Read 2 | Type II diabetes mellitus with mononeuropathy |
| Diabetes | C109B | Read 2 | Type II diabetes mellitus with polyneuropathy |
| Diabetes | C109C | Read 2 | Type II diabetes mellitus with nephropathy |
| Diabetes | C109D | Read 2 | Type II diabetes mellitus with hypoglycaemic coma |
| Diabetes | C109E | Read 2 | Type II diabetes mellitus with diabetic cataract |
| Diabetes | C109F | Read 2 | Type II diabetes mellitus with peripheral angiopathy |
| Diabetes | C109G | Read 2 | Type II diabetes mellitus with arthropathy |
| Diabetes | C109H | Read 2 | Type II diabetes mellitus with neuropathic arthropathy |
| Diabetes | C109J | Read 2 | Insulin treated Type 2 diabetes mellitus |
| Diabetes | C109K | Read 2 | Hyperosmolar non-ketotic state in type 2 diabetes mellitus |
| Diabetes | C10F. | Read 2 | Type 2 diabetes mellitus |
| Diabetes | C10F0 | Read 2 | Type 2 diabetes mellitus with renal complications |
| Diabetes | C10F1 | Read 2 | Type 2 diabetes mellitus with ophthalmic complications |
| Diabetes | C10F2 | Read 2 | Type 2 diabetes mellitus with neurological complications |
| Diabetes | C10F3 | Read 2 | Type 2 diabetes mellitus with multiple complications |
| Diabetes | C10F4 | Read 2 | Type 2 diabetes mellitus with ulcer |
| Diabetes | C10F5 | Read 2 | Type 2 diabetes mellitus with gangrene |
| Diabetes | C10F6 | Read 2 | Type 2 diabetes mellitus with retinopathy |
| Diabetes | C10F7 | Read 2 | Type 2 diabetes mellitus - poor control |
| Diabetes | C10F9 | Read 2 | Type 2 diabetes mellitus without complication |
| Diabetes | C10FA | Read 2 | Type 2 diabetes mellitus with mononeuropathy |
| Diabetes | C10FB | Read 2 | Type 2 diabetes mellitus with polyneuropathy |
| Diabetes | C10FC | Read 2 | Type 2 diabetes mellitus with nephropathy |
| Diabetes | C10FD | Read 2 | Type 2 diabetes mellitus with hypoglycaemic coma |
| Diabetes | C10FE | Read 2 | Type 2 diabetes mellitus with diabetic cataract |
| Diabetes | C10FF | Read 2 | Type 2 diabetes mellitus with peripheral angiopathy |
| Diabetes | C10FG | Read 2 | Type 2 diabetes mellitus with arthropathy |
| Diabetes | C10FH | Read 2 | Type 2 diabetes mellitus with neuropathic arthropathy |
| Diabetes | C10FJ | Read 2 | Insulin treated Type 2 diabetes mellitus |
| Diabetes | C10FK | Read 2 | Hyperosmolar non-ketotic state in type 2 diabetes mellitus |
| Diabetes | C10FL | Read 2 | Type 2 diabetes mellitus with persistent proteinuria |
| Diabetes | C10FM | Read 2 | Type 2 diabetes mellitus with persistent microalbuminuria |
| Diabetes | C10FN | Read 2 | Type 2 diabetes mellitus with ketoacidosis |
| Diabetes | C10FP | Read 2 | Type 2 diabetes mellitus with ketoacidotic coma |
| Diabetes | C10FQ | Read 2 | Type 2 diabetes mellitus with exudative maculopathy |
| Diabetes | C10FR | Read 2 | Type 2 diabetes mellitus with gastroparesis |
| Diabetes | X40J4 | Read 3 | Type I diabetes mellitus |
| Diabetes | C1080 | Read 3 | Type I diabetes mellitus with renal complications |
| Diabetes | C1082 | Read 3 | Type I diabetes mellitus with neurological complications |
| Diabetes | Xa4g7 | Read 3 | Unstable type I diabetes mellitus |
| Diabetes | C1085 | Read 3 | Type I diabetes mellitus with ulcer |
| Diabetes | C1087 | Read 3 | Type I diabetes mellitus with retinopathy |
| Diabetes | C1088 | Read 3 | Type I diabetes mellitus - poor control |
| Diabetes | C1089 | Read 3 | Type I diabetes mellitus maturity onset |
| Diabetes | XaELP | Read 3 | Type I diabetes mellitus without complication |
| Diabetes | XaF04 | Read 3 | Type I diabetes mellitus with nephropathy |
| Diabetes | XaFWG | Read 3 | Type I diabetes mellitus with hypoglycaemic coma |
| Diabetes | XaFm8 | Read 3 | Type I diabetes mellitus with diabetic cataract |
| Diabetes | XaFmL | Read 3 | Type I diabetes mellitus with arthropathy |
| Diabetes | XaFmM | Read 3 | Type I diabetes mellitus with neuropathic arthropathy |
| Diabetes | C1081 | Read 3 | Type I diabetes mellitus with ophthalmic complications |
| Diabetes | C1083 | Read 3 | Type I diabetes mellitus with multiple complications |
| Diabetes | C1086 | Read 3 | Type I diabetes mellitus with gangrene |
| Diabetes | XaEnn | Read 3 | Type I diabetes mellitus with mononeuropathy |
| Diabetes | XaEno | Read 3 | Type I diabetes mellitus with polyneuropathy |
| Diabetes | XaFmK | Read 3 | Type I diabetes mellitus with peripheral angiopathy |
| Diabetes | XaIzM | Read 3 | Type 1 diabetes mellitus with persistent proteinuria |
| Diabetes | XaIzN | Read 3 | Type 1 diabetes mellitus with persistent microalbuminuria |
| Diabetes | C1010 | Read 3 | Type 1 diabetes mellitus with ketoacidosis |
| Diabetes | C1030 | Read 3 | Type 1 diabetes mellitus with ketoacidotic coma |
| Diabetes | XaJSr | Read 3 | Type 1 diabetes mellitus with exudative maculopathy |
| Diabetes | XaKyW | Read 3 | Type 1 diabetes mellitus with gastroparesis |
| Diabetes | X40J5 | Read 3 | Type II diabetes mellitus |
| Diabetes | C1090 | Read 3 | Type II diabetes mellitus with renal complications |
| Diabetes | C1091 | Read 3 | Type II diabetes mellitus with ophthalmic complications |
| Diabetes | C1092 | Read 3 | Type II diabetes mellitus with neurological complications |
| Diabetes | C1094 | Read 3 | Type II diabetes mellitus with ulcer |
| Diabetes | C1095 | Read 3 | Type II diabetes mellitus with gangrene |
| Diabetes | C1096 | Read 3 | Type II diabetes mellitus with retinopathy |
| Diabetes | C1097 | Read 3 | Type II diabetes mellitus - poor control |
| Diabetes | XaEnp | Read 3 | Type II diabetes mellitus with mononeuropathy |
| Diabetes | XaEnq | Read 3 | Type II diabetes mellitus with polyneuropathy |
| Diabetes | XaF05 | Read 3 | Type II diabetes mellitus with nephropathy |
| Diabetes | XaFWI | Read 3 | Type II diabetes mellitus with hypoglycaemic coma |
| Diabetes | XaFmA | Read 3 | Type II diabetes mellitus with diabetic cataract |
| Diabetes | XaFn7 | Read 3 | Type II diabetes mellitus with peripheral angiopathy |
| Diabetes | XaFn8 | Read 3 | Type II diabetes mellitus with arthropathy |
| Diabetes | XaFn9 | Read 3 | Type II diabetes mellitus with neuropathic arthropathy |
| Diabetes | X40J6 | Read 3 | Insulin treated Type 2 diabetes mellitus |
| Diabetes | XaIrf | Read 3 | Hyperosmolar non-ketotic state in type II diabetes mellitus |
| Diabetes | C1093 | Read 3 | Type II diabetes mellitus with multiple complications |
| Diabetes | XaELQ | Read 3 | Type II diabetes mellitus without complication |
| Diabetes | XaIzQ | Read 3 | Type II diabetes mellitus with persistent proteinuria |
| Diabetes | XaIzR | Read 3 | Type II diabetes mellitus with persistent microalbuminuria |
| Diabetes | C1011 | Read 3 | Type 2 diabetes mellitus with ketoacidosis |
| Diabetes | C1031 | Read 3 | Type II diabetes mellitus with ketoacidotic coma |
| Diabetes | XaJQp | Read 3 | Type II diabetes mellitus with exudative maculopathy |
| Diabetes | XaKyX | Read 3 | Type II diabetes mellitus with gastroparesis |
| Diabetic retinopathy | 1276 | Data coding 6 | diabetic eye disease |
| Diabetic retinopathy | E103 | ICD-10 | Type 1 diabetes mellitus With ophthalmic complications |
| Diabetic retinopathy | E113 | ICD-10 | Type 2 diabetes mellitus With ophthalmic complications |
| Diabetic retinopathy | E123 | ICD-10 | Malnutrition-related diabetes mellitus With ophthalmic complications |
| Diabetic retinopathy | E133 | ICD-10 | Other specified diabetes mellitus With ophthalmic complications |
| Diabetic retinopathy | E143 | ICD-10 | Unspecified diabetes mellitus With ophthalmic complications |
| Diabetic retinopathy | H360 | ICD-10 | Diabetic retinopathy |
| Diabetic retinopathy | 2504 | ICD-9 | DIABETES WITH OPHTHALMIC MANIFESTATIONS |
| Diabetic retinopathy | 3620A | ICD-9 | DIABETIC RETINOPATHY |
| Diabetic retinopathy | 2BBF. | Read 2 | Retinal abnormality - diabetes related |
| Diabetic retinopathy | C105. | Read 2 | Diabetes mellitus with ophthalmic manifestation |
| Diabetic retinopathy | C1050 | Read 2 | Diabetes mellitus, juvenile type, with ophthalmic manifestation |
| Diabetic retinopathy | C1051 | Read 2 | Diabetes mellitus, adult onset, with ophthalmic manifestation |
| Diabetic retinopathy | C105y | Read 2 | Other specified diabetes mellitus with ophthalmic complications |
| Diabetic retinopathy | C105z | Read 2 | Diabetes mellitus NOS with ophthalmic manifestation |
| Diabetic retinopathy | C1081 | Read 2 | Insulin-dependent diabetes mellitus with ophthalmic complications |
| Diabetic retinopathy | C1087 | Read 2 | Insulin dependent diabetes mellitus with retinopathy |
| Diabetic retinopathy | C1091 | Read 2 | Non-insulin-dependent diabetes mellitus with ophthalmic complications |
| Diabetic retinopathy | C1096 | Read 2 | Non-insulin-dependent diabetes mellitus with retinopathy |
| Diabetic retinopathy | C10A3 | Read 2 | Malnutrition-related diabetes mellitus with ophthalmic complications |
| Diabetic retinopathy | C10E1 | Read 2 | Type 1 diabetes mellitus with ophthalmic complications |
| Diabetic retinopathy | C10E7 | Read 2 | Type 1 diabetes mellitus with retinopathy |
| Diabetic retinopathy | C10F1 | Read 2 | Type 2 diabetes mellitus with ophthalmic complications |
| Diabetic retinopathy | C10F6 | Read 2 | Type 2 diabetes mellitus with retinopathy |
| Diabetic retinopathy | F420. | Read 2 | Diabetic retinopathy |
| Diabetic retinopathy | F420z | Read 2 | Diabetic retinopathy NOS |
| Diabetic retinopathy | 2BBP. | Read 2 | O/E - right eye background diabetic retinopathy |
| Diabetic retinopathy | 2BBQ. | Read 2 | O/E - left eye background diabetic retinopathy |
| Diabetic retinopathy | F4200 | Read 2 | Background diabetic retinopathy |
| Diabetic retinopathy | F4206 | Read 2 | Non proliferative diabetic retinopathy |
| Diabetic retinopathy | 2BBo. | Read 2 | O/E - sight threatening diabetic retinopathy |
| Diabetic retinopathy | 2BBR. | Read 2 | O/E - right eye preproliferative diabetic retinopathy |
| Diabetic retinopathy | 2BBS. | Read 2 | O/E - left eye preproliferative diabetic retinopathy |
| Diabetic retinopathy | F4202 | Read 2 | Preproliferative diabetic retinopathy |
| Diabetic retinopathy | F4208 | Read 2 | High risk non proliferative diabetic retinopathy |
| Diabetic retinopathy | 2BBr. | Read 2 | Impaired vision due to diabetic retinopathy |
| Diabetic retinopathy | 2BBk. | Read 2 | O/E - right eye stable treated proliferative diabetic retinopathy |
| Diabetic retinopathy | 2BBl. | Read 2 | O/E - left eye stable treated proliferative diabetic retinopathy |
| Diabetic retinopathy | 2BBT. | Read 2 | O/E - right eye proliferative diabetic retinopathy |
| Diabetic retinopathy | 2BBV. | Read 2 | O/E - left eye proliferative diabetic retinopathy |
| Diabetic retinopathy | 7276. | Read 2 | Pan retinal photocoagulation for diabetes |
| Diabetic retinopathy | F4201 | Read 2 | Proliferative diabetic retinopathy |
| Diabetic retinopathy | F4205 | Read 2 | Advanced diabetic retinal disease |
| Diabetic retinopathy | F4207 | Read 2 | High risk proliferative diabetic retinopathy |
| Diabetic retinopathy | 2BBm. | Read 2 | O/E - right eye clinically significant macular oedema |
| Diabetic retinopathy | 2BBn. | Read 2 | O/E - left eye clinically significant macular oedema |
| Diabetic retinopathy | C10EP | Read 2 | Type 1 diabetes mellitus with exudative maculopathy |
| Diabetic retinopathy | C10FQ | Read 2 | Type 2 diabetes mellitus with exudative maculopathy |
| Diabetic retinopathy | F4203 | Read 2 | Advanced diabetic maculopathy |
| Diabetic retinopathy | 2BBL. | Read 2 | O/E - diabetic maculopathy present both eyes |
| Diabetic retinopathy | 2BBW. | Read 2 | O/E - right eye diabetic maculopathy |
| Diabetic retinopathy | 2BBX. | Read 2 | O/E - left eye diabetic maculopathy |
| Diabetic retinopathy | F4204 | Read 2 | Diabetic maculopathy |
| Diabetic retinopathy | C105. | Read 3 | Diabetes mellitus with ophthalmic manifestation |
| Diabetic retinopathy | C1050 | Read 3 | Diabetes mellitus, juvenile type, with ophthalmic manifestation |
| Diabetic retinopathy | C1051 | Read 3 | Diabetes mellitus, adult onset, with ophthalmic manifestation |
| Diabetic retinopathy | C105y | Read 3 | Other specified diabetes mellitus with ophthalmic complications |
| Diabetic retinopathy | C105z | Read 3 | Diabetes mellitus NOS with ophthalmic manifestation |
| Diabetic retinopathy | C1081 | Read 3 | Type I diabetes mellitus with ophthalmic complications |
| Diabetic retinopathy | C1087 | Read 3 | Type I diabetes mellitus with retinopathy |
| Diabetic retinopathy | C1091 | Read 3 | Type II diabetes mellitus with ophthalmic complications |
| Diabetic retinopathy | C1096 | Read 3 | Type II diabetes mellitus with retinopathy |
| Diabetic retinopathy | C10A3 | Read 3 | Malnutrition-related diabetes mellitus with ophthalmic complications |
| Diabetic retinopathy | F420. | Read 3 | Diabetic retinopathy |
| Diabetic retinopathy | F420z | Read 3 | Diabetic retinopathy NOS |
| Diabetic retinopathy | XE12G | Read 3 | Diabetes + eye manifestation (& [cataract] or [retinopathy]) |
| Diabetic retinopathy | XaBul | Read 3 | Retinal abnormality - diabetes-related |
| Diabetic retinopathy | XaXfs | Read 3 | Diabetic retinopathy detected by national screening programme |
| Diabetic retinopathy | F4200 | Read 3 | Background diabetic retinopathy |
| Diabetic retinopathy | XaE5T | Read 3 | Mild non proliferative diabetic retinopathy |
| Diabetic retinopathy | XaIP5 | Read 3 | Non proliferative diabetic retinopathy |
| Diabetic retinopathy | XaJOg | Read 3 | O/E - right eye background diabetic retinopathy |
| Diabetic retinopathy | XaJOh | Read 3 | O/E - left eye background diabetic retinopathy |
| Diabetic retinopathy | F4202 | Read 3 | Preproliferative diabetic retinopathy |
| Diabetic retinopathy | X00dF | Read 3 | Visually threatening diabetic retinopathy |
| Diabetic retinopathy | XaE5U | Read 3 | Moderate non proliferative diabetic retinopathy |
| Diabetic retinopathy | XaE5V | Read 3 | Severe non proliferative diabetic retinopathy |
| Diabetic retinopathy | XaIW8 | Read 3 | High risk non proliferative diabetic retinopathy |
| Diabetic retinopathy | XaJOi | Read 3 | O/E - right eye preproliferative diabetic retinopathy |
| Diabetic retinopathy | XaJOj | Read 3 | O/E - left eye preproliferative diabetic retinopathy |
| Diabetic retinopathy | XaKcS | Read 3 | O/E - sight threatening diabetic retinopathy |
| Diabetic retinopathy | XaPen | Read 3 | Impaired vision due to diabetic retinopathy |
| Diabetic retinopathy | F4201 | Read 3 | Proliferative diabetic retinopathy |
| Diabetic retinopathy | X00dH | Read 3 | Proliferative diabetic retinopathy new vessels on disc |
| Diabetic retinopathy | X00dI | Read 3 | Proliferative diabetic retinopathy with new vessels elsewhere than on disc |
| Diabetic retinopathy | X00dJ | Read 3 | Diabetic traction retinal detachment |
| Diabetic retinopathy | XaD2T | Read 3 | Advanced diabetic retinal disease |
| Diabetic retinopathy | XaE5W | Read 3 | Proliferative diabetic retinopathy - non high risk |
| Diabetic retinopathy | XaE5X | Read 3 | Proliferative diabetic retinopathy - high risk |
| Diabetic retinopathy | XaE5Y | Read 3 | Proliferative diabetic retinopathy - quiescent |
| Diabetic retinopathy | XaE5Z | Read 3 | Proliferative diabetic retinopathy - iris neovascularisation |
| Diabetic retinopathy | XaJOk | Read 3 | O/E - right eye proliferative diabetic retinopathy |
| Diabetic retinopathy | XaJOl | Read 3 | O/E - left eye proliferative diabetic retinopathy |
| Diabetic retinopathy | XaJlI | Read 3 | Pan retinal photocoagulation for diabetes |
| Diabetic retinopathy | XaKDG | Read 3 | O/E - right eye stable treated proliferative diabetic retinopathy |
| Diabetic retinopathy | XaKDH | Read 3 | O/E - left eye stable treated proliferative diabetic retinopathy |
| Diabetic retinopathy | F4203 | Read 3 | Advanced diabetic maculopathy |
| Diabetic retinopathy | XaE5b | Read 3 | Clinically significant macular oedema |
| Diabetic retinopathy | XaE5c | Read 3 | Diabetic macular oedema |
| Diabetic retinopathy | XaEVO | Read 3 | Diffuse diabetic maculopathy |
| Diabetic retinopathy | XaEVP | Read 3 | Focal diabetic maculopathy |
| Diabetic retinopathy | XaEVS | Read 3 | Ischaemic diabetic maculopathy |
| Diabetic retinopathy | XaEVT | Read 3 | Mixed diabetic maculopathy |
| Diabetic retinopathy | XaJQp | Read 3 | Type II diabetes mellitus with exudative maculopathy |
| Diabetic retinopathy | XaJSr | Read 3 | Type 1 diabetes mellitus with exudative maculopathy |
| Diabetic retinopathy | XaKDI | Read 3 | O/E - right eye clinically significant macular oedema |
| Diabetic retinopathy | XaKDJ | Read 3 | O/E - left eye clinically significant macular oedema |
| Diabetic retinopathy | X00dG | Read 3 | Diabetic maculopathy |
| Diabetic retinopathy | XaIPk | Read 3 | O/E - diabetic maculopathy present both eyes |
| Diabetic retinopathy | XaJOn | Read 3 | O/E - right eye diabetic maculopathy |
| Diabetic retinopathy | XaJOo | Read 3 | O/E - left eye diabetic maculopathy |
| Glaucoma | 1436 | Data coding 5 | glaucoma surgery/trabeculectomy |
| Glaucoma | 1277 | Data coding 6 | glaucoma |
| Glaucoma | Q150 | ICD-10 | Congenital glaucoma |
| Glaucoma | H40 | ICD-10 | Glaucoma |
| Glaucoma | H409 | ICD-10 | Glaucoma, unspecified |
| Glaucoma | H402 | ICD-10 | Primary angle-closure glaucoma |
| Glaucoma | H401 | ICD-10 | Primary open-angle glaucoma |
| Glaucoma | H403 | ICD-10 | Glaucoma secondary to eye trauma |
| Glaucoma | H404 | ICD-10 | Glaucoma secondary to eye inflammation |
| Glaucoma | H405 | ICD-10 | Glaucoma secondary to other eye disorders |
| Glaucoma | H406 | ICD-10 | Glaucoma secondary to drugs |
| Glaucoma | H408 | ICD-10 | Other glaucoma |
| Glaucoma | H42 | ICD-10 | Glaucoma in diseases classified elsewhere |
| Glaucoma | H420 | ICD-10 | Glaucoma in endocrine, nutritional and metabolic diseases |
| Glaucoma | H428 | ICD-10 | Glaucoma in other diseases classified elsewhere |
| Glaucoma | 365 | ICD-9 | GLAUCOMA |
| Glaucoma | 3659 | ICD-9 | UNSPECIFIED |
| Glaucoma | V801 | ICD-9 | GLAUCOMA |
| Glaucoma | 3652 | ICD-9 | PRIMARY ANGLE-CLOSURE GLAUCOMA |
| Glaucoma | 3653 | ICD-9 | CORTICOSTEROID-INDUCED GLAUCOMA |
| Glaucoma | 3654 | ICD-9 | GLAUCOMA ASSOCIATED WITH CONGENITAL ANOMALIES, WIT |
| Glaucoma | 3654A | ICD-9 | GLAUCOMA ASSOCIATED WITH CONGENITAL ANOMALIES, WITH DYSTROPH |
| Glaucoma | 3655 | ICD-9 | GLAUCOMA ASSOCIATED WITH DISORDERS OF THE LENS |
| Glaucoma | 3656 | ICD-9 | GLAUCOMA ASSOCIATED WITH OTHER OCULAR DISORDERS |
| Glaucoma | 3658 | ICD-9 | OTHER GLAUCOMA |
| Glaucoma | 3651 | ICD-9 | OPEN-ANGLE GLAUCOMA |
| Glaucoma | C601 | OPCS-4 | C60.1 Trabeculectomy |
| Glaucoma | C605 | OPCS-4 | C60.5 Insertion of tube into anterior chamber of eye to assist drainage of aqueous humour |
| Glaucoma | C606 | OPCS-4 | C60.6 Viscocanulostomy |
| Glaucoma | C61 | OPCS-4 | C61 Other operations on trabecular meshwork of eye |
| Glaucoma | C611 | OPCS-4 | C61.1 Laser trabeculoplasty |
| Glaucoma | C612 | OPCS-4 | C61.2 Trabeculotomy |
| Glaucoma | C613 | OPCS-4 | C61.3 Goniotomy |
| Glaucoma | C614 | OPCS-4 | C61.4 Goniopuncture |
| Glaucoma | C615 | OPCS-4 | C61.5 Viscogonioplasty |
| Glaucoma | C618 | OPCS-4 | C61.8 Other specified other operations on trabecular meshwork of eye |
| Glaucoma | C619 | OPCS-4 | C61.9 Unspecified other operations on trabecular meshwork of eye |
| Glaucoma | C65 | OPCS-4 | C65 Operations following glaucoma surgery |
| Glaucoma | C654 | OPCS-4 | C65.4 Removal of releasable suture following glaucoma surgery |
| Glaucoma | C655 | OPCS-4 | C65.5 Laser suture lysis following glaucoma surgery |
| Glaucoma | C658 | OPCS-4 | C65.8 Other specified operations following glaucoma surgery |
| Glaucoma | C659 | OPCS-4 | C65.9 Unspecified operations following glaucoma surgery |
| Glaucoma | C663 | OPCS-4 | C66.3 Cryotherapy to ciliary body |
| Glaucoma | C664 | OPCS-4 | C66.4 Laser photocoagulation of ciliary body |
| Glaucoma | C665 | OPCS-4 | C66.5 Destruction of ciliary body NEC |
| Glaucoma | F4514 | Read 2 | Glaucoma of childhood |
| Glaucoma | P3200 | Read 2 | Congenital glaucoma |
| Glaucoma | Q20y7 | Read 2 | Traumatic glaucoma due to birth trauma |
| Glaucoma | 72550 | Read 2 | Trabeculectomy |
| Glaucoma | 72554 | Read 2 | Insertion of tube into anterior chamber of eye to assist drainage of aqueous humour |
| Glaucoma | 72555 | Read 2 | Viscocanulostomy |
| Glaucoma | 72556 | Read 2 | Revision of trabeculectomy |
| Glaucoma | 7256. | Read 2 | Other operations on trabecular meshwork of eye |
| Glaucoma | 72560 | Read 2 | Laser trabeculoplasty |
| Glaucoma | 72561 | Read 2 | Trabeculotomy |
| Glaucoma | 72562 | Read 2 | Goniotomy |
| Glaucoma | 72563 | Read 2 | Goniopuncture |
| Glaucoma | 72564 | Read 2 | Viscogonioplasty |
| Glaucoma | 7256y | Read 2 | Other specified other operation on trabecular meshwork of eye |
| Glaucoma | 7256z | Read 2 | Other operation on trabecular meshwork of eye NOS |
| Glaucoma | 72587 | Read 2 | Resuturing of trabeculectomy |
| Glaucoma | 7259. | Read 2 | Operations following glaucoma surgery |
| Glaucoma | 72590 | Read 2 | Needling of bleb following glaucoma surgery |
| Glaucoma | 72591 | Read 2 | Injection of bleb following glaucoma surgery |
| Glaucoma | 72592 | Read 2 | Revision of bleb NEC following glaucoma surgery |
| Glaucoma | 72593 | Read 2 | Removal of releasable suture following glaucoma surgery |
| Glaucoma | 72594 | Read 2 | Laser suture lysis following glaucoma surgery |
| Glaucoma | 7259y | Read 2 | Other specified operations following glaucoma surgery |
| Glaucoma | 7259z | Read 2 | Operations following glaucoma surgery NOS |
| Glaucoma | 72601 | Read 2 | Cryotherapy to ciliary body |
| Glaucoma | 72603 | Read 2 | Laser photocoagulation of ciliary body |
| Glaucoma | 72604 | Read 2 | Destruction of ciliary body NEC |
| Glaucoma | 7275. | Read 2 | Pan retinal photocoagulation for glaucoma |
| Glaucoma | F45.. | Read 2 | Glaucoma |
| Glaucoma | F45z. | Read 2 | Glaucoma NOS |
| Glaucoma | F4H14 | Read 2 | Optic disc glaucomatous atrophy |
| Glaucoma | FyuG. | Read 2 | [X]Glaucoma |
| Glaucoma | 1482. | Read 2 | H/O: glaucoma |
| Glaucoma | F452. | Read 2 | Primary angle-closure glaucoma |
| Glaucoma | F4520 | Read 2 | Unspecified primary angle-closure glaucoma |
| Glaucoma | F4521 | Read 2 | Intermittent primary angle-closure glaucoma |
| Glaucoma | F4522 | Read 2 | Acute primary angle-closure glaucoma |
| Glaucoma | F4523 | Read 2 | Chronic primary angle-closure glaucoma |
| Glaucoma | F4524 | Read 2 | Primary angle-closure glaucoma residual stage |
| Glaucoma | F452z | Read 2 | Primary angle-closure glaucoma NOS |
| Glaucoma | F4511 | Read 2 | Primary open-angle glaucoma |
| Glaucoma | F4512 | Read 2 | Low tension glaucoma |
| Glaucoma | F45y2 | Read 2 | Low tension glaucoma |
| Glaucoma | F4042 | Read 2 | Blind hypertensive eye |
| Glaucoma | F4421 | Read 2 | Glaucomatocyclitic crises |
| Glaucoma | F4513 | Read 2 | Pigmentary glaucoma |
| Glaucoma | F453. | Read 2 | Steroid-induced glaucoma |
| Glaucoma | F4530 | Read 2 | Steroid-induced glaucoma glaucomatous stage |
| Glaucoma | F4531 | Read 2 | Steroid-induced glaucoma residual stage |
| Glaucoma | F453z | Read 2 | Steroid-induced glaucoma NOS |
| Glaucoma | F454. | Read 2 | Glaucoma due to disease EC |
| Glaucoma | F4540 | Read 2 | Glaucoma due to chamber angle anomaly |
| Glaucoma | F4541 | Read 2 | Glaucoma due to iris anomaly |
| Glaucoma | F4542 | Read 2 | Glaucoma due to other anterior segment anomaly |
| Glaucoma | F4543 | Read 2 | Glaucoma due to systemic syndrome |
| Glaucoma | F4544 | Read 2 | Glaucoma in endocrine, nutritional and metabolic diseases |
| Glaucoma | F454z | Read 2 | Glaucoma due to disease NOS |
| Glaucoma | F455. | Read 2 | Glaucoma associated with disorders of the lens |
| Glaucoma | F4550 | Read 2 | Phacolytic glaucoma |
| Glaucoma | F4551 | Read 2 | Pseudoexfoliation glaucoma |
| Glaucoma | F455z | Read 2 | Glaucoma associated with disorders of the lens NOS |
| Glaucoma | F456. | Read 2 | Glaucoma associated with other ocular disorders |
| Glaucoma | F4560 | Read 2 | Glaucoma due to unspecified ocular disorder |
| Glaucoma | F4561 | Read 2 | Glaucoma due to pupillary block |
| Glaucoma | F4562 | Read 2 | Glaucoma due to ocular inflammation |
| Glaucoma | F4563 | Read 2 | Glaucoma due to ocular vascular disorder |
| Glaucoma | F4564 | Read 2 | Glaucoma due to ocular tumour or cyst |
| Glaucoma | F4565 | Read 2 | Glaucoma due to ocular trauma |
| Glaucoma | F4566 | Read 2 | Neovascular glaucoma |
| Glaucoma | F456z | Read 2 | Glaucoma associated with other ocular disorders NOS |
| Glaucoma | F45y. | Read 2 | Other specified forms of glaucoma |
| Glaucoma | F45y0 | Read 2 | Hypersecretion glaucoma |
| Glaucoma | F45y1 | Read 2 | Glaucoma due to episode of increased venous pressure |
| Glaucoma | F45yz | Read 2 | Other specified glaucoma NOS |
| Glaucoma | FyuG0 | Read 2 | [X]Other glaucoma |
| Glaucoma | FyuG1 | Read 2 | [X]Glaucoma in endocrine, nutritional and metabolic diseases classified elsewhere |
| Glaucoma | FyuG2 | Read 2 | [X]Glaucoma in other diseases classified elsewhere |
| Glaucoma | F4631 | Read 2 | Glaucomatous subcapsular flecks |
| Glaucoma | F4501 | Read 2 | Open angle glaucoma with borderline intraocular pressure |
| Glaucoma | F451. | Read 2 | Open-angle glaucoma |
| Glaucoma | F4510 | Read 2 | Unspecified open-angle glaucoma |
| Glaucoma | F4515 | Read 2 | Open-angle glaucoma residual stage |
| Glaucoma | F451z | Read 2 | Open-angle glaucoma NOS |
| Glaucoma | F4514 | Read 3 | Glaucoma of childhood |
| Glaucoma | P32.. | Read 3 | Congenital glaucoma |
| Glaucoma | P3421 | Read 3 | Irido-corneo-trabecular dysgenesis |
| Glaucoma | P3422 | Read 3 | Irido-trabecular dysgenesis |
| Glaucoma | Q20y7 | Read 3 | Traumatic glaucoma due to birth trauma |
| Glaucoma | X75kE | Read 3 | Goniodysgenesis iris ciliary body abnormal |
| Glaucoma | X77NV | Read 3 | Goniodysgenesis |
| Glaucoma | X77t4 | Read 3 | Trabecular dysgenesis |
| Glaucoma | .N141 | Read 3 | Congenital glaucoma |
| Glaucoma | P3200 | Read 3 | Congenital glaucoma |
| Glaucoma | 72550 | Read 3 | Trabeculectomy |
| Glaucoma | 72554 | Read 3 | Insertion anterior chamber drainage tube (& Molteno tube) |
| Glaucoma | 7256. | Read 3 | Other operations on trabecular meshwork of eye |
| Glaucoma | 72560 | Read 3 | Laser trabeculoplasty |
| Glaucoma | 72561 | Read 3 | Trabeculotomy |
| Glaucoma | 72562 | Read 3 | Goniotomy (& Barkan) |
| Glaucoma | 72563 | Read 3 | Goniopuncture (& Barkan) |
| Glaucoma | 7256y | Read 3 | Other specified other operation on trabecular meshwork of eye |
| Glaucoma | 7256z | Read 3 | Other operation on trabecular meshwork of eye NOS |
| Glaucoma | 7260. | Read 3 | Destruction of ciliary body |
| Glaucoma | 72603 | Read 3 | Laser coagulation of ciliary body |
| Glaucoma | 72604 | Read 3 | Destruction of ciliary body NEC |
| Glaucoma | 7275. | Read 3 | Panretinal photocoagulation for glaucoma |
| Glaucoma | X00Wr | Read 3 | Insertion of Molteno tube into anterior chamber |
| Glaucoma | X00X4 | Read 3 | Trabecular meshwork operation |
| Glaucoma | X00X5 | Read 3 | Trabeculoplasty |
| Glaucoma | X00X6 | Read 3 | Surgical trabeculoplasty |
| Glaucoma | X00X8 | Read 3 | Ultrasound destruction of ciliary body |
| Glaucoma | X00XI | Read 3 | Cataract extraction, insertion of intraocular lens and trabeculectomy |
| Glaucoma | XE0BO | Read 3 | Goniotomy |
| Glaucoma | XE0BP | Read 3 | Goniopuncture |
| Glaucoma | XE0Kk | Read 3 | Intraocular tension relief (& glaucoma) |
| Glaucoma | XE0Km | Read 3 | (Iridectomy) or (trabeculectomy) |
| Glaucoma | XE0Ko | Read 3 | (Iridotomy) or (trabeculotomy) |
| Glaucoma | XaE6n | Read 3 | Viscocanulostomy |
| Glaucoma | XaEXv | Read 3 | Revision of trabeculectomy |
| Glaucoma | XaEYc | Read 3 | Resuturing of trabeculectomy |
| Glaucoma | XaKSk | Read 3 | Adjustment of trabeculectomy suture |
| Glaucoma | XaKaz | Read 3 | Trabeculectomy with intraoperative application of 5-fluorouracil |
| Glaucoma | XaKb1 | Read 3 | Trabeculectomy with intraoperative application of mitomycin |
| Glaucoma | XaKb3 | Read 3 | Trabeculectomy with beta-irradiation |
| Glaucoma | XaKb6 | Read 3 | Removal of glaucoma drainage tube |
| Glaucoma | XaKb8 | Read 3 | Removal of Molteno tube |
| Glaucoma | XaKbh | Read 3 | Removal of releasable trabeculectomy suture |
| Glaucoma | XaKbp | Read 3 | Adjustment of suture within Molteno tube |
| Glaucoma | XaL58 | Read 3 | Operations following glaucoma surgery |
| Glaucoma | XaL59 | Read 3 | Removal of releasable suture following glaucoma surgery |
| Glaucoma | XaL5A | Read 3 | Laser suture lysis following glaucoma surgery |
| Glaucoma | XaL5B | Read 3 | Other specified operations following glaucoma surgery |
| Glaucoma | XaL5C | Read 3 | Operations following glaucoma surgery NOS |
| Glaucoma | XaL5m | Read 3 | Needling of bleb following glaucoma surgery |
| Glaucoma | XaL5n | Read 3 | Injection of bleb following glaucoma surgery |
| Glaucoma | XaL5o | Read 3 | Revision of bleb NEC following glaucoma surgery |
| Glaucoma | XaXCJ | Read 3 | Viscogonioplasty |
| Glaucoma | .7381 | Read 3 | Intraocular tension relief (& glaucoma) |
| Glaucoma | .7384 | Read 3 | (Iridectomy) or (trabeculectomy) |
| Glaucoma | .7385 | Read 3 | (Iridotomy) or (trabeculotomy) |
| Glaucoma | 72556 | Read 3 | Revision of trabeculectomy |
| Glaucoma | 72557 | Read 3 | Viscocanulostomy |
| Glaucoma | 72564 | Read 3 | Viscogonioplasty |
| Glaucoma | 72587 | Read 3 | Resuturing of trabeculectomy |
| Glaucoma | 7259. | Read 3 | Operations following glaucoma surgery |
| Glaucoma | 72590 | Read 3 | Needling of bleb following glaucoma surgery |
| Glaucoma | 72591 | Read 3 | Injection of bleb following glaucoma surgery |
| Glaucoma | 72592 | Read 3 | Revision of bleb NEC following glaucoma surgery |
| Glaucoma | 72593 | Read 3 | Removal of releasable suture following glaucoma surgery |
| Glaucoma | 72594 | Read 3 | Laser suture lysis following glaucoma surgery |
| Glaucoma | 7259y | Read 3 | Other specified operations following glaucoma surgery |
| Glaucoma | 7259z | Read 3 | Operations following glaucoma surgery NOS |
| Glaucoma | F45.. | Read 3 | Glaucoma |
| Glaucoma | F45z. | Read 3 | Glaucoma NOS |
| Glaucoma | F4H14 | Read 3 | Glaucomatous optic atrophy |
| Glaucoma | .F54. | Read 3 | Glaucoma |
| Glaucoma | FyuG. | Read 3 | Glaucoma |
| Glaucoma | 1482. | Read 3 | H/O: glaucoma |
| Glaucoma | .1482 | Read 3 | H/O: glaucoma |
| Glaucoma | F452. | Read 3 | Glaucoma: [primary angle-closure] or [closed angle] |
| Glaucoma | F4520 | Read 3 | Unspecified primary angle-closure glaucoma |
| Glaucoma | F4521 | Read 3 | Intermittent angle-closure glaucoma |
| Glaucoma | F4524 | Read 3 | Primary angle-closure glaucoma residual stage |
| Glaucoma | F452z | Read 3 | Primary angle-closure glaucoma NOS |
| Glaucoma | XE2a8 | Read 3 | Primary angle-closure glaucoma |
| Glaucoma | .F543 | Read 3 | Primary angle-closure glaucoma |
| Glaucoma | F4511 | Read 3 | Primary open-angle glaucoma |
| Glaucoma | F4512 | Read 3 | Low tension glaucoma |
| Glaucoma | XaF9D | Read 3 | Primary open-angle glaucoma with narrow angles |
| Glaucoma | .F542 | Read 3 | Primary open-angle glaucoma |
| Glaucoma | .F544 | Read 3 | Low tension glaucoma |
| Glaucoma | F45y2 | Read 3 | Low tension glaucoma |
| Glaucoma | F4042 | Read 3 | (Blind hypertensive eye) or (glaucoma absolute) |
| Glaucoma | F4421 | Read 3 | Posner-Schlossman syndrome |
| Glaucoma | F4513 | Read 3 | Pigmentary glaucoma |
| Glaucoma | F453. | Read 3 | Steroid-induced glaucoma |
| Glaucoma | F4530 | Read 3 | Steroid-induced glaucoma glaucomatous stage |
| Glaucoma | F4531 | Read 3 | Steroid-induced glaucoma residual stage |
| Glaucoma | F453z | Read 3 | Steroid-induced glaucoma NOS |
| Glaucoma | F454. | Read 3 | Glaucoma due to disease EC |
| Glaucoma | F4540 | Read 3 | Glaucoma due to chamber angle anomaly |
| Glaucoma | F4541 | Read 3 | Glaucoma due to iris anomaly |
| Glaucoma | F4542 | Read 3 | Glaucoma due to other anterior segment anomaly |
| Glaucoma | F4543 | Read 3 | Glaucoma due to systemic syndrome |
| Glaucoma | F4544 | Read 3 | Glaucoma in endocrine, nutritional and metabolic diseases |
| Glaucoma | F454z | Read 3 | Glaucoma due to disease NOS |
| Glaucoma | F455. | Read 3 | Glaucoma with lens disorder |
| Glaucoma | F4550 | Read 3 | Phacolytic glaucoma |
| Glaucoma | F4551 | Read 3 | Pseudoexfoliation glaucoma |
| Glaucoma | F455z | Read 3 | Glaucoma associated with disorders of the lens NOS |
| Glaucoma | F456. | Read 3 | Glaucoma associated with other ocular disorders |
| Glaucoma | F4560 | Read 3 | Glaucoma due to unspecified ocular disorder |
| Glaucoma | F4561 | Read 3 | Secondary angle-closure glaucoma with pupil block |
| Glaucoma | F4562 | Read 3 | Glaucoma with ocular inflammation |
| Glaucoma | F4563 | Read 3 | Glaucoma due to ocular vascular disorder |
| Glaucoma | F4564 | Read 3 | Glaucoma due to ocular tumour &/or cyst |
| Glaucoma | F4565 | Read 3 | Glaucoma due to ocular trauma |
| Glaucoma | F456z | Read 3 | Glaucoma associated with other ocular disorders NOS |
| Glaucoma | F45y. | Read 3 | Other specified forms of glaucoma |
| Glaucoma | F45y0 | Read 3 | Hypersecretion glaucoma |
| Glaucoma | F45y1 | Read 3 | Glaucoma due to raised episcleral venous pressure |
| Glaucoma | F45yz | Read 3 | Other specified glaucoma NOS |
| Glaucoma | FyuG0 | Read 3 | [X]Other glaucoma |
| Glaucoma | FyuG1 | Read 3 | [X]Glaucoma in endocrine, nutritional and metabolic diseases classified elsewhere |
| Glaucoma | FyuG2 | Read 3 | [X]Glaucoma in other diseases classified elsewhere |
| Glaucoma | X00ee | Read 3 | Mixed open and angle-closure glaucoma |
| Glaucoma | X00ef | Read 3 | Absolute glaucoma |
| Glaucoma | X00eg | Read 3 | Iatrogenic glaucoma |
| Glaucoma | X00eh | Read 3 | Iatrogenic angle-closure glaucoma |
| Glaucoma | X00ei | Read 3 | Glaucoma and corneal anomaly |
| Glaucoma | X00ek | Read 3 | Chandler iris naevus syndrome |
| Glaucoma | X00el | Read 3 | Phacogenic glaucoma |
| Glaucoma | X00em | Read 3 | Neovascular glaucoma |
| Glaucoma | X00en | Read 3 | Glaucoma with intraocular haemorrhage |
| Glaucoma | X00eo | Read 3 | Ghost cell glaucoma |
| Glaucoma | X00eq | Read 3 | Glaucoma due to contusion injury |
| Glaucoma | X00er | Read 3 | Angle recession glaucoma |
| Glaucoma | X00es | Read 3 | Glaucoma following surgery |
| Glaucoma | X00et | Read 3 | Ciliary block glaucoma |
| Glaucoma | XE15r | Read 3 | Blind hypertensive eye |
| Glaucoma | XE16F | Read 3 | Glaucoma due to ocular tumour or cyst |
| Glaucoma | XM1QA | Read 3 | Glaucoma due to ocular cyst |
| Glaucoma | Xa3fE | Read 3 | Secondary/other glaucoma |
| Glaucoma | Xa9BT | Read 3 | Secondary glaucoma |
| Glaucoma | XaEVp | Read 3 | Uveitic glaucoma |
| Glaucoma | XaF9I | Read 3 | Secondary open-angle glaucoma |
| Glaucoma | XaF9J | Read 3 | Secondary angle-closure glaucoma |
| Glaucoma | XaFao | Read 3 | Secondary angle-closure glaucoma - synechial |
| Glaucoma | XaFap | Read 3 | Secondary angle-closure glaucoma with rubeosis |
| Glaucoma | XaI97 | Read 3 | Glaucoma due to silicon oil |
| Glaucoma | .F54Z | Read 3 | Secondary/other glaucoma |
| Glaucoma | F448. | Read 3 | Pigmentary glaucoma |
| Glaucoma | F4566 | Read 3 | Neovascular glaucoma |
| Glaucoma | F4522 | Read 3 | Acute angle-closure glaucoma |
| Glaucoma | F4523 | Read 3 | Chronic angle-closure glaucoma |
| Glaucoma | F4631 | Read 3 | Glaucomatous subcapsular flecks |
| Glaucoma | XaEVW | Read 3 | Acute-on-chronic glaucoma |
| Glaucoma | F4501 | Read 3 | Open-angle glaucoma - borderline |
| Glaucoma | F451. | Read 3 | Open-angle glaucoma |
| Glaucoma | F4510 | Read 3 | Unspecified open-angle glaucoma |
| Glaucoma | F4515 | Read 3 | Open-angle glaucoma residual stage |
| Glaucoma | F451z | Read 3 | Open-angle glaucoma NOS |
| Hypertension | 1065 | Data coding 6 | hypertension |
| Hypertension | 1072 | Data coding 6 | essential hypertension |
| Hypertension | 1073 | Data coding 6 | gestational hypertension/pre-eclampsia |
| Hypertension | I10 | ICD-10 | Essential (primary) hypertension |
| Hypertension | I11 | ICD-10 | Hypertensive heart disease |
| Hypertension | I12 | ICD-10 | Hypertensive renal disease |
| Hypertension | I13 | ICD-10 | Hypertensive heart and renal disease |
| Hypertension | I110 | ICD-10 | Hypertensive heart disease with (congestive) heart failure |
| Hypertension | I119 | ICD-10 | Hypertensive heart disease without (congestive) heart failure |
| Hypertension | I120 | ICD-10 | Hypertensive renal disease with renal failure |
| Hypertension | I129 | ICD-10 | Hypertensive renal disease without renal failure |
| Hypertension | I130 | ICD-10 | Hypertensive heart and renal disease with (congestive) heart failure |
| Hypertension | I131 | ICD-10 | Hypertensive heart and renal disease with renal failure |
| Hypertension | I132 | ICD-10 | Hypertensive heart and renal disease with both (congestive) heart failure and renal failure |
| Hypertension | I139 | ICD-10 | Hypertensive heart and renal disease, unspecified |
| Hypertension | I15 | ICD-10 | Secondary hypertension |
| Hypertension | I150 | ICD-10 | Renovascular hypertension |
| Hypertension | I151 | ICD-10 | Hypertension secondary to other renal disorders |
| Hypertension | I152 | ICD-10 | Hypertension secondary to endocrine disorders |
| Hypertension | I158 | ICD-10 | Other secondary hypertension |
| Hypertension | I159 | ICD-10 | Secondary hypertension, unspecified |
| Hypertension | 4010 | ICD-9 | MALIGNANT ESSENTIAL HYPERTENSION |
| Hypertension | 4011 | ICD-9 | BENIGN ESSENTIAL HYPERTENSION |
| Hypertension | 4019 | ICD-9 | ESSENTIAL HYPERTENSION NOT SPECIFIED |
| Hypertension | 4020 | ICD-9 | MALIGNANT HYPERTENSIVE HEART DISEASE |
| Hypertension | 4021 | ICD-9 | BENIGN HYPERTENSIVE HEART DISEASE |
| Hypertension | 4029 | ICD-9 | HYPERTENSIVE HEART DISEASE NOT SPECIFIED |
| Hypertension | 4030 | ICD-9 | MALIGNANT HYPERTENSIVE RENAL DISEASE |
| Hypertension | 4031 | ICD-9 | BENIGN HYPERTENSIVE RENAL DISEASE |
| Hypertension | 4039 | ICD-9 | HYPERTENSIVE RENAL DISEASE NOT SPECIFIED |
| Hypertension | 4040 | ICD-9 | MALIGNANT HYPERTENSIVE HEART AND RENAL DISEASE |
| Hypertension | 4041 | ICD-9 | BENIGN HYPERTENSIVE HEART AND RENAL DISEASE |
| Hypertension | 4049 | ICD-9 | HYPERTENSIVE HEART AND RENAL DISEASE NOT SPECIFIED |
| Hypertension | 4050 | ICD-9 | SPECIFIED AS MALIGNANT |
| Hypertension | 4051 | ICD-9 | SPECIFIED AS BENIGN |
| Hypertension | 4059 | ICD-9 | NOT SPECIFIED AS MALIGNANT OR BENIGN |
| Hypertension | 14A2. | Read 2 | H/O: hypertension |
| Hypertension | 21261 | Read 2 | Hypertension resolved |
| Hypertension | 212K. | Read 2 | Hypertension resolved |
| Hypertension | 9OI9. | Read 2 | Hypertens.monitor deleted |
| Hypertension | 6624. | Read 2 | Borderline hyperten:yearly obs |
| Hypertension | 6627. | Read 2 | Good hypertension control |
| Hypertension | 6628. | Read 2 | Poor hypertension control |
| Hypertension | 662b. | Read 2 | Moderate hypertension control |
| Hypertension | 662c. | Read 2 | Hypertension six month review |
| Hypertension | 662d. | Read 2 | Hypertension annual review |
| Hypertension | 662F. | Read 2 | Hypertension treatm. started |
| Hypertension | 662G. | Read 2 | Hypertensive treatm.changed |
| Hypertension | 662O. | Read 2 | On treatment for hypertension |
| Hypertension | 662r. | Read 2 | Trial withdrawal of antihypertensive therapy |
| Hypertension | 7Q01. | Read 2 | High cost hypertension drugs |
| Hypertension | 8B26. | Read 2 | Antihypertensive therapy |
| Hypertension | 8BL0. | Read 2 | Patient on maximal tolerated antihypertensive therapy |
| Hypertension | 8I3N. | Read 2 | Hypertension treatment refused |
| Hypertension | F4213 | Read 2 | Hypertensive retinopathy |
| Hypertension | G2... | Read 2 | Hypertensive disease |
| Hypertension | G20.. | Read 2 | Essential hypertension |
| Hypertension | G200. | Read 2 | Malignant essential hypertension |
| Hypertension | G201. | Read 2 | Benign essential hypertension |
| Hypertension | G202. | Read 2 | Systolic hypertension |
| Hypertension | G203. | Read 2 | Diastolic hypertension |
| Hypertension | G20z. | Read 2 | Essential hypertension NOS |
| Hypertension | G21.. | Read 2 | Hypertensive heart disease |
| Hypertension | G210. | Read 2 | Malignant hypertensive heart disease |
| Hypertension | G2100 | Read 2 | Malignant hypertensive heart disease without CCF |
| Hypertension | G2101 | Read 2 | Malignant hypertensive heart disease with CCF |
| Hypertension | G211. | Read 2 | Benign hypertensive heart disease |
| Hypertension | G2110 | Read 2 | Benign hypertensive heart disease without CCF |
| Hypertension | G2111 | Read 2 | Benign hypertensive heart disease with CCF |
| Hypertension | G21z. | Read 2 | Hypertensive heart disease NOS |
| Hypertension | G21z0 | Read 2 | Hypertensive heart disease NOS without CCF |
| Hypertension | G21z1 | Read 2 | Hypertensive heart disease NOS with CCF |
| Hypertension | G21zz | Read 2 | Hypertensive heart disease NOS |
| Hypertension | G22.. | Read 2 | Hypertensive renal disease |
| Hypertension | G220. | Read 2 | Malignant hypertensive renal disease |
| Hypertension | G221. | Read 2 | Benign hypertensive renal disease |
| Hypertension | G222. | Read 2 | Hypertensive renal disease with renal failure |
| Hypertension | G22z. | Read 2 | Hypertensive renal disease NOS |
| Hypertension | G23.. | Read 2 | Hypertensive heart and renal disease |
| Hypertension | G230. | Read 2 | Malignant hypertensive heart and renal disease |
| Hypertension | G231. | Read 2 | Benign hypertensive heart and renal disease |
| Hypertension | G232. | Read 2 | Hypertensive heart&renal dis wth (congestive) heart failure |
| Hypertension | G233. | Read 2 | Hypertensive heart and renal disease with renal failure |
| Hypertension | G234. | Read 2 | Hyperten heart&renal dis+both(congestv)heart and renal fail |
| Hypertension | G23z. | Read 2 | Hypertensive heart and renal disease NOS |
| Hypertension | G2y.. | Read 2 | Other specified hypertensive disease |
| Hypertension | G2z.. | Read 2 | Hypertensive disease NOS |
| Hypertension | G672. | Read 2 | Hypertensive encephalopathy |
| Hypertension | Gyu2. | Read 2 | [X]Hypertensive diseases |
| Hypertension | L122. | Read 2 | Other pre-existing hypertension in preg/childbirth/puerp |
| Hypertension | L1220 | Read 2 | Other pre-existing hypertension in preg/childb/puerp unspec |
| Hypertension | L1221 | Read 2 | Other pre-existing hypertension in preg/childb/puerp - deliv |
| Hypertension | L1223 | Read 2 | Other pre-exist hypertension in preg/childb/puerp-not deliv |
| Hypertension | L122z | Read 2 | Other pre-existing hypertension in preg/childb/puerp NOS |
| Hypertension | L127. | Read 2 | Pre-eclampsia or eclampsia with pre-existing hypertension |
| Hypertension | L127z | Read 2 | Pre-eclampsia or eclampsia + pre-existing hypertension NOS |
| Hypertension | L128. | Read 2 | Pre-exist hypertension compl preg childbirth and puerperium |
| Hypertension | L1280 | Read 2 | Pre-exist hyperten heart dis compl preg childbth+puerperium |
| Hypertension | L1282 | Read 2 | Pre-exist 2ndry hypertens comp preg childbth and puerperium |
| Hypertension | TJC7. | Read 2 | Adverse reaction to other antihypertensives |
| Hypertension | TJC7z | Read 2 | Adverse reaction to antihypertensives NOS |
| Hypertension | U60C5 | Read 2 | [X]Oth antihyperten drug caus advers eff in therap use, NEC |
| Hypertension | 61462 | Read 2 | Hypertension induced by oral contraceptive pill |
| Hypertension | G24.. | Read 2 | Secondary hypertension |
| Hypertension | G240. | Read 2 | Secondary malignant hypertension |
| Hypertension | G2400 | Read 2 | Secondary malignant renovascular hypertension |
| Hypertension | G240z | Read 2 | Secondary malignant hypertension NOS |
| Hypertension | G241. | Read 2 | Secondary benign hypertension |
| Hypertension | G2410 | Read 2 | Secondary benign renovascular hypertension |
| Hypertension | G241z | Read 2 | Secondary benign hypertension NOS |
| Hypertension | G244. | Read 2 | Hypertension secondary to endocrine disorders |
| Hypertension | G24z. | Read 2 | Secondary hypertension NOS |
| Hypertension | G24z0 | Read 2 | Secondary renovascular hypertension NOS |
| Hypertension | G24z1 | Read 2 | Hypertension secondary to drug |
| Hypertension | G24zz | Read 2 | Secondary hypertension NOS |
| Hypertension | Gyu21 | Read 2 | [X]Hypertension secondary to other renal disorders |
| Hypertension | 14A2. | Read 3 | H/O: hypertension |
| Hypertension | 21261 | Read 3 | Hypertension resolved |
| Hypertension | 6624. | Read 3 | Borderline hyperten:yearly obs |
| Hypertension | 6627. | Read 3 | Good hypertension control |
| Hypertension | 6628. | Read 3 | Poor hypertension control |
| Hypertension | XaIy8 | Read 3 | Moderate hypertension control |
| Hypertension | XaIyD | Read 3 | Hypertension six month review |
| Hypertension | XaIyE | Read 3 | Hypertension annual review |
| Hypertension | 662F. | Read 3 | Hypertension treatm. started |
| Hypertension | 662G. | Read 3 | Hypertensive treatm.changed |
| Hypertension | Xa8HD | Read 3 | On treatment for hypertension |
| Hypertension | XaNFs | Read 3 | Trial withdrawal of antihypertensive therapy |
| Hypertension | XaM5f | Read 3 | High cost hypertension drugs |
| Hypertension | XaBLq | Read 3 | Antihypertensive therapy |
| Hypertension | XaJ5h | Read 3 | Patient on maximal tolerated antihypertensive therapy |
| Hypertension | XaIyC | Read 3 | Hypertension treatment refused |
| Hypertension | F4211 | Read 3 | Hypertensive retinopathy |
| Hypertension | G2... | Read 3 | Hypertensive disease |
| Hypertension | XE0Uc | Read 3 | Essential hypertension |
| Hypertension | XM02V | Read 3 | Raised blood pressure |
| Hypertension | G200. | Read 3 | Malignant essential hypertension |
| Hypertension | G201. | Read 3 | Benign essential hypertension |
| Hypertension | G202. | Read 3 | Systolic hypertension |
| Hypertension | XSDSb | Read 3 | Diastolic hypertension |
| Hypertension | XE0Ud | Read 3 | Essential hypertension NOS |
| Hypertension | XE0Ub | Read 3 | Hypertension |
| Hypertension | G21.. | Read 3 | Hypertensive heart disease |
| Hypertension | G210. | Read 3 | Malignant hypertensive heart disease |
| Hypertension | G2100 | Read 3 | Malignant hypertensive heart disease without congestive cardiac failure |
| Hypertension | G2101 | Read 3 | Malignant hypertensive heart disease with congestive cardiac failure |
| Hypertension | G211. | Read 3 | Benign hypertensive heart disease |
| Hypertension | G2110 | Read 3 | Benign hypertensive heart disease without congestive cardiac failure |
| Hypertension | G2111 | Read 3 | Benign hypertensive heart disease with congestive cardiac failure |
| Hypertension | G21z. | Read 3 | Hypertensive heart disease NOS |
| Hypertension | XE0Ue | Read 3 | Hypertensive heart disease NOS without congestive cardiac failure |
| Hypertension | XM1Qp | Read 3 | Cardiomegaly - hypertensive |
| Hypertension | G21z1 | Read 3 | Hypertensive heart disease NOS with congestive cardiac failure |
| Hypertension | XE0Uf | Read 3 | Hypertensive renal disease |
| Hypertension | G220. | Read 3 | Malignant hypertensive renal disease |
| Hypertension | G221. | Read 3 | Benign hypertensive renal disease |
| Hypertension | G222. | Read 3 | Hypertensive renal disease with renal failure |
| Hypertension | XE0Ug | Read 3 | Hypertensive renal disease NOS |
| Hypertension | G23.. | Read 3 | Hypertensive heart and renal disease |
| Hypertension | G230. | Read 3 | Malignant hypertensive heart and renal disease |
| Hypertension | G231. | Read 3 | Benign hypertensive heart and renal disease |
| Hypertension | G232. | Read 3 | Hypertensive heart and renal disease with (congestive) heart failure |
| Hypertension | G233. | Read 3 | Hypertensive heart and renal disease with renal failure |
| Hypertension | G234. | Read 3 | Hypertensive heart and renal disease with both (congestive) heart failure and renal failure |
| Hypertension | G23z. | Read 3 | Hypertensive heart and renal disease NOS |
| Hypertension | G2y.. | Read 3 | Other specified hypertensive disease |
| Hypertension | G2z.. | Read 3 | Hypertensive disease NOS |
| Hypertension | XE0VM | Read 3 | Hypertensive encephalopathy |
| Hypertension | Gyu2. | Read 3 | [X]Hypertensive diseases |
| Hypertension | L122. | Read 3 | Other pre-existing hypertension in preg/childbirth/puerp |
| Hypertension | L1220 | Read 3 | Other pre-existing hypertension complicating pregnancy, childbirth and the puerperium unspecified |
| Hypertension | L1221 | Read 3 | Other pre-existing hypertension complicating pregnancy, childbirth and the puerperium - delivered |
| Hypertension | L1223 | Read 3 | Other pre-existing hypertension complicating pregnancy, childbirth and the puerperium - not delivered |
| Hypertension | L122z | Read 3 | Other pre-existing hypertension complicating pregnancy, childbirth and the puerperium NOS |
| Hypertension | L127. | Read 3 | Pre-eclampsia or eclampsia with pre-existing hypertension |
| Hypertension | L127z | Read 3 | Pre-eclampsia or eclampsia with pre-existing hypertension NOS |
| Hypertension | L128. | Read 3 | Pre-existing hypertension complicating pregnancy, childbirth and puerperium |
| Hypertension | L1280 | Read 3 | Pre-existing hypertensive heart disease complicating pregnancy, childbirth and the puerperium |
| Hypertension | L1282 | Read 3 | Pre-existing secondary hypertension complicating pregnancy, childbirth and puerperium |
| Hypertension | TJC7. | Read 3 | Adverse reaction to other antihypertensives |
| Hypertension | TJC7z | Read 3 | Adverse reaction to antihypertensives NOS |
| Hypertension | U60C5 | Read 3 | [X]Other antihypertensive drugs causing adverse effects in therapeutic use, not elsewhere classified |
| Hypertension | 61462 | Read 3 | Hypertension induced by oral contraceptive pill |
| Hypertension | G24.. | Read 3 | Secondary hypertension |
| Hypertension | G240. | Read 3 | Malignant secondary hypertension |
| Hypertension | G2400 | Read 3 | Secondary malignant renovascular hypertension |
| Hypertension | G240z | Read 3 | Secondary malignant hypertension NOS |
| Hypertension | G241. | Read 3 | Secondary benign hypertension |
| Hypertension | G2410 | Read 3 | Secondary benign renovascular hypertension |
| Hypertension | G241z | Read 3 | Secondary benign hypertension NOS |
| Hypertension | G244. | Read 3 | Hypertension secondary to endocrine disorders |
| Hypertension | G24z. | Read 3 | Secondary hypertension NOS |
| Hypertension | G24z0 | Read 3 | Secondary renovascular hypertension NOS |
| Hypertension | G24z1 | Read 3 | Hypertension secondary to drug |
| Hypertension | Gyu21 | Read 3 | [X]Hypertension secondary to other renal disorders |

**sTable 3.** Participant characteristics comparing individuals who had gradable retinal images (optical coherence tomography or colour photos) in both eyes with those where retinal images were either unavailable or ungradable in at least one eye. Characteristics were ascertained from self-reported data at study baseline and linked electronic health care records data (primary care, hospital episode statistics, death register) up until the time of retinal imaging, or 1st January 2010 for participants who did not undergo imaging. Continuous variables are summarised by mean (standard deviation) and compared using the Student’s t-test and One-Way ANOVA for two and more than two groups respectively. Categorical variables are summarised by number (percentage) and compared using Pearson’s Chi-squared test. Abbreviations: AMD = age-related macular degeneration, MA = microaneurysm.

| **Characteristic** | **No, N = 56,308** | **Yes, N = 12,206** | **p-value** |
| --- | --- | --- | --- |
| **Age** | 56.9 (8.2) | 59.2 (7.7) | **<0.001** |
| **Sex** |  |  | **<0.001** |
| Female | 31,123 (55.3%) | 6,107 (50.0%) |  |
| Male | 25,185 (44.7%) | 6,099 (50.0%) |  |
| **Ethnic background** |  |  | **<0.001** |
| White | 51,135 (91.4%) | 10,461 (86.5%) |  |
| Mixed | 481 (0.9%) | 119 (1.0%) |  |
| Asian or Asian British | 1,597 (2.9%) | 625 (5.2%) |  |
| Black or Black British | 1,633 (2.9%) | 580 (4.8%) |  |
| Chinese | 237 (0.4%) | 75 (0.6%) |  |
| Other ethnic group | 838 (1.5%) | 237 (2.0%) |  |
| Missing | 387 | 109 |  |
| **Townsend Deprivation Index** | -1.05 (2.98) | -0.84 (3.09) | **<0.001** |
| Missing | 73 | 11 |  |
| **On insulin** | 540 (1.0%) | 198 (1.6%) | **<0.001** |
| **On antihypertensive medication** | 11,113 (19.7%) | 3,144 (25.8%) | **<0.001** |
| **On cholesterol-lowering medication** | 9,880 (17.5%) | 2,808 (23.0%) | **<0.001** |
| **Diabetes diagnosis** | 2,914 (5.2%) | 912 (7.5%) | **<0.001** |
| **Hypertension diagnosis** | 14,749 (26.2%) | 3,975 (32.6%) | **<0.001** |
| **Age-related macular degeneration diagnosis** | 205 (0.4%) | 66 (0.5%) | **0.005** |
| **Glaucoma diagnosis** | 830 (1.5%) | 298 (2.4%) | **<0.001** |
| **Diabetic retinopathy diagnosis** | 500 (0.9%) | 178 (1.5%) | **<0.001** |

**sTable 4.** Summary of all graded retinal features at the person level and by eye (see supplementary table 1 for variable definitions and abbreviations). VisitDate is summarised as a range, continuous variables by mean (standard deviation) and categorical variables by number. Person level summaries of categorical variables describe the number of individuals where at least one eye has that feature recorded.

|  | **Person** | **Right eye** | **Left eye** |
| --- | --- | --- | --- |
| **VisitDate** | 2009-12-07 to 2010-07-21 | — | — |
| **CupDiscRatioComparison** | 0.04 (0.05) | — | — |
| **COL_Available** | 68,504 | 67,982 | 67,610 |
| **OCT_Available** | 68,497 | 67,922 | 67,578 |
| **COL_Quality** |  |  |  |
| CG | 10,436 | 6,962 | 7,370 |
| Fair | 47,575 | 39,191 | 39,639 |
| Good | 9,704 | 8,046 | 6,259 |
| Poor | 21,223 | 13,783 | 14,342 |
| **OCT_Quality** |  |  |  |
| CG | 905 | 538 | 548 |
| Fair | 53,176 | 47,029 | 47,623 |
| Good | 18,403 | 15,244 | 14,840 |
| Poor | 7,264 | 5,111 | 4,567 |
| **AbnormalitiesPresent** | 24,733 | 18,380 | 18,140 |
| **AMD** | 15,176 | 10,507 | 10,472 |
| **COL_IfAMD** |  |  |  |
| Drusen | 9,881 | 6,858 | 6,471 |
| Exud | 63 | 39 | 35 |
| GA | 17 | 10 | 11 |
| Pigment | 581 | 296 | 356 |
| **COL_DrusenInsideGrid** | 7,544 | 5,059 | 4,754 |
| **COL_SevereDrusen** |  |  |  |
| CG | 2 | 1 | 1 |
| GreaterThan125 | 1,586 | 992 | 894 |
| LessThan63 | 3,082 | 1,842 | 1,784 |
| SixtyThreeTo125 | 3,594 | 2,224 | 2,076 |
| **COL_DrusenOutsideGrid** | 5,864 | 4,034 | 3,835 |
| **COL_ReticularDrusen** |  |  |  |
| CG | 10 | 8 | 4 |
| No | 9,842 | 6,814 | 6,429 |
| Yes | 41 | 35 | 36 |
| **OCT_DrusenPresent** | 6,528 | 4,237 | 4,151 |
| **OCT_SubretDrusen** | 8,713 | 5,602 | 5,974 |
| **OCT_FocalAtrophy** | 432 | 222 | 269 |
| **OCT_CentralSubfieldThickness** | — | 245 (41) | 247 (40) |
| **COL_RetinalHaemorrhage** | 740 | 488 | 406 |
| **COL_SignsOfDR** | 264 | 209 | 193 |
| **COL_DRFeatures** |  |  |  |
| CWS | 86 | 67 | 53 |
| Ex | 147 | 109 | 90 |
| FP | 10 | 4 | 8 |
| HMA | 264 | 209 | 193 |
| IRMA | 19 | 12 | 11 |
| LS | 62 | 41 | 56 |
| MBH | 8 | 5 | 4 |
| NVD | 9 | 3 | 7 |
| NVE | 7 | 4 | 3 |
| PF | 1 | 0 | 1 |
| PVH | 1 | 0 | 1 |
| VB | 6 | 4 | 4 |
| VL | 9 | 6 | 4 |
| **COL_VisibleLaserTreatment** | 99 | 75 | 67 |
| **OCT_HyporeflectiveIntraretinalSpaces** | 929 | 551 | 575 |
| **COL_CupDiscRatio** | — | 0.41 (0.10) | 0.41 (0.09) |
| **COL_HaemorrhageOnDisc** | 282 | 176 | 120 |
| **COL_IfOpticDiscSuspicious** |  |  |  |
| Both (IRT AND Notch) | 29 | 18 | 15 |
| IRT | 152 | 91 | 79 |
| Notch | 53 | 22 | 34 |
| **Occlusion** | 153 | 88 | 69 |
| **IfOcclusion** |  |  |  |
| BRAO | 13 | 9 | 4 |
| BRVO | 102 | 62 | 41 |
| CRAO | 1 | 1 | 0 |
| CRVO | 40 | 17 | 24 |
| **EvidenceOfERM** | 7,996 | 5,583 | 5,890 |
| **IfERMStructuralChanges** | 1,550 | 906 | 922 |
| **OCT_EvidenceOfVMA** | 45,266 | 38,110 | 39,120 |
| **OCT_IfVMACategories** |  |  |  |
| WithoutTraction | 44,954 | 37,770 | 38,755 |
| WithTraction | 617 | 340 | 365 |
| **Other** | 4,593 | 2,932 | 2,747 |
| **IfOtherMerged** |  |  |  |
| Abnormal foveal contour | 158 | 96 | 109 |
| AH | 89 | 46 | 52 |
| Angioid streaks | 1 | 1 | 1 |
| Chorioretinal changes outside macula | 47 | 24 | 25 |
| Chorioretinal changes within macula | 57 | 30 | 29 |
| Choroidal rupture | 8 | 0 | 8 |
| Coloboma | 4 | 0 | 4 |
| Congenital loop on disc | 10 | 7 | 4 |
| Cotton wool spot | 40 | 23 | 18 |
| CSCR | 228 | 125 | 144 |
| Dragged disc | 2 | 1 | 2 |
| Dragged macula | 1 | 1 | 0 |
| Embolus | 8 | 5 | 5 |
| Exudate | 28 | 14 | 15 |
| Fibroproliferation | 17 | 10 | 7 |
| Focal inflammatory changes | 17 | 9 | 13 |
| Hypoplastic disc | 6 | 4 | 5 |
| Idiopathic SPED | 100 | 54 | 56 |
| Inherited retinal disease | 41 | 41 | 40 |
| Inner retinal changes | 205 | 113 | 130 |
| MacTel | 5 | 5 | 5 |
| MD | 290 | 241 | 216 |
| MH | 312 | 174 | 168 |
| MO | 64 | 46 | 40 |
| Morning glory syndrome | 1 | 1 | 0 |
| Myelinated nerve fibres | 104 | 57 | 63 |
| Naev | 1,458 | 811 | 695 |
| Non-AMD neovascularisation | 2 | 2 | 0 |
| Optic disc drusen | 19 | 14 | 12 |
| Optic disc pallor | 5 | 3 | 3 |
| Optic disc pit | 8 | 2 | 6 |
| Outer retinal changes | 617 | 371 | 359 |
| Pigmented lesion outside macula | 132 | 74 | 59 |
| Pigmented lesion within macula | 124 | 75 | 55 |
| Pigmented spot outside macula | 101 | 48 | 56 |
| Pigmented spot within macula | 115 | 82 | 45 |
| RD | 34 | 23 | 12 |
| Retinal folds | 40 | 31 | 31 |
| Retinal hamartoma | 2 | 1 | 1 |
| Retinal ischaemia | 1 | 1 | 0 |
| SFCS | 46 | 24 | 27 |
| Swollen optic disc | 29 | 24 | 21 |
| Vascular changes | 61 | 39 | 39 |
| Vessel tortuosity | 163 | 147 | 137 |
| Vitelliform macular dystrophy | 73 | 57 | 54 |
| Vitreous changes | 35 | 22 | 18 |
