# Supplementary material for: UK Biobank retinal imaging grading: methodology, baseline characteristics and findings for common ocular diseases
Source: Eye (Lond). 2022 Nov 3;37(10):2109–16. doi: 10.1038/s41433-022-02298-7 (PMC10333328; doi:10.1038/s41433-022-02298-7)
Supplement: Supplementary file 1 [file 41433_2022_2298_MOESM5_ESM.docx]

**sFigure 1.** Numbers of participants who attended for retinal imaging with a diagnostic record of age-related macular degeneration, glaucoma and diabetic retinopathy identified through self-reported (verbal intervew), primary care, hospital episode statistics and death register records and those with suspicious features for these conditions identified from retinal imaging (i) at the time of imaging (ii) at any time. Abbreviations: AMD = age-related macular degeneration, DR = diabetic retinopathy, MA = microaneurysm.

**sFigure 2.** Comparisons of baseline measurements for LDL and HDL cholesterol, triglycerides and diastolic blood pressure between participants who were identified from retinal imaging to have suspected age-related macular degeneration, glaucoma, diabetic retinopathy or other vascular disease. Statistically significant pairwise comparisons (Student’s t-test) are indicated by asterisks: p<0.05 (*), p<0.01 (**), p<0.001(***). Abbreviations: LDL = low-density lipoprotein cholesterol; HDL = high-density lipoprotein cholesterol.

# Supplementary Material

**sTable 1.** Data dictionary for available variables in the retinal gradings dataset. Variables relating specifically to either colour fundus photographs or optical coherence tomography scans are prefixed with ‘COL_’ and ‘OCT_’ respectively. Those without a prefix are multimodal. Column ‘PersonEye’ indicates whether variables are recorded at either a person or eye level.

**sTable 2.** Clinical codes used to identify diagnosed ocular (age-related macular degeneration, diabetic retinopathy and glaucoma) and systemic disease (diabetes, hypertension) in the UK Biobank from self-reported (UK Biobank data coding 6) and linked primary care (Read 2 and Read 3), hospital episodes statistics and death register (ICD-9 and ICD-10) data.

**sTable 3.** Participant characteristics comparing individuals who had gradable retinal images (optical coherence tomography or colour photos) in both eyes with those where retinal images were either unavailable or ungradable in at least one eye. Characteristics were ascertained from self-reported data at study baseline and linked electronic health care records data (primary care, hospital episode statistics, death register) up until the time of retinal imaging, or 1st January 2010 for participants who did not undergo imaging. Continuous variables are summarised by mean (standard deviation) and compared using the Student’s t-test and One-Way ANOVA for two and more than two groups respectively. Categorical variables are summarised by number (percentage) and compared using Pearson’s Chi-squared test. Abbreviations: AMD = age-related macular degeneration, MA = microaneurysm.

**sTable 4.** Summary of all graded retinal features at the person level and by eye (see Supplementary Table 1 for variable definitions and abbreviations). VisitDate is summarised as a range, continuous variables by mean (standard deviation) and categorical variables by number. Person level summaries of categorical variables describe the number of individuals where at least one eye has that feature recorded.
