# Supplementary material for: UK Biobank retinal imaging grading: methodology, baseline characteristics and findings for common ocular diseases
Source: Eye (Lond). 2022 Nov 3;37(10):2109–16. doi: 10.1038/s41433-022-02298-7 (PMC10333328; doi:10.1038/s41433-022-02298-7)
Supplement: Supplementary file 1 [file 41433_2022_2298_MOESM1_ESM.pdf]

sTable 1. Data dictionary for available variables in the retinal gradings dataset. Variables relating specifically to either colour fundus photographs or optical coherence tomography scans are prefixed with 'COL_' and 'OCT_' respectively. Those without a prefix are multimodal. Column 'PersonEye' indicates whether variables are recorded at either a person or eye level.

Field	Notes	ValueType	PersonEye
VisitDate	The date of the visit; DD/MM/YYYY	Date	person
CupDiscRatioComparison	Absolute cup disc ratio comparison; 99 if CDR is missing for one or both eyes	Continuous	person
COL_Available	Are colour images available? Yes, No	Categorical single	eye
OCT_Available	Are optical coherence tomography (OCT) scans available? Yes, No	Categorical single	eye
COL_Quality	Colour image quality – Good, Fair, Poor or Cannot grade [CG]; NULL if COL_Available is No	Categorical single	eye
OCT_Quality	OCT scan quality – Good, Fair, Poor or Cannot grade [CG]; NULL if OCT_Available is No	Categorical single	eye
AbnormalitiesPresent	Are any abnormalities present? Yes, No; NULL only if all modalities are unavailable or ungradable	Categorical single	eye
AMD	Is there evidence of age-related macular degeneration (AMD)? Yes, No; NULL if AbnormalitiesPresent is No or NULL	Categorical single	eye
COL_IfAMD	On colour, is AMD: Exudative [Exud], Geographic atrophy [GA], Hyperpigmentation [Pigment], Drusen; NULL if AbnormalitiesPresent is No or NULL, colour images are unavailable or ungradable, or if AMD is not visible on colour images	Compound	eye
COL_DrusenInsideGrid	Are there drusen inside the grid? Yes, No; NULL if AbnormalitiesPresent is No or NULL, AMD is No or COL_IfAMD does not contain Drusen	Categorical single	eye
COL_SevereDrusen	Most severe drusen type in grid: <63µm [LessThan63], 63-125µm [SixtyThreeTo125], >125µm [GreaterThan125], Cannot grade [CG]; NULL if COL_DrusenInsideGrid is No or NULL	Categorical single	eye
COL_DrusenOutsideGrid	Are there drusen outside the grid? Yes, No; NULL if AbnormalitiesPresent is No, AMD is No or COL_IfAMD does not contain Drusen	Categorical single	eye

Field	Notes	ValueType	PersonEye
COL_ReticularDrusen	Reticular pseudodrusen? Yes, No, CG; NULL if AbnormalitiesPresent is No, AMD is No or COL_IfAMD does not contain Drusen	Categorical single	eye
OCT_DrusenPresent	On OCT, are drusen present? Yes, No; NULL if AbnormalitiesPresent is No, AMD is No or OCT images are unavailable or ungradable	Categorical single	eye
OCT_SubretDrusen	On OCT, are there subretinal drusenoid deposits? Yes, No; NULL if AbnormalitiesPresent is No, AMD is No or OCT images are unavailable or ungradable	Categorical single	eye
OCT_FocalAtrophy	On OCT, is there focal atrophy? Yes, No; NULL if AbnormalitiesPresent is No, AMD is No or OCT images are unavailable or ungradable	Categorical single	eye
OCT_CentralSubfieldThickness	Central subfield thickness; Integer obtained from automated OCT machine thickness map; NULL if OCT images are unavailable or ungradable	Integer	eye
COL_RetinalHaemorrhage	Is there evidence of retinal haemorrhage or microaneurysm? Yes, No; NULL if AbnormalitiesPresent is No or NULL if COL images are unavailable or ungradable	Categorical single	eye
COL_SignsOfDR	If COL_RetinalHaemorrhage is Yes, is it accompanied by characteristic signs of diabetic retinopathy? Yes, No; NULL if COL_RetinalHaemorrhage is No or NULL	Categorical single	eye
COL_DRFeatures	If COL_SignsOfDR is Yes, select all the characteristics that apply: Microaneurysm/haemorrhage [HMA], Venous loop [VL], Exudate [Ex], Cotton wool spots [CWS], Venous beading [VB], Venous reduplication [VR], Multiple blot haemorrhages [MBH], intra-retinal microvascular abnormalities [IRMA], Fibrous proliferation [FP], Laser scars [LS], New vessels on disc [NVD], New vessels elsewhere [NVE], Pre-retinal or vitreous haemorrhage [PVH], Pre-retinal fibrosis [PF], tractional retinal detachment [TRD]; NULL if COL_SignsOfDR is No or NULL	Compound	eye

Field	Notes	ValueType	PersonEye
COL_VisibleLaserTreatment	Any visible laser treatment? Yes, No; NULL if AbnormalitiesPresent is No or NULL; or COL images are unavailable or ungradable	Categorical single	eye
OCT_HyporeflectiveIntraretinalSpaces	On OCT, are there hyporeflective intraretinal spaces? Yes, No; NULL if AbnormalitiesPresent is No or NULL; or OCT images are unavailable or ungradable	Categorical single	eye
COL_CupDiscRatio	Cup disc ratio (CDR); Floating point measurement; NULL if CDR not gradable or COL images are unavailable	Continuous	eye
COL_HaemorrhageOnDisc	Haemorrhage on disc? Yes, No, Cannot grade [CG]; NULL if AbnormalitiesPresent is No or NULL	Categorical single	eye
COL_IfOpticDiscSuspicious	If suspicious: Notch, Inferior rim thinning [IRT], Notch and Inferior rim thinning [Both]; NULL if COL_CupDiscRatio is ≥0.7 or COL_HaemorrhageOnDisc is Yes	Categorical single	eye
Occlusion	Occlusion? Yes, No; NULL if AbnormalitiesPresent is No or NULL	Categorical single	eye
IfOcclusion	If Occlusion is Yes: Central retinal vein occlusion [CRVO], Branch retinal vein occlusion [BRVO], Central retinal artery occlusion [CRAO], Branch retinal artery occlusion [BRAO]; NULL if Occlusion is No or NULL	Categorical single	eye
EvidenceOfERM	Is there evidence of Epi-retinal membrane [ERM]? Yes, No; NULL if AbnormalitiesPresent is No or NULL	Categorical single	eye
IfERMStructuralChanges	Are there any retinal structural changes associated with the ERM? Yes, No; NULL if EvidenceOfERM is No or NULL	Categorical single	eye
OCT_EvidenceOfVMA	Is there evidence of vitreomacular adhesion [VMA]? Yes, No; NULL if AbnormalitiesPresent is No or NULL; or OCT images are unavailable or ungradable	Categorical single	eye
OCT_IfVMACategories	VMA categories: WithTraction, WithoutTraction; NULL if OCT_EvidenceOfVMA is No or NULL	Categorical single	eye

Field	Notes	ValueType	PersonEye
Other	Are other features present? Yes, No; NULL if AbnormalitiesPresent is No or NULL	Categorical single	eye
IfOther	If Other is Yes, select all that apply: Asteroid hyalosis [AH], Central serous chorio-retinopathy [CSCR], Macular hole (any stage) [MH], Macular telangiectasia [MacTel], Media opacity [MO], Myopic degeneration [MD], Naevus/Melanoma [Naev], Retinal detachment [RD], Schisis/foveal cystic changes [SFCS], Other; NULL if Other is No or NULL	Compound	eye
OtherComment	If Other was selected, please comment: Free-text; NULL if Other is No or NULL; NULL if IfOther does not contain Other	Compound	eye
IfOtherMerged	IfOther and OtherComment combined	Compound	eye

sTable 2. Clinical codes used to identify diagnosed ocular (age-related macular degeneration, diabetic retinopathy and glaucoma) and systemic disease (diabetes, hypertension) in the UK Biobank from self-reported (UK Biobank data coding 6) and linked primary care (Read 2 and Read 3), hospital episodes statistics and death register (ICD-9 and ICD-10) data.

Disease	Code	Code type	Description
Age-related macular degeneration	1528	Data coding 6	Macular degeneration
Age-related macular degeneration	H353	ICD-10	Degeneration of macula and posterior pole
Age-related macular degeneration	3625	ICD-9	DEGENERATION OF MACULA AND POSTERIOR POLE
Age-related macular degeneration	2BBH.	Read 2	Retinal drusen
Age-related macular degeneration	F425.	Read 2	Degeneration of macula and posterior pole
Age-related macular degeneration	F4250	Read 2	Unspecified senile macular degeneration
Age-related macular degeneration	F4251	Read 2	Dry senile macular degeneration
Age-related macular degeneration	F4252	Read 2	Wet senile macular degeneration
Age-related macular degeneration	F4257	Read 2	Drusen
Age-related macular degeneration	F425z	Read 2	Degeneration of macula or posterior pole NOS
Age-related macular degeneration	F4250	Read 3	Unspecified senile macular degeneration
Age-related macular degeneration	F4251	Read 3	Atrophic age-related macular degeneration
Age-related macular degeneration	F4252	Read 3	Subretinal neovascularisation of macula
Age-related macular degeneration	F4257	Read 3	Drusen
Age-related macular degeneration	F425z	Read 3	Degeneration of macula or posterior pole NOS
Age-related macular degeneration	X75mo	Read 3	Retinal drusen
Age-related macular degeneration	XE15x	Read 3	Degeneration of macula and posterior pole
Age-related macular degeneration	XE18j	Read 3	Age-related macular degeneration

Disease	Code	Code type	Description
Age-related macular degeneration	X75mp	Read 3	Macular drusen
Age-related macular degeneration	XaF41	Read 3	Drusen plus pigment change stage macular degeneration
Age-related macular degeneration	Xa9BN	Read 3	Macular degeneration
Diabetes	1220	Data coding 6	diabetes
Diabetes	1221	Data coding 6	gestational diabetes
Diabetes	1222	Data coding 6	type 1 diabetes
Diabetes	1223	Data coding 6	type 2 diabetes
Diabetes	E13	ICD-10	Other specified diabetes mellitus
Diabetes	E14	ICD-10	Unspecified diabetes mellitus
Diabetes	G590	ICD-10	Diabetic mononeuropathy
Diabetes	G632	ICD-10	Diabetic polyneuropathy
Diabetes	H280	ICD-10	Diabetic cataract
Diabetes	H360	ICD-10	Diabetic retinopathy
Diabetes	M142	ICD-10	Diabetic arthropathy
Diabetes	N083	ICD-10	Glomerular disorders in diabetes mellitus
Diabetes	O240	ICD-10	Diabetes mellitus in pregnancy: Pre-existing diabetes mellitus, insulin-dependent
Diabetes	O241	ICD-10	Diabetes mellitus in pregnancy: Pre-existing diabetes mellitus, non-insulin-dependent
Diabetes	O243	ICD-10	Diabetes mellitus in pregnancy: Pre-existing diabetes mellitus, unspecified
Diabetes	E130	ICD-10	Other specified diabetes mellitus With coma
Diabetes	E131	ICD-10	Other specified diabetes mellitus With ketoacidosis
Diabetes	E132	ICD-10	Other specified diabetes mellitus With renal complications
Diabetes	E133	ICD-10	Other specified diabetes mellitus With ophthalmic complications

Disease	Code	Code type	Description
Diabetes	E134	ICD-10	Other specified diabetes mellitus With neurological complications
Diabetes	E135	ICD-10	Other specified diabetes mellitus With peripheral circulatory complications
Diabetes	E136	ICD-10	Other specified diabetes mellitus With other specified complications
Diabetes	E137	ICD-10	Other specified diabetes mellitus With multiple complications
Diabetes	E138	ICD-10	Other specified diabetes mellitus With unspecified complications
Diabetes	E139	ICD-10	Other specified diabetes mellitus Without complications
Diabetes	E140	ICD-10	Unspecified diabetes mellitus With coma
Diabetes	E141	ICD-10	Unspecified diabetes mellitus With ketoacidosis
Diabetes	E142	ICD-10	Unspecified diabetes mellitus With renal complications
Diabetes	E143	ICD-10	Unspecified diabetes mellitus With ophthalmic complications
Diabetes	E144	ICD-10	Unspecified diabetes mellitus With neurological complications
Diabetes	E145	ICD-10	Unspecified diabetes mellitus With peripheral circulatory complications
Diabetes	E146	ICD-10	Unspecified diabetes mellitus With other specified complications
Diabetes	E147	ICD-10	Unspecified diabetes mellitus With multiple complications
Diabetes	E148	ICD-10	Unspecified diabetes mellitus With unspecified complications
Diabetes	E149	ICD-10	Unspecified diabetes mellitus Without complications
Diabetes	E10	ICD-10	Insulin-dependent diabetes mellitus
Diabetes	E100	ICD-10	Type 1 diabetes mellitus With coma
Diabetes	E101	ICD-10	Type 1 diabetes mellitus With ketoacidosis
Diabetes	E102	ICD-10	Type 1 diabetes mellitus With renal complications
Diabetes	E103	ICD-10	Type 1 diabetes mellitus With ophthalmic complications
Diabetes	E104	ICD-10	Type 1 diabetes mellitus With neurological complications
Diabetes	E105	ICD-10	Type 1 diabetes mellitus With peripheral circulatory complications
Diabetes	E106	ICD-10	Type 1 diabetes mellitus With other specified complications
Diabetes	E107	ICD-10	Type 1 diabetes mellitus With multiple complications

Disease	Code	Code type	Description
Diabetes	E108	ICD-10	Type 1 diabetes mellitus With unspecified complications
Diabetes	E109	ICD-10	Type 1 diabetes mellitus Without complications
Diabetes	E11	ICD-10	Non-insulin-dependent diabetes mellitus
Diabetes	E110	ICD-10	Type 2 diabetes mellitus With coma
Diabetes	E111	ICD-10	Type 2 diabetes mellitus With ketoacidosis
Diabetes	E112	ICD-10	Type 2 diabetes mellitus With renal complications
Diabetes	E113	ICD-10	Type 2 diabetes mellitus With ophthalmic complications
Diabetes	E114	ICD-10	Type 2 diabetes mellitus With neurological complications
Diabetes	E115	ICD-10	Type 2 diabetes mellitus With peripheral circulatory complications
Diabetes	E116	ICD-10	Type 2 diabetes mellitus With other specified complications
Diabetes	E117	ICD-10	Type 2 diabetes mellitus With multiple complications
Diabetes	E118	ICD-10	Type 2 diabetes mellitus With unspecified complications
Diabetes	E119	ICD-10	Type 2 diabetes mellitus Without complications
Diabetes	E12	ICD-10	Malnutrition-related diabetes mellitus
Diabetes	O242	ICD-10	Diabetes mellitus in pregnancy: Pre-existing malnutrition-related diabetes mellitus
Diabetes	E120	ICD-10	Malnutrition-related diabetes mellitus With coma
Diabetes	E121	ICD-10	Malnutrition-related diabetes mellitus With ketoacidosis
Diabetes	E122	ICD-10	Malnutrition-related diabetes mellitus With renal complications
Diabetes	E123	ICD-10	Malnutrition-related diabetes mellitus With ophthalmic complications
Diabetes	E124	ICD-10	Malnutrition-related diabetes mellitus With neurological complications
Diabetes	E125	ICD-10	Malnutrition-related diabetes mellitus With peripheral circulatory complications
Diabetes	E126	ICD-10	Malnutrition-related diabetes mellitus With other specified complications
Diabetes	E127	ICD-10	Malnutrition-related diabetes mellitus With multiple complications
Diabetes	E128	ICD-10	Malnutrition-related diabetes mellitus With unspecified complications

Disease	Code	Code type	Description
Diabetes	E129	ICD-10	Malnutrition-related diabetes mellitus Without complications
Diabetes	6480	ICD-9	DIABETES MELLITUS
Diabetes	C108.	Read 2	Type 1 diabetes mellitus
Diabetes	C1080	Read 2	Type I diabetes mellitus with renal complications
Diabetes	C1082	Read 2	Type I diabetes mellitus with neurological complications
Diabetes	C1084	Read 2	Unstable type I diabetes mellitus
Diabetes	C1085	Read 2	Type I diabetes mellitus with ulcer
Diabetes	C1087	Read 2	Type I diabetes mellitus with retinopathy
Diabetes	C1088	Read 2	Type I diabetes mellitus - poor control
Diabetes	C1089	Read 2	Type I diabetes mellitus maturity onset
Diabetes	C108A	Read 2	Type I diabetes mellitus without complication
Diabetes	C108D	Read 2	Type I diabetes mellitus with nephropathy
Diabetes	C108E	Read 2	Type I diabetes mellitus with hypoglycaemic coma
Diabetes	C108F	Read 2	Type I diabetes mellitus with diabetic cataract
Diabetes	C108H	Read 2	Type I diabetes mellitus with arthropathy
Diabetes	C108J	Read 2	Type I diabetes mellitus with neuropathic arthropathy
Diabetes	C10E.	Read 2	Type 1 diabetes mellitus
Diabetes	C10E0	Read 2	Type 1 diabetes mellitus with renal complications
Diabetes	C10E1	Read 2	Type 1 diabetes mellitus with ophthalmic complications
Diabetes	C10E2	Read 2	Type 1 diabetes mellitus with neurological complications
Diabetes	C10E3	Read 2	Type 1 diabetes mellitus with multiple complications
Diabetes	C10E4	Read 2	Unstable type 1 diabetes mellitus
Diabetes	C10E5	Read 2	Type 1 diabetes mellitus with ulcer
Diabetes	C10E6	Read 2	Type 1 diabetes mellitus with gangrene
Diabetes	C10E7	Read 2	Type 1 diabetes mellitus with retinopathy
Diabetes	C10E8	Read 2	Type 1 diabetes mellitus - poor control
Diabetes	C10E9	Read 2	Type 1 diabetes mellitus maturity onset
Diabetes	C10EA	Read 2	Type 1 diabetes mellitus without complication
Diabetes	C10EB	Read 2	Type 1 diabetes mellitus with mononeuropathy

Disease	Code	Code type	Description
Diabetes	C10EC	Read 2	Type 1 diabetes mellitus with polyneuropathy
Diabetes	C10ED	Read 2	Type 1 diabetes mellitus with nephropathy
Diabetes	C10EE	Read 2	Type 1 diabetes mellitus with hypoglycaemic coma
Diabetes	C10EF	Read 2	Type 1 diabetes mellitus with diabetic cataract
Diabetes	C10EG	Read 2	Type 1 diabetes mellitus with peripheral angiopathy
Diabetes	C10EH	Read 2	Type 1 diabetes mellitus with arthropathy
Diabetes	C10EJ	Read 2	Type 1 diabetes mellitus with neuropathic arthropathy
Diabetes	C10EK	Read 2	Type 1 diabetes mellitus with persistent proteinuria
Diabetes	C10EL	Read 2	Type 1 diabetes mellitus with persistent microalbuminuria
Diabetes	C10EM	Read 2	Type 1 diabetes mellitus with ketoacidosis
Diabetes	C10EN	Read 2	Type 1 diabetes mellitus with ketoacidotic coma
Diabetes	C10EP	Read 2	Type 1 diabetes mellitus with exudative maculopathy
Diabetes	C10EQ	Read 2	Type 1 diabetes mellitus with gastroparesis
Diabetes	C109.	Read 2	Type 2 diabetes mellitus
Diabetes	C1090	Read 2	Type II diabetes mellitus with renal complications
Diabetes	C1091	Read 2	Type II diabetes mellitus with ophthalmic complications
Diabetes	C1092	Read 2	Type II diabetes mellitus with neurological complications
Diabetes	C1094	Read 2	Type II diabetes mellitus with ulcer
Diabetes	C1095	Read 2	Type II diabetes mellitus with gangrene
Diabetes	C1096	Read 2	Type II diabetes mellitus with retinopathy
Diabetes	C1097	Read 2	Type II diabetes mellitus - poor control
Diabetes	C109A	Read 2	Type II diabetes mellitus with mononeuropathy
Diabetes	C109B	Read 2	Type II diabetes mellitus with polyneuropathy
Diabetes	C109C	Read 2	Type II diabetes mellitus with nephropathy
Diabetes	C109D	Read 2	Type II diabetes mellitus with hypoglycaemic coma
Diabetes	C109E	Read 2	Type II diabetes mellitus with diabetic cataract
Diabetes	C109F	Read 2	Type II diabetes mellitus with peripheral angiopathy
Diabetes	C109G	Read 2	Type II diabetes mellitus with arthropathy
Diabetes	C109H	Read 2	Type II diabetes mellitus with neuropathic arthropathy

Disease	Code	Code type	Description
Diabetes	C109J	Read 2	Insulin treated Type 2 diabetes mellitus
Diabetes	C109K	Read 2	Hyperosmolar non-ketotic state in type 2 diabetes mellitus
Diabetes	C10F.	Read 2	Type 2 diabetes mellitus
Diabetes	C10F0	Read 2	Type 2 diabetes mellitus with renal complications
Diabetes	C10F1	Read 2	Type 2 diabetes mellitus with ophthalmic complications
Diabetes	C10F2	Read 2	Type 2 diabetes mellitus with neurological complications
Diabetes	C10F3	Read 2	Type 2 diabetes mellitus with multiple complications
Diabetes	C10F4	Read 2	Type 2 diabetes mellitus with ulcer
Diabetes	C10F5	Read 2	Type 2 diabetes mellitus with gangrene
Diabetes	C10F6	Read 2	Type 2 diabetes mellitus with retinopathy
Diabetes	C10F7	Read 2	Type 2 diabetes mellitus - poor control
Diabetes	C10F9	Read 2	Type 2 diabetes mellitus without complication
Diabetes	C10FA	Read 2	Type 2 diabetes mellitus with mononeuropathy
Diabetes	C10FB	Read 2	Type 2 diabetes mellitus with polyneuropathy
Diabetes	C10FC	Read 2	Type 2 diabetes mellitus with nephropathy
Diabetes	C10FD	Read 2	Type 2 diabetes mellitus with hypoglycaemic coma
Diabetes	C10FE	Read 2	Type 2 diabetes mellitus with diabetic cataract
Diabetes	C10FF	Read 2	Type 2 diabetes mellitus with peripheral angiopathy
Diabetes	C10FG	Read 2	Type 2 diabetes mellitus with arthropathy
Diabetes	C10FH	Read 2	Type 2 diabetes mellitus with neuropathic arthropathy
Diabetes	C10FJ	Read 2	Insulin treated Type 2 diabetes mellitus
Diabetes	C10FK	Read 2	Hyperosmolar non-ketotic state in type 2 diabetes mellitus
Diabetes	C10FL	Read 2	Type 2 diabetes mellitus with persistent proteinuria
Diabetes	C10FM	Read 2	Type 2 diabetes mellitus with persistent microalbuminuria
Diabetes	C10FN	Read 2	Type 2 diabetes mellitus with ketoacidosis
Diabetes	C10FP	Read 2	Type 2 diabetes mellitus with ketoacidotic coma
Diabetes	C10FQ	Read 2	Type 2 diabetes mellitus with exudative maculopathy
Diabetes	C10FR	Read 2	Type 2 diabetes mellitus with gastroparesis
Diabetes	X40J4	Read 3	Type I diabetes mellitus

Disease	Code	Code type	Description
Diabetes	C1080	Read 3	Type I diabetes mellitus with renal complications
Diabetes	C1082	Read 3	Type I diabetes mellitus with neurological complications
Diabetes	Xa4g7	Read 3	Unstable type I diabetes mellitus
Diabetes	C1085	Read 3	Type I diabetes mellitus with ulcer
Diabetes	C1087	Read 3	Type I diabetes mellitus with retinopathy
Diabetes	C1088	Read 3	Type I diabetes mellitus - poor control
Diabetes	C1089	Read 3	Type I diabetes mellitus maturity onset
Diabetes	XaELP	Read 3	Type I diabetes mellitus without complication
Diabetes	XaF04	Read 3	Type I diabetes mellitus with nephropathy
Diabetes	XaFWG	Read 3	Type I diabetes mellitus with hypoglycaemic coma
Diabetes	XaFm8	Read 3	Type I diabetes mellitus with diabetic cataract
Diabetes	XaFmL	Read 3	Type I diabetes mellitus with arthropathy
Diabetes	XaFmM	Read 3	Type I diabetes mellitus with neuropathic arthropathy
Diabetes	C1081	Read 3	Type I diabetes mellitus with ophthalmic complications
Diabetes	C1083	Read 3	Type I diabetes mellitus with multiple complications
Diabetes	C1086	Read 3	Type I diabetes mellitus with gangrene
Diabetes	XaEnn	Read 3	Type I diabetes mellitus with mononeuropathy
Diabetes	XaEno	Read 3	Type I diabetes mellitus with polyneuropathy
Diabetes	XaFmK	Read 3	Type I diabetes mellitus with peripheral angiopathy
Diabetes	XaIzM	Read 3	Type 1 diabetes mellitus with persistent proteinuria
Diabetes	XaIzN	Read 3	Type 1 diabetes mellitus with persistent microalbuminuria
Diabetes	C1010	Read 3	Type 1 diabetes mellitus with ketoacidosis
Diabetes	C1030	Read 3	Type 1 diabetes mellitus with ketoacidotic coma
Diabetes	XaJSr	Read 3	Type 1 diabetes mellitus with exudative maculopathy
Diabetes	XaKyW	Read 3	Type 1 diabetes mellitus with gastroparesis
Diabetes	X40J5	Read 3	Type II diabetes mellitus
Diabetes	C1090	Read 3	Type II diabetes mellitus with renal complications
Diabetes	C1091	Read 3	Type II diabetes mellitus with ophthalmic complications
Diabetes	C1092	Read 3	Type II diabetes mellitus with neurological complications

Disease	Code	Code type	Description
Diabetes	C1094	Read 3	Type II diabetes mellitus with ulcer
Diabetes	C1095	Read 3	Type II diabetes mellitus with gangrene
Diabetes	C1096	Read 3	Type II diabetes mellitus with retinopathy
Diabetes	C1097	Read 3	Type II diabetes mellitus - poor control
Diabetes	XaEnp	Read 3	Type II diabetes mellitus with mononeuropathy
Diabetes	XaEnq	Read 3	Type II diabetes mellitus with polyneuropathy
Diabetes	XaF05	Read 3	Type II diabetes mellitus with nephropathy
Diabetes	XaFWI	Read 3	Type II diabetes mellitus with hypoglycaemic coma
Diabetes	XaFmA	Read 3	Type II diabetes mellitus with diabetic cataract
Diabetes	XaFn7	Read 3	Type II diabetes mellitus with peripheral angiopathy
Diabetes	XaFn8	Read 3	Type II diabetes mellitus with arthropathy
Diabetes	XaFn9	Read 3	Type II diabetes mellitus with neuropathic arthropathy
Diabetes	X40J6	Read 3	Insulin treated Type 2 diabetes mellitus
Diabetes	XaIrf	Read 3	Hyperosmolar non-ketotic state in type II diabetes mellitus
Diabetes	C1093	Read 3	Type II diabetes mellitus with multiple complications
Diabetes	XaELQ	Read 3	Type II diabetes mellitus without complication
Diabetes	XaIzQ	Read 3	Type II diabetes mellitus with persistent proteinuria
Diabetes	XaIzR	Read 3	Type II diabetes mellitus with persistent microalbuminuria
Diabetes	C1011	Read 3	Type 2 diabetes mellitus with ketoacidosis
Diabetes	C1031	Read 3	Type II diabetes mellitus with ketoacidotic coma
Diabetes	XaJQp	Read 3	Type II diabetes mellitus with exudative maculopathy
Diabetes	XaKyX	Read 3	Type II diabetes mellitus with gastroparesis
Diabetic retinopathy	1276	Data coding 6	diabetic eye disease
Diabetic retinopathy	E103	ICD-10	Type 1 diabetes mellitus With ophthalmic complications
Diabetic retinopathy	E113	ICD-10	Type 2 diabetes mellitus With ophthalmic complications
Diabetic retinopathy	E123	ICD-10	Malnutrition-related diabetes mellitus With ophthalmic complications
Diabetic retinopathy	E133	ICD-10	Other specified diabetes mellitus With ophthalmic complications

Disease	Code	Code type	Description
Diabetic retinopathy	E143	ICD-10	Unspecified diabetes mellitus With ophthalmic complications
Diabetic retinopathy	H360	ICD-10	Diabetic retinopathy
Diabetic retinopathy	2504	ICD-9	DIABETES WITH OPHTHALMIC MANIFESTATIONS
Diabetic retinopathy	3620A	ICD-9	DIABETIC RETINOPATHY
Diabetic retinopathy	2BBF.	Read 2	Retinal abnormality - diabetes related
Diabetic retinopathy	C105.	Read 2	Diabetes mellitus with ophthalmic manifestation
Diabetic retinopathy	C1050	Read 2	Diabetes mellitus, juvenile type, with ophthalmic manifestation
Diabetic retinopathy	C1051	Read 2	Diabetes mellitus, adult onset, with ophthalmic manifestation
Diabetic retinopathy	C105y	Read 2	Other specified diabetes mellitus with ophthalmic complications
Diabetic retinopathy	C105z	Read 2	Diabetes mellitus NOS with ophthalmic manifestation
Diabetic retinopathy	C1081	Read 2	Insulin-dependent diabetes mellitus with ophthalmic complications
Diabetic retinopathy	C1087	Read 2	Insulin dependent diabetes mellitus with retinopathy
Diabetic retinopathy	C1091	Read 2	Non-insulin-dependent diabetes mellitus with ophthalmic complications
Diabetic retinopathy	C1096	Read 2	Non-insulin-dependent diabetes mellitus with retinopathy
Diabetic retinopathy	C10A3	Read 2	Malnutrition-related diabetes mellitus with ophthalmic complications
Diabetic retinopathy	C10E1	Read 2	Type 1 diabetes mellitus with ophthalmic complications
Diabetic retinopathy	C10E7	Read 2	Type 1 diabetes mellitus with retinopathy
Diabetic retinopathy	C10F1	Read 2	Type 2 diabetes mellitus with ophthalmic complications
Diabetic retinopathy	C10F6	Read 2	Type 2 diabetes mellitus with retinopathy
Diabetic retinopathy	F420.	Read 2	Diabetic retinopathy
Diabetic retinopathy	F420z	Read 2	Diabetic retinopathy NOS
Diabetic retinopathy	2BBP.	Read 2	O/E - right eye background diabetic retinopathy
Diabetic retinopathy	2BBQ.	Read 2	O/E - left eye background diabetic retinopathy
Diabetic retinopathy	F4200	Read 2	Background diabetic retinopathy
Diabetic retinopathy	F4206	Read 2	Non proliferative diabetic retinopathy
Diabetic retinopathy	2BBo.	Read 2	O/E - sight threatening diabetic retinopathy

Disease	Code	Code type	Description
Diabetic retinopathy	2BBR.	Read 2	O/E - right eye preproliferative diabetic retinopathy
Diabetic retinopathy	2BBS.	Read 2	O/E - left eye preproliferative diabetic retinopathy
Diabetic retinopathy	F4202	Read 2	Preproliferative diabetic retinopathy
Diabetic retinopathy	F4208	Read 2	High risk non proliferative diabetic retinopathy
Diabetic retinopathy	2BBr.	Read 2	Impaired vision due to diabetic retinopathy
Diabetic retinopathy	2BBk.	Read 2	O/E - right eye stable treated proliferative diabetic retinopathy
Diabetic retinopathy	2BBl.	Read 2	O/E - left eye stable treated proliferative diabetic retinopathy
Diabetic retinopathy	2BBT.	Read 2	O/E - right eye proliferative diabetic retinopathy
Diabetic retinopathy	2BBV.	Read 2	O/E - left eye proliferative diabetic retinopathy
Diabetic retinopathy	7276.	Read 2	Pan retinal photocoagulation for diabetes
Diabetic retinopathy	F4201	Read 2	Proliferative diabetic retinopathy
Diabetic retinopathy	F4205	Read 2	Advanced diabetic retinal disease
Diabetic retinopathy	F4207	Read 2	High risk proliferative diabetic retinopathy
Diabetic retinopathy	2BBm.	Read 2	O/E - right eye clinically significant macular oedema
Diabetic retinopathy	2BBn.	Read 2	O/E - left eye clinically significant macular oedema
Diabetic retinopathy	C10EP	Read 2	Type 1 diabetes mellitus with exudative maculopathy
Diabetic retinopathy	C10FQ	Read 2	Type 2 diabetes mellitus with exudative maculopathy
Diabetic retinopathy	F4203	Read 2	Advanced diabetic maculopathy
Diabetic retinopathy	2BBL.	Read 2	O/E - diabetic maculopathy present both eyes
Diabetic retinopathy	2BBW.	Read 2	O/E - right eye diabetic maculopathy
Diabetic retinopathy	2BBX.	Read 2	O/E - left eye diabetic maculopathy
Diabetic retinopathy	F4204	Read 2	Diabetic maculopathy
Diabetic retinopathy	C105.	Read 3	Diabetes mellitus with ophthalmic manifestation
Diabetic retinopathy	C1050	Read 3	Diabetes mellitus, juvenile type, with ophthalmic manifestation
Diabetic retinopathy	C1051	Read 3	Diabetes mellitus, adult onset, with ophthalmic manifestation
Diabetic retinopathy	C105y	Read 3	Other specified diabetes mellitus with ophthalmic complications
Diabetic retinopathy	C105z	Read 3	Diabetes mellitus NOS with ophthalmic manifestation

Disease	Code	Code type	Description
Diabetic retinopathy	C1081	Read 3	Type I diabetes mellitus with ophthalmic complications
Diabetic retinopathy	C1087	Read 3	Type I diabetes mellitus with retinopathy
Diabetic retinopathy	C1091	Read 3	Type II diabetes mellitus with ophthalmic complications
Diabetic retinopathy	C1096	Read 3	Type II diabetes mellitus with retinopathy
Diabetic retinopathy	C10A3	Read 3	Malnutrition-related diabetes mellitus with ophthalmic complications
Diabetic retinopathy	F420.	Read 3	Diabetic retinopathy
Diabetic retinopathy	F420z	Read 3	Diabetic retinopathy NOS
Diabetic retinopathy	XE12G	Read 3	Diabetes + eye manifestation (& [cataract] or [retinopathy])
Diabetic retinopathy	XaBuI	Read 3	Retinal abnormality - diabetes-related
Diabetic retinopathy	XaXfs	Read 3	Diabetic retinopathy detected by national screening programme
Diabetic retinopathy	F4200	Read 3	Background diabetic retinopathy
Diabetic retinopathy	XaE5T	Read 3	Mild non proliferative diabetic retinopathy
Diabetic retinopathy	XaIP5	Read 3	Non proliferative diabetic retinopathy
Diabetic retinopathy	XaJOg	Read 3	O/E - right eye background diabetic retinopathy
Diabetic retinopathy	XaJOh	Read 3	O/E - left eye background diabetic retinopathy
Diabetic retinopathy	F4202	Read 3	Preproliferative diabetic retinopathy
Diabetic retinopathy	X00dF	Read 3	Visually threatening diabetic retinopathy
Diabetic retinopathy	XaE5U	Read 3	Moderate non proliferative diabetic retinopathy
Diabetic retinopathy	XaE5V	Read 3	Severe non proliferative diabetic retinopathy
Diabetic retinopathy	XaIW8	Read 3	High risk non proliferative diabetic retinopathy
Diabetic retinopathy	XaJOi	Read 3	O/E - right eye preproliferative diabetic retinopathy
Diabetic retinopathy	XaJOj	Read 3	O/E - left eye preproliferative diabetic retinopathy
Diabetic retinopathy	XaKcS	Read 3	O/E - sight threatening diabetic retinopathy
Diabetic retinopathy	XaPen	Read 3	Impaired vision due to diabetic retinopathy
Diabetic retinopathy	F4201	Read 3	Proliferative diabetic retinopathy
Diabetic retinopathy	X00dH	Read 3	Proliferative diabetic retinopathy new vessels on disc
Diabetic retinopathy	X00dI	Read 3	Proliferative diabetic retinopathy with new vessels elsewhere than on disc

Disease	Code	Code type	Description
Diabetic retinopathy	X00dJ	Read 3	Diabetic traction retinal detachment
Diabetic retinopathy	XaD2T	Read 3	Advanced diabetic retinal disease
Diabetic retinopathy	XaE5W	Read 3	Proliferative diabetic retinopathy - non high risk
Diabetic retinopathy	XaE5X	Read 3	Proliferative diabetic retinopathy - high risk
Diabetic retinopathy	XaE5Y	Read 3	Proliferative diabetic retinopathy - quiescent
Diabetic retinopathy	XaE5Z	Read 3	Proliferative diabetic retinopathy - iris neovascularisation
Diabetic retinopathy	XaJOk	Read 3	O/E - right eye proliferative diabetic retinopathy
Diabetic retinopathy	XaJOl	Read 3	O/E - left eye proliferative diabetic retinopathy
Diabetic retinopathy	XaJlI	Read 3	Pan retinal photocoagulation for diabetes
Diabetic retinopathy	XaKDG	Read 3	O/E - right eye stable treated proliferative diabetic retinopathy
Diabetic retinopathy	XaKDH	Read 3	O/E - left eye stable treated proliferative diabetic retinopathy
Diabetic retinopathy	F4203	Read 3	Advanced diabetic maculopathy
Diabetic retinopathy	XaE5b	Read 3	Clinically significant macular oedema
Diabetic retinopathy	XaE5c	Read 3	Diabetic macular oedema
Diabetic retinopathy	XaEVO	Read 3	Diffuse diabetic maculopathy
Diabetic retinopathy	XaEVP	Read 3	Focal diabetic maculopathy
Diabetic retinopathy	XaEVS	Read 3	Ischaemic diabetic maculopathy
Diabetic retinopathy	XaEVT	Read 3	Mixed diabetic maculopathy
Diabetic retinopathy	XaJQp	Read 3	Type II diabetes mellitus with exudative maculopathy
Diabetic retinopathy	XaJSr	Read 3	Type 1 diabetes mellitus with exudative maculopathy
Diabetic retinopathy	XaKDI	Read 3	O/E - right eye clinically significant macular oedema
Diabetic retinopathy	XaKDJ	Read 3	O/E - left eye clinically significant macular oedema
Diabetic retinopathy	X00dG	Read 3	Diabetic maculopathy
Diabetic retinopathy	XaIPk	Read 3	O/E - diabetic maculopathy present both eyes
Diabetic retinopathy	XaJOn	Read 3	O/E - right eye diabetic maculopathy
Diabetic retinopathy	XaJOo	Read 3	O/E - left eye diabetic maculopathy
Glaucoma	1436	Data coding 5	glaucoma surgery/trabeculectomy

Disease	Code	Code type	Description
Glaucoma	1277	Data coding 6	glaucoma
Glaucoma	Q150	ICD-10	Congenital glaucoma
Glaucoma	H40	ICD-10	Glaucoma
Glaucoma	H409	ICD-10	Glaucoma, unspecified
Glaucoma	H402	ICD-10	Primary angle-closure glaucoma
Glaucoma	H401	ICD-10	Primary open-angle glaucoma
Glaucoma	H403	ICD-10	Glaucoma secondary to eye trauma
Glaucoma	H404	ICD-10	Glaucoma secondary to eye inflammation
Glaucoma	H405	ICD-10	Glaucoma secondary to other eye disorders
Glaucoma	H406	ICD-10	Glaucoma secondary to drugs
Glaucoma	H408	ICD-10	Other glaucoma
Glaucoma	H42	ICD-10	Glaucoma in diseases classified elsewhere
Glaucoma	H420	ICD-10	Glaucoma in endocrine, nutritional and metabolic diseases
Glaucoma	H428	ICD-10	Glaucoma in other diseases classified elsewhere
Glaucoma	365	ICD-9	GLAUCOMA
Glaucoma	3659	ICD-9	UNSPECIFIED
Glaucoma	V801	ICD-9	GLAUCOMA
Glaucoma	3652	ICD-9	PRIMARY ANGLE-CLOSURE GLAUCOMA
Glaucoma	3653	ICD-9	CORTICOSTEROID-INDUCED GLAUCOMA
Glaucoma	3654	ICD-9	GLAUCOMA ASSOCIATED WITH CONGENITAL ANOMALIES, WIT
Glaucoma	3654A	ICD-9	GLAUCOMA ASSOCIATED WITH CONGENITAL ANOMALIES, WITH DYSTROPH
Glaucoma	3655	ICD-9	GLAUCOMA ASSOCIATED WITH DISORDERS OF THE LENS
Glaucoma	3656	ICD-9	GLAUCOMA ASSOCIATED WITH OTHER OCULAR DISORDERS
Glaucoma	3658	ICD-9	OTHER GLAUCOMA
Glaucoma	3651	ICD-9	OPEN-ANGLE GLAUCOMA
Glaucoma	C601	OPCS-4	C60.1 Trabeculectomy

Disease	Code	Code type	Description
Glaucoma	C605	OPCS-4	C60.5 Insertion of tube into anterior chamber of eye to assist drainage of aqueous humour
Glaucoma	C606	OPCS-4	C60.6 Viscocanulostomy
Glaucoma	C61	OPCS-4	C61 Other operations on trabecular meshwork of eye
Glaucoma	C611	OPCS-4	C61.1 Laser trabeculoplasty
Glaucoma	C612	OPCS-4	C61.2 Trabeculotomy
Glaucoma	C613	OPCS-4	C61.3 Goniotomy
Glaucoma	C614	OPCS-4	C61.4 Goniopuncture
Glaucoma	C615	OPCS-4	C61.5 Viscogonioplasty
Glaucoma	C618	OPCS-4	C61.8 Other specified other operations on trabecular meshwork of eye
Glaucoma	C619	OPCS-4	C61.9 Unspecified other operations on trabecular meshwork of eye
Glaucoma	C65	OPCS-4	C65 Operations following glaucoma surgery
Glaucoma	C654	OPCS-4	C65.4 Removal of releasable suture following glaucoma surgery
Glaucoma	C655	OPCS-4	C65.5 Laser suture lysis following glaucoma surgery
Glaucoma	C658	OPCS-4	C65.8 Other specified operations following glaucoma surgery
Glaucoma	C659	OPCS-4	C65.9 Unspecified operations following glaucoma surgery
Glaucoma	C663	OPCS-4	C66.3 Cryotherapy to ciliary body
Glaucoma	C664	OPCS-4	C66.4 Laser photocoagulation of ciliary body
Glaucoma	C665	OPCS-4	C66.5 Destruction of ciliary body NEC
Glaucoma	F4514	Read 2	Glaucoma of childhood
Glaucoma	P3200	Read 2	Congenital glaucoma
Glaucoma	Q20y7	Read 2	Traumatic glaucoma due to birth trauma
Glaucoma	72550	Read 2	Trabeculectomy
Glaucoma	72554	Read 2	Insertion of tube into anterior chamber of eye to assist drainage of aqueous humour
Glaucoma	72555	Read 2	Viscocanulostomy
Glaucoma	72556	Read 2	Revision of trabeculectomy
Glaucoma	7256.	Read 2	Other operations on trabecular meshwork of eye

Disease	Code	Code type	Description
Glaucoma	72560	Read 2	Laser trabeculoplasty
Glaucoma	72561	Read 2	Trabeculotomy
Glaucoma	72562	Read 2	Goniotomy
Glaucoma	72563	Read 2	Goniopuncture
Glaucoma	72564	Read 2	Viscogonioplasty
Glaucoma	7256y	Read 2	Other specified other operation on trabecular meshwork of eye
Glaucoma	7256z	Read 2	Other operation on trabecular meshwork of eye NOS
Glaucoma	72587	Read 2	Resuturing of trabeculectomy
Glaucoma	7259.	Read 2	Operations following glaucoma surgery
Glaucoma	72590	Read 2	Needling of bleb following glaucoma surgery
Glaucoma	72591	Read 2	Injection of bleb following glaucoma surgery
Glaucoma	72592	Read 2	Revision of bleb NEC following glaucoma surgery
Glaucoma	72593	Read 2	Removal of releasable suture following glaucoma surgery
Glaucoma	72594	Read 2	Laser suture lysis following glaucoma surgery
Glaucoma	7259y	Read 2	Other specified operations following glaucoma surgery
Glaucoma	7259z	Read 2	Operations following glaucoma surgery NOS
Glaucoma	72601	Read 2	Cryotherapy to ciliary body
Glaucoma	72603	Read 2	Laser photocoagulation of ciliary body
Glaucoma	72604	Read 2	Destruction of ciliary body NEC
Glaucoma	7275.	Read 2	Pan retinal photocoagulation for glaucoma
Glaucoma	F45..	Read 2	Glaucoma
Glaucoma	F45z.	Read 2	Glaucoma NOS
Glaucoma	F4H14	Read 2	Optic disc glaucomatous atrophy
Glaucoma	FyuG.	Read 2	[X]Glaucoma
Glaucoma	1482.	Read 2	H/O: glaucoma
Glaucoma	F452.	Read 2	Primary angle-closure glaucoma
Glaucoma	F4520	Read 2	Unspecified primary angle-closure glaucoma
Glaucoma	F4521	Read 2	Intermittent primary angle-closure glaucoma

Disease	Code	Code type	Description
Glaucoma	F4522	Read 2	Acute primary angle-closure glaucoma
Glaucoma	F4523	Read 2	Chronic primary angle-closure glaucoma
Glaucoma	F4524	Read 2	Primary angle-closure glaucoma residual stage
Glaucoma	F452z	Read 2	Primary angle-closure glaucoma NOS
Glaucoma	F4511	Read 2	Primary open-angle glaucoma
Glaucoma	F4512	Read 2	Low tension glaucoma
Glaucoma	F45y2	Read 2	Low tension glaucoma
Glaucoma	F4042	Read 2	Blind hypertensive eye
Glaucoma	F4421	Read 2	Glaucomatocyclitic crises
Glaucoma	F4513	Read 2	Pigmentary glaucoma
Glaucoma	F453.	Read 2	Steroid-induced glaucoma
Glaucoma	F4530	Read 2	Steroid-induced glaucoma glaucomatous stage
Glaucoma	F4531	Read 2	Steroid-induced glaucoma residual stage
Glaucoma	F453z	Read 2	Steroid-induced glaucoma NOS
Glaucoma	F454.	Read 2	Glaucoma due to disease EC
Glaucoma	F4540	Read 2	Glaucoma due to chamber angle anomaly
Glaucoma	F4541	Read 2	Glaucoma due to iris anomaly
Glaucoma	F4542	Read 2	Glaucoma due to other anterior segment anomaly
Glaucoma	F4543	Read 2	Glaucoma due to systemic syndrome
Glaucoma	F4544	Read 2	Glaucoma in endocrine, nutritional and metabolic diseases
Glaucoma	F454z	Read 2	Glaucoma due to disease NOS
Glaucoma	F455.	Read 2	Glaucoma associated with disorders of the lens
Glaucoma	F4550	Read 2	Phacolytic glaucoma
Glaucoma	F4551	Read 2	Pseudoexfoliation glaucoma
Glaucoma	F455z	Read 2	Glaucoma associated with disorders of the lens NOS
Glaucoma	F456.	Read 2	Glaucoma associated with other ocular disorders
Glaucoma	F4560	Read 2	Glaucoma due to unspecified ocular disorder
Glaucoma	F4561	Read 2	Glaucoma due to pupillary block
Glaucoma	F4562	Read 2	Glaucoma due to ocular inflammation

Disease	Code	Code type	Description
Glaucoma	F4563	Read 2	Glaucoma due to ocular vascular disorder
Glaucoma	F4564	Read 2	Glaucoma due to ocular tumour or cyst
Glaucoma	F4565	Read 2	Glaucoma due to ocular trauma
Glaucoma	F4566	Read 2	Neovascular glaucoma
Glaucoma	F456z	Read 2	Glaucoma associated with other ocular disorders NOS
Glaucoma	F45y.	Read 2	Other specified forms of glaucoma
Glaucoma	F45y0	Read 2	Hypersecretion glaucoma
Glaucoma	F45y1	Read 2	Glaucoma due to episode of increased venous pressure
Glaucoma	F45yz	Read 2	Other specified glaucoma NOS
Glaucoma	FyuG0	Read 2	[X]Other glaucoma
Glaucoma	FyuG1	Read 2	[X]Glaucoma in endocrine, nutritional and metabolic diseases classified elsewhere
Glaucoma	FyuG2	Read 2	[X]Glaucoma in other diseases classified elsewhere
Glaucoma	F4631	Read 2	Glaucomatous subcapsular flecks
Glaucoma	F4501	Read 2	Open angle glaucoma with borderline intraocular pressure
Glaucoma	F451.	Read 2	Open-angle glaucoma
Glaucoma	F4510	Read 2	Unspecified open-angle glaucoma
Glaucoma	F4515	Read 2	Open-angle glaucoma residual stage
Glaucoma	F451z	Read 2	Open-angle glaucoma NOS
Glaucoma	F4514	Read 3	Glaucoma of childhood
Glaucoma	P32..	Read 3	Congenital glaucoma
Glaucoma	P3421	Read 3	Irido-corneo-trabecular dysgenesis
Glaucoma	P3422	Read 3	Irido-trabecular dysgenesis
Glaucoma	Q20y7	Read 3	Traumatic glaucoma due to birth trauma
Glaucoma	X75kE	Read 3	Goniodysgenesis iris ciliary body abnormal
Glaucoma	X77NV	Read 3	Goniodysgenesis
Glaucoma	X77t4	Read 3	Trabecular dysgenesis
Glaucoma	.N141	Read 3	Congenital glaucoma
Glaucoma	P3200	Read 3	Congenital glaucoma

Disease	Code	Code type	Description
Glaucoma	72550	Read 3	Trabeculectomy
Glaucoma	72554	Read 3	Insertion anterior chamber drainage tube (& Molteno tube)
Glaucoma	7256.	Read 3	Other operations on trabecular meshwork of eye
Glaucoma	72560	Read 3	Laser trabeculoplasty
Glaucoma	72561	Read 3	Trabeculotomy
Glaucoma	72562	Read 3	Goniotomy (& Barkan)
Glaucoma	72563	Read 3	Goniopuncture (& Barkan)
Glaucoma	7256y	Read 3	Other specified other operation on trabecular meshwork of eye
Glaucoma	7256z	Read 3	Other operation on trabecular meshwork of eye NOS
Glaucoma	7260.	Read 3	Destruction of ciliary body
Glaucoma	72603	Read 3	Laser coagulation of ciliary body
Glaucoma	72604	Read 3	Destruction of ciliary body NEC
Glaucoma	7275.	Read 3	Panretinal photocoagulation for glaucoma
Glaucoma	X00Wr	Read 3	Insertion of Molteno tube into anterior chamber
Glaucoma	X00X4	Read 3	Trabecular meshwork operation
Glaucoma	X00X5	Read 3	Trabeculoplasty
Glaucoma	X00X6	Read 3	Surgical trabeculoplasty
Glaucoma	X00X8	Read 3	Ultrasound destruction of ciliary body
Glaucoma	X00XI	Read 3	Cataract extraction, insertion of intraocular lens and trabeculectomy
Glaucoma	XE0BO	Read 3	Goniotomy
Glaucoma	XE0BP	Read 3	Goniopuncture
Glaucoma	XE0Kk	Read 3	Intraocular tension relief (& glaucoma)
Glaucoma	XE0Km	Read 3	(Iridectomy) or (trabeculectomy)
Glaucoma	XE0Ko	Read 3	(Iridotomy) or (trabeculotomy)
Glaucoma	XaE6n	Read 3	Viscocanulostomy
Glaucoma	XaEXv	Read 3	Revision of trabeculectomy
Glaucoma	XaEYc	Read 3	Resuturing of trabeculectomy
Glaucoma	XaKSk	Read 3	Adjustment of trabeculectomy suture

Disease	Code	Code type	Description
Glaucoma	XaKaz	Read 3	Trabeculectomy with intraoperative application of 5-fluorouracil
Glaucoma	XaKb1	Read 3	Trabeculectomy with intraoperative application of mitomycin
Glaucoma	XaKb3	Read 3	Trabeculectomy with beta-irradiation
Glaucoma	XaKb6	Read 3	Removal of glaucoma drainage tube
Glaucoma	XaKb8	Read 3	Removal of Molteno tube
Glaucoma	XaKbh	Read 3	Removal of releasable trabeculectomy suture
Glaucoma	XaKbp	Read 3	Adjustment of suture within Molteno tube
Glaucoma	XaL58	Read 3	Operations following glaucoma surgery
Glaucoma	XaL59	Read 3	Removal of releasable suture following glaucoma surgery
Glaucoma	XaL5A	Read 3	Laser suture lysis following glaucoma surgery
Glaucoma	XaL5B	Read 3	Other specified operations following glaucoma surgery
Glaucoma	XaL5C	Read 3	Operations following glaucoma surgery NOS
Glaucoma	XaL5m	Read 3	Needling of bleb following glaucoma surgery
Glaucoma	XaL5n	Read 3	Injection of bleb following glaucoma surgery
Glaucoma	XaL5o	Read 3	Revision of bleb NEC following glaucoma surgery
Glaucoma	XaXCJ	Read 3	Viscogonioplasty
Glaucoma	.7381	Read 3	Intraocular tension relief (& glaucoma)
Glaucoma	.7384	Read 3	(Iridectomy) or (trabeculectomy)
Glaucoma	.7385	Read 3	(Iridotomy) or (trabeculotomy)
Glaucoma	72556	Read 3	Revision of trabeculectomy
Glaucoma	72557	Read 3	Viscocanulostomy
Glaucoma	72564	Read 3	Viscogonioplasty
Glaucoma	72587	Read 3	Resuturing of trabeculectomy
Glaucoma	7259.	Read 3	Operations following glaucoma surgery
Glaucoma	72590	Read 3	Needling of bleb following glaucoma surgery
Glaucoma	72591	Read 3	Injection of bleb following glaucoma surgery
Glaucoma	72592	Read 3	Revision of bleb NEC following glaucoma surgery
Glaucoma	72593	Read 3	Removal of releasable suture following glaucoma surgery

Disease	Code	Code type	Description
Glaucoma	72594	Read 3	Laser suture lysis following glaucoma surgery
Glaucoma	7259y	Read 3	Other specified operations following glaucoma surgery
Glaucoma	7259z	Read 3	Operations following glaucoma surgery NOS
Glaucoma	F45..	Read 3	Glaucoma
Glaucoma	F45z.	Read 3	Glaucoma NOS
Glaucoma	F4H14	Read 3	Glaucomatous optic atrophy
Glaucoma	.F54.	Read 3	Glaucoma
Glaucoma	FyuG.	Read 3	Glaucoma
Glaucoma	1482.	Read 3	H/O: glaucoma
Glaucoma	.1482	Read 3	H/O: glaucoma
Glaucoma	F452.	Read 3	Glaucoma: [primary angle-closure] or [closed angle]
Glaucoma	F4520	Read 3	Unspecified primary angle-closure glaucoma
Glaucoma	F4521	Read 3	Intermittent angle-closure glaucoma
Glaucoma	F4524	Read 3	Primary angle-closure glaucoma residual stage
Glaucoma	F452z	Read 3	Primary angle-closure glaucoma NOS
Glaucoma	XE2a8	Read 3	Primary angle-closure glaucoma
Glaucoma	.F543	Read 3	Primary angle-closure glaucoma
Glaucoma	F4511	Read 3	Primary open-angle glaucoma
Glaucoma	F4512	Read 3	Low tension glaucoma
Glaucoma	XaF9D	Read 3	Primary open-angle glaucoma with narrow angles
Glaucoma	.F542	Read 3	Primary open-angle glaucoma
Glaucoma	.F544	Read 3	Low tension glaucoma
Glaucoma	F45y2	Read 3	Low tension glaucoma
Glaucoma	F4042	Read 3	(Blind hypertensive eye) or (glaucoma absolute)
Glaucoma	F4421	Read 3	Posner-Schlossman syndrome
Glaucoma	F4513	Read 3	Pigmentary glaucoma
Glaucoma	F453.	Read 3	Steroid-induced glaucoma
Glaucoma	F4530	Read 3	Steroid-induced glaucoma glaucomatous stage
Glaucoma	F4531	Read 3	Steroid-induced glaucoma residual stage

Disease	Code	Code type	Description
Glaucoma	F453z	Read 3	Steroid-induced glaucoma NOS
Glaucoma	F454.	Read 3	Glaucoma due to disease EC
Glaucoma	F4540	Read 3	Glaucoma due to chamber angle anomaly
Glaucoma	F4541	Read 3	Glaucoma due to iris anomaly
Glaucoma	F4542	Read 3	Glaucoma due to other anterior segment anomaly
Glaucoma	F4543	Read 3	Glaucoma due to systemic syndrome
Glaucoma	F4544	Read 3	Glaucoma in endocrine, nutritional and metabolic diseases
Glaucoma	F454z	Read 3	Glaucoma due to disease NOS
Glaucoma	F455.	Read 3	Glaucoma with lens disorder
Glaucoma	F4550	Read 3	Phacolytic glaucoma
Glaucoma	F4551	Read 3	Pseudoexfoliation glaucoma
Glaucoma	F455z	Read 3	Glaucoma associated with disorders of the lens NOS
Glaucoma	F456.	Read 3	Glaucoma associated with other ocular disorders
Glaucoma	F4560	Read 3	Glaucoma due to unspecified ocular disorder
Glaucoma	F4561	Read 3	Secondary angle-closure glaucoma with pupil block
Glaucoma	F4562	Read 3	Glaucoma with ocular inflammation
Glaucoma	F4563	Read 3	Glaucoma due to ocular vascular disorder
Glaucoma	F4564	Read 3	Glaucoma due to ocular tumour &/or cyst
Glaucoma	F4565	Read 3	Glaucoma due to ocular trauma
Glaucoma	F456z	Read 3	Glaucoma associated with other ocular disorders NOS
Glaucoma	F45y.	Read 3	Other specified forms of glaucoma
Glaucoma	F45y0	Read 3	Hypersecretion glaucoma
Glaucoma	F45y1	Read 3	Glaucoma due to raised episcleral venous pressure
Glaucoma	F45yz	Read 3	Other specified glaucoma NOS
Glaucoma	FyuG0	Read 3	[X]Other glaucoma
Glaucoma	FyuG1	Read 3	[X]Glaucoma in endocrine, nutritional and metabolic diseases classified elsewhere
Glaucoma	FyuG2	Read 3	[X]Glaucoma in other diseases classified elsewhere
Glaucoma	X00ee	Read 3	Mixed open and angle-closure glaucoma

Disease	Code	Code type	Description
Glaucoma	X00ef	Read 3	Absolute glaucoma
Glaucoma	X00eg	Read 3	Iatrogenic glaucoma
Glaucoma	X00eh	Read 3	Iatrogenic angle-closure glaucoma
Glaucoma	X00ei	Read 3	Glaucoma and corneal anomaly
Glaucoma	X00ek	Read 3	Chandler iris naevus syndrome
Glaucoma	X00el	Read 3	Phacogenic glaucoma
Glaucoma	X00em	Read 3	Neovascular glaucoma
Glaucoma	X00en	Read 3	Glaucoma with intraocular haemorrhage
Glaucoma	X00eo	Read 3	Ghost cell glaucoma
Glaucoma	X00eq	Read 3	Glaucoma due to contusion injury
Glaucoma	X00er	Read 3	Angle recession glaucoma
Glaucoma	X00es	Read 3	Glaucoma following surgery
Glaucoma	X00et	Read 3	Ciliary block glaucoma
Glaucoma	XE15r	Read 3	Blind hypertensive eye
Glaucoma	XE16F	Read 3	Glaucoma due to ocular tumour or cyst
Glaucoma	XM1QA	Read 3	Glaucoma due to ocular cyst
Glaucoma	Xa3fE	Read 3	Secondary/other glaucoma
Glaucoma	Xa9BT	Read 3	Secondary glaucoma
Glaucoma	XaEVp	Read 3	Uveitic glaucoma
Glaucoma	XaF9I	Read 3	Secondary open-angle glaucoma
Glaucoma	XaF9J	Read 3	Secondary angle-closure glaucoma
Glaucoma	XaFao	Read 3	Secondary angle-closure glaucoma - synechial
Glaucoma	XaFap	Read 3	Secondary angle-closure glaucoma with rubeosis
Glaucoma	XaI97	Read 3	Glaucoma due to silicon oil
Glaucoma	.F54Z	Read 3	Secondary/other glaucoma
Glaucoma	F448.	Read 3	Pigmentary glaucoma
Glaucoma	F4566	Read 3	Neovascular glaucoma
Glaucoma	F4522	Read 3	Acute angle-closure glaucoma
Glaucoma	F4523	Read 3	Chronic angle-closure glaucoma

Disease	Code	Code type	Description
Glaucoma	F4631	Read 3	Glaucomatous subcapsular flecks
Glaucoma	XaEVW	Read 3	Acute-on-chronic glaucoma
Glaucoma	F4501	Read 3	Open-angle glaucoma - borderline
Glaucoma	F451.	Read 3	Open-angle glaucoma
Glaucoma	F4510	Read 3	Unspecified open-angle glaucoma
Glaucoma	F4515	Read 3	Open-angle glaucoma residual stage
Glaucoma	F451z	Read 3	Open-angle glaucoma NOS
Hypertension	1065	Data coding 6	hypertension
Hypertension	1072	Data coding 6	essential hypertension
Hypertension	1073	Data coding 6	gestational hypertension/pre-eclampsia
Hypertension	I10	ICD-10	Essential (primary) hypertension
Hypertension	I11	ICD-10	Hypertensive heart disease
Hypertension	I12	ICD-10	Hypertensive renal disease
Hypertension	I13	ICD-10	Hypertensive heart and renal disease
Hypertension	I110	ICD-10	Hypertensive heart disease with (congestive) heart failure
Hypertension	I119	ICD-10	Hypertensive heart disease without (congestive) heart failure
Hypertension	I120	ICD-10	Hypertensive renal disease with renal failure
Hypertension	I129	ICD-10	Hypertensive renal disease without renal failure
Hypertension	I130	ICD-10	Hypertensive heart and renal disease with (congestive) heart failure
Hypertension	I131	ICD-10	Hypertensive heart and renal disease with renal failure
Hypertension	I132	ICD-10	Hypertensive heart and renal disease with both (congestive) heart failure and renal failure
Hypertension	I139	ICD-10	Hypertensive heart and renal disease, unspecified
Hypertension	I15	ICD-10	Secondary hypertension
Hypertension	I150	ICD-10	Renovascular hypertension
Hypertension	I151	ICD-10	Hypertension secondary to other renal disorders
Hypertension	I152	ICD-10	Hypertension secondary to endocrine disorders

Disease	Code	Code type	Description
Hypertension	I158	ICD-10	Other secondary hypertension
Hypertension	I159	ICD-10	Secondary hypertension, unspecified
Hypertension	4010	ICD-9	MALIGNANT ESSENTIAL HYPERTENSION
Hypertension	4011	ICD-9	BENIGN ESSENTIAL HYPERTENSION
Hypertension	4019	ICD-9	ESSENTIAL HYPERTENSION NOT SPECIFIED
Hypertension	4020	ICD-9	MALIGNANT HYPERTENSIVE HEART DISEASE
Hypertension	4021	ICD-9	BENIGN HYPERTENSIVE HEART DISEASE
Hypertension	4029	ICD-9	HYPERTENSIVE HEART DISEASE NOT SPECIFIED
Hypertension	4030	ICD-9	MALIGNANT HYPERTENSIVE RENAL DISEASE
Hypertension	4031	ICD-9	BENIGN HYPERTENSIVE RENAL DISEASE
Hypertension	4039	ICD-9	HYPERTENSIVE RENAL DISEASE NOT SPECIFIED
Hypertension	4040	ICD-9	MALIGNANT HYPERTENSIVE HEART AND RENAL DISEASE
Hypertension	4041	ICD-9	BENIGN HYPERTENSIVE HEART AND RENAL DISEASE
Hypertension	4049	ICD-9	HYPERTENSIVE HEART AND RENAL DISEASE NOT SPECIFIED
Hypertension	4050	ICD-9	SPECIFIED AS MALIGNANT
Hypertension	4051	ICD-9	SPECIFIED AS BENIGN
Hypertension	4059	ICD-9	NOT SPECIFIED AS MALIGNANT OR BENIGN
Hypertension	14A2.	Read 2	H/O: hypertension
Hypertension	21261	Read 2	Hypertension resolved
Hypertension	212K.	Read 2	Hypertension resolved
Hypertension	9OI9.	Read 2	Hypertens.monitor deleted
Hypertension	6624.	Read 2	Borderline hyperten:yearly obs
Hypertension	6627.	Read 2	Good hypertension control
Hypertension	6628.	Read 2	Poor hypertension control
Hypertension	662b.	Read 2	Moderate hypertension control
Hypertension	662c.	Read 2	Hypertension six month review
Hypertension	662d.	Read 2	Hypertension annual review
Hypertension	662F.	Read 2	Hypertension treatm. started

Disease	Code	Code type	Description
Hypertension	662G.	Read 2	Hypertensive treatm.changed
Hypertension	662O.	Read 2	On treatment for hypertension
Hypertension	662r.	Read 2	Trial withdrawal of antihypertensive therapy
Hypertension	7Q01.	Read 2	High cost hypertension drugs
Hypertension	8B26.	Read 2	Antihypertensive therapy
Hypertension	8BL0.	Read 2	Patient on maximal tolerated antihypertensive therapy
Hypertension	8I3N.	Read 2	Hypertension treatment refused
Hypertension	F4213	Read 2	Hypertensive retinopathy
Hypertension	G2...	Read 2	Hypertensive disease
Hypertension	G20..	Read 2	Essential hypertension
Hypertension	G200.	Read 2	Malignant essential hypertension
Hypertension	G201.	Read 2	Benign essential hypertension
Hypertension	G202.	Read 2	Systolic hypertension
Hypertension	G203.	Read 2	Diastolic hypertension
Hypertension	G20z.	Read 2	Essential hypertension NOS
Hypertension	G21..	Read 2	Hypertensive heart disease
Hypertension	G210.	Read 2	Malignant hypertensive heart disease
Hypertension	G2100	Read 2	Malignant hypertensive heart disease without CCF
Hypertension	G2101	Read 2	Malignant hypertensive heart disease with CCF
Hypertension	G211.	Read 2	Benign hypertensive heart disease
Hypertension	G2110	Read 2	Benign hypertensive heart disease without CCF
Hypertension	G2111	Read 2	Benign hypertensive heart disease with CCF
Hypertension	G21z.	Read 2	Hypertensive heart disease NOS
Hypertension	G21z0	Read 2	Hypertensive heart disease NOS without CCF
Hypertension	G21z1	Read 2	Hypertensive heart disease NOS with CCF
Hypertension	G21zz	Read 2	Hypertensive heart disease NOS
Hypertension	G22..	Read 2	Hypertensive renal disease
Hypertension	G220.	Read 2	Malignant hypertensive renal disease
Hypertension	G221.	Read 2	Benign hypertensive renal disease

Disease	Code	Code type	Description
Hypertension	G222.	Read 2	Hypertensive renal disease with renal failure
Hypertension	G22z.	Read 2	Hypertensive renal disease NOS
Hypertension	G23..	Read 2	Hypertensive heart and renal disease
Hypertension	G230.	Read 2	Malignant hypertensive heart and renal disease
Hypertension	G231.	Read 2	Benign hypertensive heart and renal disease
Hypertension	G232.	Read 2	Hypertensive heart&renal dis wth (congestive) heart failure
Hypertension	G233.	Read 2	Hypertensive heart and renal disease with renal failure
Hypertension	G234.	Read 2	Hyperten heart&renal dis+both(congestv)heart and renal fail
Hypertension	G23z.	Read 2	Hypertensive heart and renal disease NOS
Hypertension	G2y..	Read 2	Other specified hypertensive disease
Hypertension	G2z..	Read 2	Hypertensive disease NOS
Hypertension	G672.	Read 2	Hypertensive encephalopathy
Hypertension	Gyu2.	Read 2	[X]Hypertensive diseases
Hypertension	L122.	Read 2	Other pre-existing hypertension in preg/childbirth/puerp
Hypertension	L1220	Read 2	Other pre-existing hypertension in preg/childb/puerp unspec
Hypertension	L1221	Read 2	Other pre-existing hypertension in preg/childb/puerp - deliv
Hypertension	L1223	Read 2	Other pre-exist hypertension in preg/childb/puerp-not deliv
Hypertension	L122z	Read 2	Other pre-existing hypertension in preg/childb/puerp NOS
Hypertension	L127.	Read 2	Pre-eclampsia or eclampsia with pre-existing hypertension
Hypertension	L127z	Read 2	Pre-eclampsia or eclampsia + pre-existing hypertension NOS
Hypertension	L128.	Read 2	Pre-exist hypertension compl preg childbirth and puerperium
Hypertension	L1280	Read 2	Pre-exist hyperten heart dis compl preg childbth+puerperium
Hypertension	L1282	Read 2	Pre-exist 2ndry hypertens comp preg childbth and puerperium
Hypertension	TJC7.	Read 2	Adverse reaction to other antihypertensives
Hypertension	TJC7z	Read 2	Adverse reaction to antihypertensives NOS
Hypertension	U60C5	Read 2	[X]Oth antihyperten drug caus advers eff in therap use, NEC
Hypertension	61462	Read 2	Hypertension induced by oral contraceptive pill
Hypertension	G24..	Read 2	Secondary hypertension

Disease	Code	Code type	Description
Hypertension	G240.	Read 2	Secondary malignant hypertension
Hypertension	G2400	Read 2	Secondary malignant renovascular hypertension
Hypertension	G240z	Read 2	Secondary malignant hypertension NOS
Hypertension	G241.	Read 2	Secondary benign hypertension
Hypertension	G2410	Read 2	Secondary benign renovascular hypertension
Hypertension	G241z	Read 2	Secondary benign hypertension NOS
Hypertension	G244.	Read 2	Hypertension secondary to endocrine disorders
Hypertension	G24z.	Read 2	Secondary hypertension NOS
Hypertension	G24z0	Read 2	Secondary renovascular hypertension NOS
Hypertension	G24z1	Read 2	Hypertension secondary to drug
Hypertension	G24zz	Read 2	Secondary hypertension NOS
Hypertension	Gyu21	Read 2	[X]Hypertension secondary to other renal disorders
Hypertension	14A2.	Read 3	H/O: hypertension
Hypertension	21261	Read 3	Hypertension resolved
Hypertension	6624.	Read 3	Borderline hyperten:yearly obs
Hypertension	6627.	Read 3	Good hypertension control
Hypertension	6628.	Read 3	Poor hypertension control
Hypertension	XaIy8	Read 3	Moderate hypertension control
Hypertension	XaIyD	Read 3	Hypertension six month review
Hypertension	XaIyE	Read 3	Hypertension annual review
Hypertension	662F.	Read 3	Hypertension treatm. started
Hypertension	662G.	Read 3	Hypertensive treatm.changed
Hypertension	Xa8HD	Read 3	On treatment for hypertension
Hypertension	XaNFs	Read 3	Trial withdrawal of antihypertensive therapy
Hypertension	XaM5f	Read 3	High cost hypertension drugs
Hypertension	XaBLq	Read 3	Antihypertensive therapy
Hypertension	XaJ5h	Read 3	Patient on maximal tolerated antihypertensive therapy
Hypertension	XaIyC	Read 3	Hypertension treatment refused
Hypertension	F4211	Read 3	Hypertensive retinopathy

Disease	Code	Code type	Description
Hypertension	G2...	Read 3	Hypertensive disease
Hypertension	XE0Uc	Read 3	Essential hypertension
Hypertension	XM02V	Read 3	Raised blood pressure
Hypertension	G200.	Read 3	Malignant essential hypertension
Hypertension	G201.	Read 3	Benign essential hypertension
Hypertension	G202.	Read 3	Systolic hypertension
Hypertension	XSDSb	Read 3	Diastolic hypertension
Hypertension	XE0Ud	Read 3	Essential hypertension NOS
Hypertension	XE0Ub	Read 3	Hypertension
Hypertension	G21..	Read 3	Hypertensive heart disease
Hypertension	G210.	Read 3	Malignant hypertensive heart disease
Hypertension	G2100	Read 3	Malignant hypertensive heart disease without congestive cardiac failure
Hypertension	G2101	Read 3	Malignant hypertensive heart disease with congestive cardiac failure
Hypertension	G211.	Read 3	Benign hypertensive heart disease
Hypertension	G2110	Read 3	Benign hypertensive heart disease without congestive cardiac failure
Hypertension	G2111	Read 3	Benign hypertensive heart disease with congestive cardiac failure
Hypertension	G21z.	Read 3	Hypertensive heart disease NOS
Hypertension	XE0Ue	Read 3	Hypertensive heart disease NOS without congestive cardiac failure
Hypertension	XM1Qp	Read 3	Cardiomegaly - hypertensive
Hypertension	G21z1	Read 3	Hypertensive heart disease NOS with congestive cardiac failure
Hypertension	XE0Uf	Read 3	Hypertensive renal disease
Hypertension	G220.	Read 3	Malignant hypertensive renal disease
Hypertension	G221.	Read 3	Benign hypertensive renal disease
Hypertension	G222.	Read 3	Hypertensive renal disease with renal failure
Hypertension	XE0Ug	Read 3	Hypertensive renal disease NOS
Hypertension	G23..	Read 3	Hypertensive heart and renal disease

Disease	Code	Code type	Description
Hypertension	G230.	Read 3	Malignant hypertensive heart and renal disease
Hypertension	G231.	Read 3	Benign hypertensive heart and renal disease
Hypertension	G232.	Read 3	Hypertensive heart and renal disease with (congestive) heart failure
Hypertension	G233.	Read 3	Hypertensive heart and renal disease with renal failure
Hypertension	G234.	Read 3	Hypertensive heart and renal disease with both (congestive) heart failure and renal failure
Hypertension	G23z.	Read 3	Hypertensive heart and renal disease NOS
Hypertension	G2y..	Read 3	Other specified hypertensive disease
Hypertension	G2z..	Read 3	Hypertensive disease NOS
Hypertension	XE0VM	Read 3	Hypertensive encephalopathy
Hypertension	Gyu2.	Read 3	[X]Hypertensive diseases
Hypertension	L122.	Read 3	Other pre-existing hypertension in preg/childbirth/puerp
Hypertension	L1220	Read 3	Other pre-existing hypertension complicating pregnancy, childbirth and the puerperium unspecified
Hypertension	L1221	Read 3	Other pre-existing hypertension complicating pregnancy, childbirth and the puerperium - delivered
Hypertension	L1223	Read 3	Other pre-existing hypertension complicating pregnancy, childbirth and the puerperium - not delivered
Hypertension	L122z	Read 3	Other pre-existing hypertension complicating pregnancy, childbirth and the puerperium NOS
Hypertension	L127.	Read 3	Pre-eclampsia or eclampsia with pre-existing hypertension
Hypertension	L127z	Read 3	Pre-eclampsia or eclampsia with pre-existing hypertension NOS
Hypertension	L128.	Read 3	Pre-existing hypertension complicating pregnancy, childbirth and puerperium
Hypertension	L1280	Read 3	Pre-existing hypertensive heart disease complicating pregnancy, childbirth and the puerperium
Hypertension	L1282	Read 3	Pre-existing secondary hypertension complicating pregnancy, childbirth and puerperium
Hypertension	TJC7.	Read 3	Adverse reaction to other antihypertensives
Hypertension	TJC7z	Read 3	Adverse reaction to antihypertensives NOS
Hypertension	U60C5	Read 3	[X]Other antihypertensive drugs causing adverse effects in therapeutic use, not elsewhere classified

Disease	Code	Code type	Description
Hypertension	61462	Read 3	Hypertension induced by oral contraceptive pill
Hypertension	G24..	Read 3	Secondary hypertension
Hypertension	G240.	Read 3	Malignant secondary hypertension
Hypertension	G2400	Read 3	Secondary malignant renovascular hypertension
Hypertension	G240z	Read 3	Secondary malignant hypertension NOS
Hypertension	G241.	Read 3	Secondary benign hypertension
Hypertension	G2410	Read 3	Secondary benign renovascular hypertension
Hypertension	G241z	Read 3	Secondary benign hypertension NOS
Hypertension	G244.	Read 3	Hypertension secondary to endocrine disorders
Hypertension	G24z.	Read 3	Secondary hypertension NOS
Hypertension	G24z0	Read 3	Secondary renovascular hypertension NOS
Hypertension	G24z1	Read 3	Hypertension secondary to drug
Hypertension	Gyu21	Read 3	[X]Hypertension secondary to other renal disorders

sTable 3. Participant characteristics comparing individuals who had gradable retinal images (optical coherence tomography or colour photos) in both eyes with those where retinal images were either unavailable or ungradable in at least one eye. Characteristics were ascertained from self-reported data at study baseline and linked electronic health care records data (primary care, hospital episode statistics, death register) up until the time of retinal imaging, or 1st January 2010 for participants who did not undergo imaging. Continuous variables are summarised by mean (standard deviation) and compared using the Student's t-test and One-Way ANOVA for two and more than two groups respectively. Categorical variables are summarised by number (percentage) and compared using Pearson's Chi-squared test. Abbreviations: AMD = age-related macular degeneration, MA = microaneurysm.

Characteristic	No, N = 56,308	Yes, N = 12,206	p-value
**Age**	56.9 (8.2)	59.2 (7.7)	**<0.001**
**Sex**			**<0.001**
Female	31,123 (55.3%)	6,107 (50.0%)	
Male	25,185 (44.7%)	6,099 (50.0%)	
**Ethnic background**			**<0.001**
White	51,135 (91.4%)	10,461 (86.5%)	
Mixed	481 (0.9%)	119 (1.0%)	
Asian or Asian British	1,597 (2.9%)	625 (5.2%)	
Black or Black British	1,633 (2.9%)	580 (4.8%)	
Chinese	237 (0.4%)	75 (0.6%)	
Other ethnic group	838 (1.5%)	237 (2.0%)	
Missing	387	109	
**Townsend Deprivation Index**	-1.05 (2.98)	-0.84 (3.09)	**<0.001**
Missing	73	11	
**On insulin**	540 (1.0%)	198 (1.6%)	**<0.001**
**On antihypertensive medication**	11,113 (19.7%)	3,144 (25.8%)	**<0.001**
**On cholesterol-lowering medication**	9,880 (17.5%)	2,808 (23.0%)	**<0.001**
**Diabetes diagnosis**	2,914 (5.2%)	912 (7.5%)	**<0.001**
**Hypertension diagnosis**	14,749 (26.2%)	3,975 (32.6%)	**<0.001**
**Age-related macular degeneration diagnosis**	205 (0.4%)	66 (0.5%)	**0.005**
**Glaucoma diagnosis**	830 (1.5%)	298 (2.4%)	**<0.001**
**Diabetic retinopathy diagnosis**	500 (0.9%)	178 (1.5%)	**<0.001**

sTable 4. Summary of all graded retinal features at the person level and by eye (see supplementary table 1 for variable definitions and abbreviations). VisitDate is summarised as a range, continuous variables by mean (standard deviation) and categorical variables by number. Person level summaries of categorical variables describe the number of individuals where at least one eye has that feature recorded.

	Person	Right eye	Left eye
**VisitDate**	2009-12-07 to 2010-07-21	—	—
**CupDiscRatioComparison**	0.04 (0.05)	—	—
**COL_Available**	68,504	67,982	67,610
**OCT_Available**	68,497	67,922	67,578
**COL_Quality**			
CG	10,436	6,962	7,370
Fair	47,575	39,191	39,639
Good	9,704	8,046	6,259
Poor	21,223	13,783	14,342
**OCT_Quality**			
CG	905	538	548
Fair	53,176	47,029	47,623
Good	18,403	15,244	14,840
Poor	7,264	5,111	4,567
**AbnormalitiesPresent**	24,733	18,380	18,140
**AMD**	15,176	10,507	10,472
**COL_IfAMD**			
Drusen	9,881	6,858	6,471
Exud	63	39	35
GA	17	10	11
Pigment	581	296	356
**COL_DrusenInsideGrid**	7,544	5,059	4,754
**COL_SevereDrusen**			
CG	2	1	1
GreaterThan125	1,586	992	894
LessThan63	3,082	1,842	1,784
SixtyThreeTo125	3,594	2,224	2,076
**COL_DrusenOutsideGrid**	5,864	4,034	3,835
**COL_ReticularDrusen**			

	Person	Right eye	Left eye
CG	10	8	4
No	9,842	6,814	6,429
Yes	41	35	36
**OCT_DrusenPresent**	6,528	4,237	4,151
**OCT_SubretDrusen**	8,713	5,602	5,974
**OCT_FocalAtrophy**	432	222	269
**OCT_CentralSubfieldThickness**	—	245 (41)	247 (40)
**COL_RetinalHaemorrhage**	740	488	406
**COL_SignsOfDR**	264	209	193
**COL_DRFeatures**			
CWS	86	67	53
Ex	147	109	90
FP	10	4	8
HMA	264	209	193
IRMA	19	12	11
LS	62	41	56
MBH	8	5	4
NVD	9	3	7
NVE	7	4	3
PF	1	0	1
PVH	1	0	1
VB	6	4	4
VL	9	6	4
**COL_VisibleLaserTreatment**	99	75	67
**OCT_HyporeflectiveIntraretinalSpaces**	929	551	575
**COL_CupDiscRatio**	—	0.41 (0.10)	0.41 (0.09)
**COL_HaemorrhageOnDisc**	282	176	120
**COL_IfODSuspicious**			
Both (IRT AND Notch)	29	18	15
IRT	152	91	79

	Person	Right eye	Left eye
Notch	53	22	34
**Occlusion**	153	88	69
**IfOcclusion**			
BRAO	13	9	4
BRVO	102	62	41
CRAO	1	1	0
CRVO	40	17	24
**EvidenceOfERM**	7,996	5,583	5,890
**IfERMStructuralChanges**	1,550	906	922
**OCT_EvidenceOfVMA**	45,266	38,110	39,120
**OCT_IfVMACategories**			
WithoutTraction	44,954	37,770	38,755
WithTraction	617	340	365
**Other**	4,593	2,932	2,747
**IfOtherMerged**			
Abnormal foveal contour	158	96	109
AH	89	46	52
Angioid streaks	1	1	1
Chorioretinal changes outside macula	47	24	25
Chorioretinal changes within macula	57	30	29
Choroidal rupture	8	0	8
Coloboma	4	0	4
Congenital loop on disc	10	7	4
Cotton wool spot	40	23	18
CSCR	228	125	144
Dragged disc	2	1	2
Dragged macula	1	1	0
Embolus	8	5	5
Exudate	28	14	15
Fibroproliferation	17	10	7

	Person	Right eye	Left eye
Focal inflammatory changes	17	9	13
Hypoplastic disc	6	4	5
Idiopathic SPED	100	54	56
Inherited retinal disease	41	41	40
Inner retinal changes	205	113	130
MacTel	5	5	5
MD	290	241	216
MH	312	174	168
MO	64	46	40
Morning glory syndrome	1	1	0
Myelinated nerve fibres	104	57	63
Naev	1,458	811	695
Non-AMD neovascularisation	2	2	0
Optic disc drusen	19	14	12
Optic disc pallor	5	3	3
Optic disc pit	8	2	6
Outer retinal changes	617	371	359
Pigmented lesion outside macula	132	74	59
Pigmented lesion within macula	124	75	55
Pigmented spot outside macula	101	48	56
Pigmented spot within macula	115	82	45
RD	34	23	12
Retinal folds	40	31	31
Retinal hamartoma	2	1	1
Retinal ischaemia	1	1	0
SFCS	46	24	27
Swollen optic disc	29	24	21
Vascular changes	61	39	39
Vessel tortuosity	163	147	137
Vitelliform macular dystrophy	73	57	54

	Person	Right eye	Left eye
Vitreous changes	35	22	18
